# Biomimetic Analysis of Neurotransmitters for Disease Diagnosis through Light‐Driven Nanozyme Sensor Array and Machine Learning

**DOI:** 10.1002/advs.202505333

**Published:** 2025-07-06

**Authors:** Kun Yu, Siyuan Lu, Kaiwen Qiu, Yuanzun Zhang, Aobing Sun, Shiqi Gong, Kai Wang, Xuzhu Gao, Xiangyu Xu, Hao Wang

**Affiliations:** ^1^ College of Medical Engineering Jining Medical University Jining 272067 P. R. China; ^2^ Lianyungang Clinical College Jiangsu University & The Second People's Hospital of Lianyungang Lianyungang 222006 P. R. China; ^3^ College of Basic Medicine Jining Medical University Jining 272067 P. R. China; ^4^ College of Clinical Medicine Jining Medical University Jining 272067 P. R. China; ^5^ School of Pharmaceutical Sciences & Institute of Materia Medica Science and Technology Innovation Center Shandong First Medical University & Shandong Academy of Medical Sciences Jinan 250062 P. R. China

**Keywords:** metal‐organic frameworks, nanozyme, neurological diseases, neurotransmitters, sensor array

## Abstract

Neurological diseases, including Alzheimer's disease, Parkinson's disease, and multiple sclerosis, pose a significant global health challenge due to their complex pathogenesis and widespread prevalence. These disorders are often associated with disruptions in neurotransmitter regulation, leading to progressive cognitive and motor impairments. Conventional diagnostic methods are time‐consuming and lack the sensitivity required for early‐stage detection. Herein, for the first time a novel photoresponsive nanozyme sensor array is presented that integrates metal‐organic frameworks (MOFs) and machine learning algorithms for the rapid, sensitive, and multiplexed detection of neurotransmitters. Wherein, Zn(II) meso‐Tetra(4‐carboxyphenyl)porphine (ZnTCPP) ‐based MOFs, with their large specific surface area, enhance the interaction between reactant substrates and catalytic active sites within the material, significantly improving response sensitivity. Additionally, light‐driven catalysis greatly accelerates the response speed of the nanozyme. Mimicking the mammalian olfactory system, the array responds to various neurotransmitters in a patterned manner, enabling accurate differentiation and quantification within minutes. It maintains high precision even in complex biological samples such as serum and cerebrospinal fluid. The biomimetic sensor can detect neurotransmitter signatures linked to neurological disorders, such as Alzheimer's disease. This platform offers significant potential for early diagnosis and continuous monitoring of neurological conditions.

## Graphical abstract

A novel photoresponsive nanozyme sensor array, combining metal‐organic frameworks (MOFs) with advanced machine learning algorithms, provides a breakthrough in rapid neurotransmitter detection and disease diagnostics. This sensor array is capable of accurately identifying neurotransmitter profiles associated with neurological disorders, offering significant promise for early diagnosis and monitoring. It sets a new standard for sensitivity and speed in the biomolecular detection field, expanding the potential for real‐time, rapid disease diagnostics.

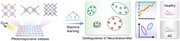

## 1 Introduction

Neurological diseases, including Alzheimer's disease (AD), Parkinson's disease (PD), and multiple sclerosis, *etc*., represent a significant global health burden, affecting millions of individuals worldwide. These conditions are characterized by the progressive degeneration of the nervous system, leading to cognitive decline, motor dysfunction, and a diminished quality of life.^[^ ^1^ ^]^ One of the central factors contributing to the onset and progression of neurological diseases is the dysregulation of neurotransmitters, such as dopamine, serotonin, and glutamate, which play pivotal roles in regulating mood, learning, memory, and motor control.^[^ ^2^ ^]^ The imbalance in the levels of these neurotransmitters has been closely linked to the pathophysiology of various neurological disorders, making their detection crucial for diagnosis and monitoring the progression of such diseases.^[^ ^3^ ^]^

Given the complex nature of neurological diseases and the involvement of multiple neurotransmitters, detecting neurotransmitters is fundamental to understanding disease mechanisms and facilitating early diagnosis.^[^ ^4^ ^]^ Traditional diagnostic methods for neurological diseases, such as clinical imaging, cerebrospinal fluid analysis, and neuropsychological assessments, often lack sensitivity and specificity in detecting early‐stage changes. Moreover, these methods can be invasive, time‐consuming, and costly, further hindering timely intervention.^[^ ^5^ ^]^ As a result, there is a growing demand for novel diagnostic platforms that can offer rapid, sensitive, and non‐invasive detection of neurological disease biomarkers, ideally in real‐time.^[^ ^6^ ^]^

Sensor arrays have emerged as powerful tools in the field of biomolecule detection, offering distinct advantages for the simultaneous monitoring of multiple analytes.^[^ ^7^ ^]^ Array‐based sensors utilize combinations of sensor elements to produce unique response patterns, akin to fingerprints, for different target molecules. This pattern recognition ability is particularly valuable in complex biological environments, where detecting multiple species simultaneously is often necessary.^[^ ^8^ ^]^ In the context of neurotransmitter detection, sensor arrays enable real‐time monitoring of various neurotransmitters, which is critical for understanding the interactions among signaling molecules in neurological diseases and for detecting disease. The use of sensor arrays in clinical applications has gained considerable attention due to their ability to perform multiplexed analyses with high throughput and reduced assay times.^[^ ^9^ ^]^

While conventional sensor arrays typically rely on organic or inorganic sensing materials, the integration of metal‐organic frameworks (MOFs) nanozyme into sensor arrays has opened up new possibilities.^[^ ^10^ ^]^ MOFs, known for their tunable porous structures, large surface areas, and catalytic activity, are ideal for enhancing sensor performance.^[^ ^11^ ^]^ In particular, MOF‐based nanozymes‐materials that mimic the activity of natural enzymes‐have shown promise in biosensing applications due to their superior catalytic efficiency, stability, and versatility.^[^ ^12^ ^]^ Unlike natural enzymes, MOF nanozymes exhibit greater robustness under diverse environmental conditions and can be engineered to catalyze specific reactions, making them highly suitable for biosensing.^[^ ^13^ ^]^ Remarkably, Zn(II) *meso*‐Tetra (4‐carboxyphenyl) porphine (ZnTCPP) based MOF nanozymes exhibit exceptional photoactivated oxidase‐like activity, owing to their broad UV‐visible absorption that efficiently harvests visible light to drive electron excitation, generating highly reactive electron‐hole pairs.^[^ ^14^ ^]^ Concurrently, the excited‐state electrons rapidly migrate to substrates via π‐π stacking or coordination interactions, mimicking the electron transfer pathways of natural oxidases while achieving enhanced catalytic turnover.^[^ ^15^ ^]^ Therefore, incorporating photo‐catalytic properties into MOF nanozymes enhances their utility in sensor arrays. The light‐driven reactions facilitated by these properties accelerate the oxidation of target molecules, such as neurotransmitters, generating rapid and measurable colorimetric signals. This capability is especially beneficial for real‐time detection, where speed and accuracy are critical. The ability to use light for controlling catalytic reactions also adds an extra layer of precision, enabling selective detection of target analytes in complex biological samples.^[^ ^16^ ^]^

**Scheme 1:**
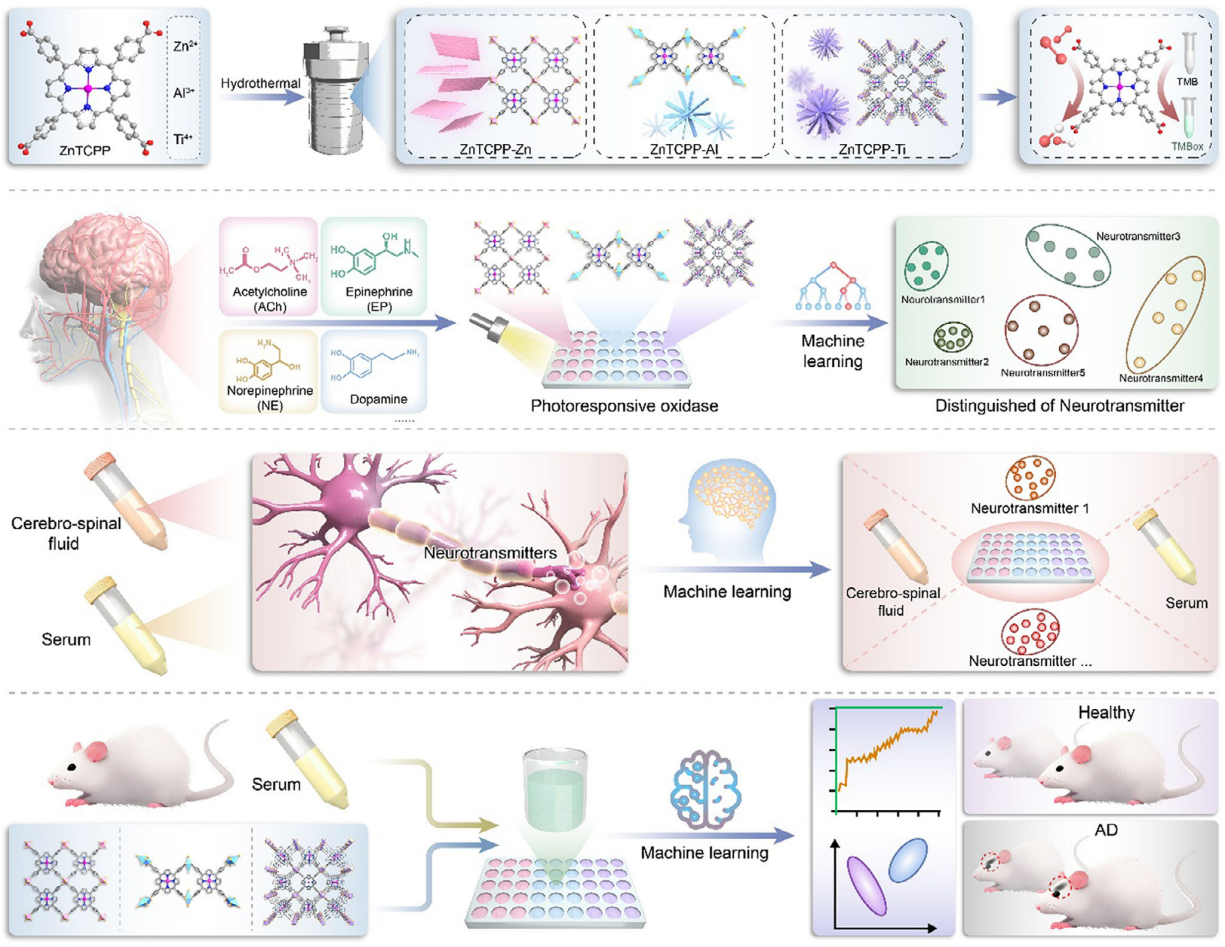
Schematic illustration of ZnTCPP MOF‐based light‐responsive nanozyme sensor array for neurological disease diagnosis.

In this study, the development of a photoresponsive nanozyme integrated with a machine learning‐based sensor array for the rapid and multiplexed detection of neurotransmitters. The three‐element sensor array was constructed using ZnTCPP‐based MOFs with different metal nodes. The catalytic activity of the nanozymes was regulated through diverse interactions between different types of neurotransmitters and both the surface and internal cavities of the ZnTCPP‐based MOFs. Simultaneously, neurotransmitters of various types influenced the catalytic processes of the nanozymes, affecting the generation of colored substrates. By collecting the unique colorimetric signals produced by these substrates and leveraging multiple machine learning algorithms, the array achieved qualitative and quantitative detection of different neurotransmitters. Moreover, the array demonstrated its capability to differentiate mixtures of neurotransmitters with varying proportions, delivering satisfactory results even in complex matrices such as serum and cerebrospinal fluid. Most importantly, the sensor array enabled accurate classification and reliable diagnosis of AD. Integrating MOF nanozymes into the array enhanced the sensor's sensitivity and selectivity, while the photo‐catalytic properties of the ZnTCPP‐based MOFs enabled the rapid quantification and classification of neurotransmitters and related diseases within minutes under light irradiation, without the need for any pretreatment (**Scheme** **1**). The MOF‐based photoresponsive nanozyme sensor array provides a fast, precise, and practical platform for the detection of multiple neurotransmitters, holding considerable promise for advancing the early diagnosis and continuous monitoring of neurological conditions.

## 2 Results and Discussion

### 2.1 Synthesis and Characterization of ZnTCPP MOFs

**Figure 1:**
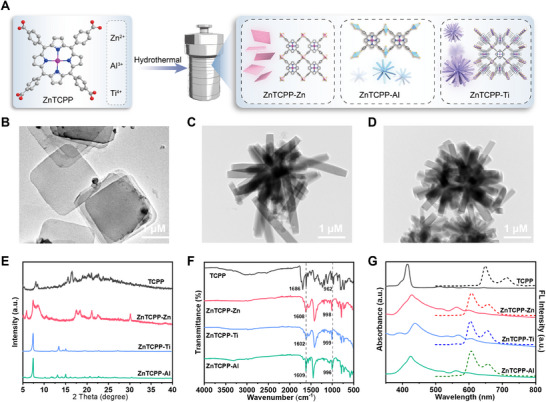
Synthesis and Characterization of ZnTCPP MOFs. A) Synthesis procedures of ZnTCPP‐Zn, ZnTCPP‐Ti, and ZnTCPP‐Al. TEM images of ZnTCPP‐Zn B), ZnTCPP‐Ti C), and ZnTCPP‐Al D). XRD pattern E), FT–IR spectra F), UV–vis absorption spectra, and emission spectra G) of TCPP, ZnTCPP‐Zn, ZnTCPP‐Ti, and ZnTCPP‐Al.

In this work, the synthesis of ZnTCPP was optimized based on prior literature (Scheme S1, Supporting Information). As shown in **Figure** **1**A, three types of ZnTCPP‐based MOFs (ZnTCPP‐Zn, ZnTCPP‐Ti, and ZnTCPP‐Al) were synthesized using ZnTCPP as the ligand through a simple dissolution‐thermal method. And Figure 1B reveals that ZnTCPP‐Zn exhibits a characteristic 2D layered architecture. Such structural configuration typically provides a substantially enlarged specific surface area, thereby enhancing the interaction between reactant substrates and catalytic active sites within the material. ZnTCPP‐Ti and ZnTCPP‐Al display rod‐like cluster structures (Figure 1C,D). Compared to the X‐ray diffraction (XRD) pattern of *meso*‐tetra(4‐carboxyphenyl)porphine (TCPP), ZnTCPP‐Zn, ZnTCPP‐Ti, and ZnTCPP‐Al show distinct characteristic peaks, indicating their good crystallinity (Figure 1E).^[^ ^15^, ^17^ ^]^ Further investigation using FT‐IR spectroscopy analyzed the functional groups of these MOFs. Specifically, the disappearance of the C = O stretching vibration peak at 1686 cm⁻¹ and the appearance of the M‐O bond near 1600 cm⁻¹ suggest that the carboxyl groups of ZnTCPP have undergone modification to form M‐O nodes through metal coordination (Figure 1F). Additionally, the 952 cm⁻¹ peak in TCPP corresponds to N‐H in‐plane vibrations, while new vibration peaks around 990 cm⁻¹ in ZnTCPP‐Zn, ZnTCPP‐Ti, and ZnTCPP‐Al indicate the presence of Zn(II) ions at the porphyrin ring center.^[^ ^18^ ^]^ Furthermore, the Q‐band of all ZnTCPP MOFs was reduced to two peaks due to the introduction of Zn(II) ions (Figure 1G), which increased the symmetry of the porphyrin, bringing energy levels closer together and resulting in fewer Q‐bands.^[^ ^19^ ^]^ Concurrently, a blue shift was observed in the fluorescence emission peaks of these ZnTCPP MOFs. This blue shift is attributed to the reduced electronic delocalization in the porphyrin molecules caused by metal‐ligand coordination, which raised the energy level of the lowest excited state and increased the energy gap for the S_0_→S_1_ transition. Finally, X‐ray photoelectron spectroscopy (XPS) confirmed the successful synthesis of ZnTCPP‐Zn, ZnTCPP‐Ti, and ZnTCPP‐Al (Figures S1–S3, Supporting Information).

### 2.2 Photoresponsive Oxidase‐Like Activities of ZnTCPP‐Zn, ZnTCPP‐Ti, and ZnTCPP‐Al

**Figure 2:**
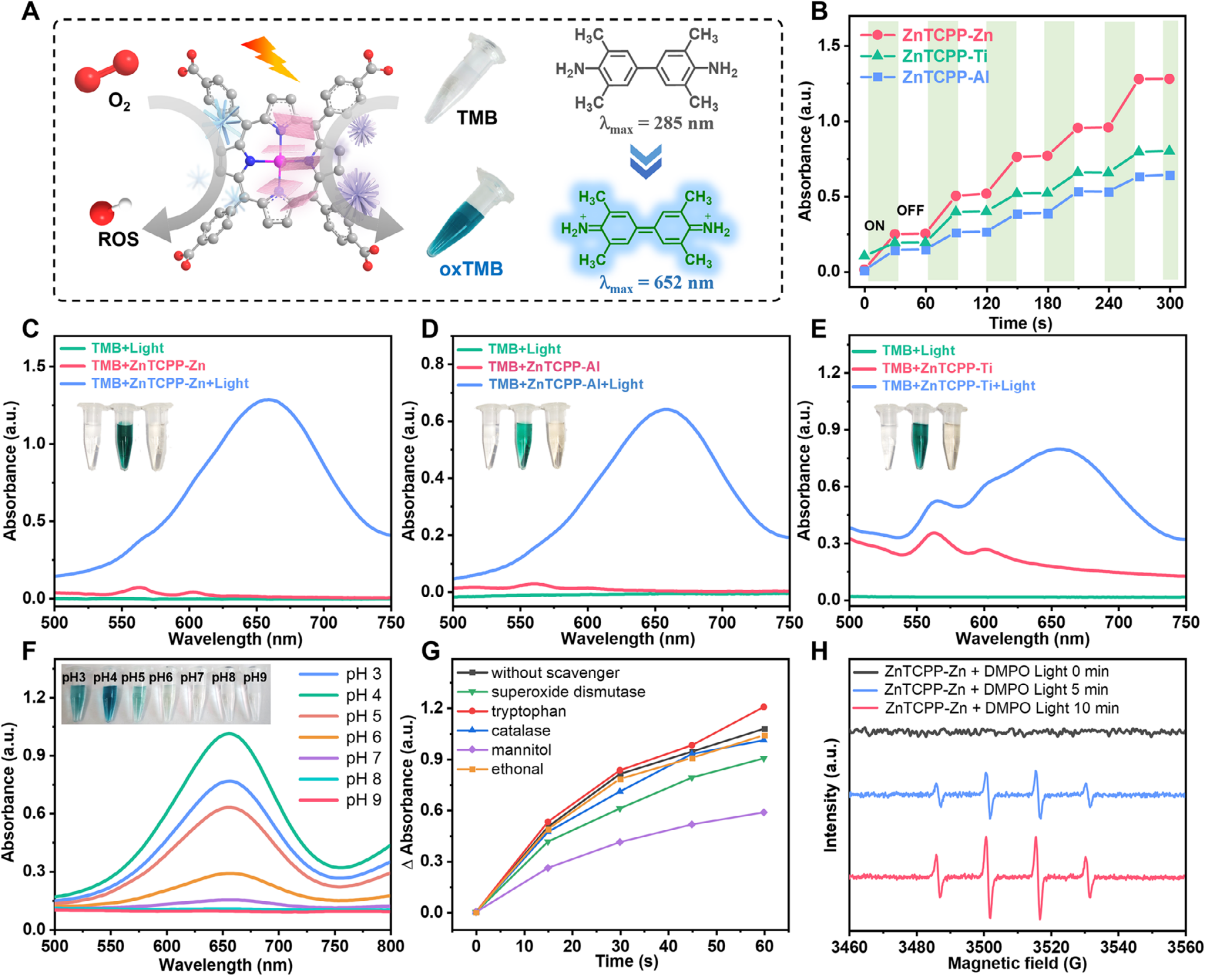
Photoresponsive oxidase‐like activities of ZnTCPP MOFs. A) Schematic illustration of the photoresponsive catalysis process. B) Staircaselike behavior of oxidase‐like activity when the light source was turned on and off. UV–vis absorption spectra of ZnTCPP‐Zn C), ZnTCPP‐Al D), and ZnTCPP‐Ti E) in 0.10 m acetate buffer containing TMB under light irradiation, MOFs + TMB (without light irradiation), and MOFs + TMB (under light irradiation). Inset: corresponding photograph of the three samples. The sequence from left to right is TMB + Light, TMB + MOFs + Light, and TMB + MOFs. F) Effects of pH on the oxidase‐mimicking activity of the ZnTCPP‐Zn. Inset: corresponding photograph of the seven samples. G) Effect of different scavengers on the catalytic oxidation of ZnTCPP‐Zn by the under light irradiation. H) EPR spectra of the ZnTCPP‐Zn and DMPO mixed solution before and after irradiation with Xe lamp (300 W) for 0, 5, and 10 min.

Subsequently, the catalytic oxidation of the typical oxidase colorimetric substrate 3,3′,5,5′‐tetramethylbenzidine (TMB) was monitored using UV‐Vis spectroscopy. Under light irradiation, the MOF catalyzed the oxidation of colorless TMB, producing a blue oxidized TMB (oxTMB) product (**Figure** **2**A). Further experiments using alternating periods of light exposure revealed a stepwise behavior in the oxidase‐like activity of ZnTCPP MOFs, indicating its light‐controllable oxidase‐like activity (Figure 2B). As shown in Figure 2C, the TMB sample alone did not exhibit a significant absorption peak under light, while a notable oxTMB absorption peak at 652 nm was observed for the ZnTCPP‐Zn and TMB mixture after light exposure. In contrast, the same sample without light exposure showed negligible absorption at 652 nm, further confirming the photo‐responsive activity of ZnTCPP‐Zn. Additionally, due to the incorporation of the same ZnTCPP photoactive ligand, ZnTCPP‐Ti and ZnTCPP‐Al also demonstrated excellent light‐responsive oxidase‐like activity (Figure 2D,E). To maximize the photo‐responsive oxidase‐like activity of MOF‐based nanozymes, we systematically explored the pH‐dependent activity profiles under illumination. As evidenced in Figure 2F, optimal catalytic performance was achieved at pH 4.0, demonstrating a distinct proton‐coupled electron transfer mechanism inherent to this photoresponsive system.

The oxidase‐like catalytic mechanism of photoactive ZnTCPP MOFs may involve multiple reactive oxygen species (ROS), including hydroxyl radicals (·OH), singlet oxygen (¹O_2_), hydrogen peroxide (H_2_O_2_), superoxide anions (O_2_·⁻), and photoinduced holes (h⁺). To elucidate the dominant catalytic pathways in ZnTCPP‐Zn, targeted scavenging experiments were conducted using established quenching agents: mannitol (·OH), tryptophan (¹O_2_), catalase (H_2_O_2_), superoxide dismutase (O_2_·⁻), and ethanol (h⁺). As demonstrated in Figure 2G, the oxidase‐like activity exhibited ·OH scavenging (45.8% activity loss with mannitol), while O_2_·⁻ depletion via SOD induced small inhibition (16.1% reduction). Intriguingly, neither tryptophan nor catalase caused statistically significant attenuation. Notably, ethanol‐mediated hole quenching paradoxically enhanced TMB oxidation by a small margin, suggesting that suppressed electron‐hole recombination may facilitate interfacial charge transfer kinetics. Additionally, terephthalic acid (TA) was employed as a fluorescent probe for ·OH, which forms 2‐hydroxyterephthalate (emission peak at≈420 nm). As depicted in Figure S4 (Supporting Information), the fluorescence intensity increased with prolonged irradiation, confirming the time‐dependent accumulation of ·OH. Meanwhile, the enhanced vibrations attributed to ·OH at 1440 cm^−1^ and C = N (oxTMB) at 1605 cm^−1^ in the in‐situ Raman spectra further confirmed our hypothesis. To mechanistically validate reactive oxygen species (ROS) participation in the catalytic cycle, electron paramagnetic resonance (EPR) spectroscopy with 5,5‐dimethyl‐1‐pyrroline N‐oxide (DMPO) as the spin‐trapping agent was employed. As unequivocally demonstrated in Figure 2H, ZnTCPP‐Zn photoresponsive nanozymes exhibited characteristic EPR signals corresponding to DMPO−OOH adducts, which are diagnostic of ·OH generation. Meanwhile, as the illumination time increases, the EPR signal gradually strengthens. These findings collectively demonstrate ·OH as the primary ROS mediator in the photocatalytic oxidase‐mimicking process. Moreover, steady‐state kinetics were employed to characterize the oxidase‐like activity of the ZnTCPP MOFs. Typical Michaelis‐Menten curves were obtained as shown in Figure S5 (Supporting Information), and kinetic parameters *v*_max_ and *K*_m_ were calculated based on the function *v* = *v*_max_ [*S*]/(*K*_m_ + [*S*]), where *v* represents the initial velocity, [*S*] the substrate concentration, and *v*_max_ and *K*_m_ denote the maximum reaction velocity and Michaelis constant, respectively. As shown in Table S1 (Supporting Information), ZnTCPP‐Zn exhibited the higher reaction rate and a lower *K*_m_ value, indicating a significant catalytic effect, which may be attributed to its unique 2D morphology. ZnTCPP‐Ti demonstrated the lowest *K*_m_ value, suggesting a higher affinity for TMB, while ZnTCPP‐Al showed the highest *v*_max_, indicating faster catalytic turnover. These differences enhance the array's capability for cross‐reactive pattern recognition, which is essential for accurate analyte discrimination. Additionally, the stability studies revealed exceptional preservation of catalytic activity across all three MOFs (ZnTCPP‐Zn, ZnTCPP‐Al, and ZnTCPP‐Ti). Additionally, the stability and reusability of the three MOFs were further investigated. As shown in Figure S6 (Supporting Information), these MOFs maintained >95% of their initial activity after 90 days of dry storage. Notably, even under aqueous conditions, the photocatalytic oxidase‐like activity remained at approximately 90%. In addition, we rigorously evaluated the sensors' cycling performance through four consecutive usage cycles. The data demonstrate that ZnTCPP‐Zn retained 87% of its initial activity after four cycles, while the more structurally robust ZnTCPP‐Al and ZnTCPP‐Ti maintained ∼90% activity (Figure S7, Supporting Information). The outstanding stability and repeatability of these materials are essential for high‐performance sensing systems.

### 2.3 The Catalytic Process and Density‐Functional Theory (DFT) Calculations of ZnTCPP MOF Nanozymes

**Figure 3:**
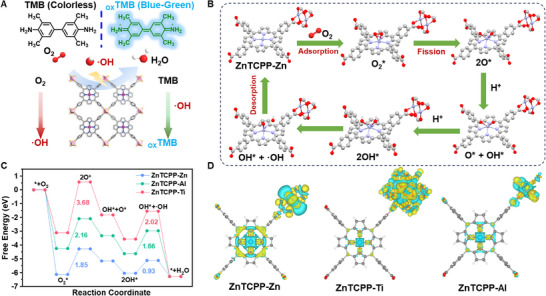
DFT calculations of nanozyme. A) Schematic representation of the oxidase‐mimicking activity of MOFs. B) Schematic of the proposed catalytic mechanism of MOFs. C) The free energy diagrams of MOFs during the catalytic process in an acidic environment. D) The charge density difference between ZnTCPP‐Zn, ZnTCPP‐Ti, and ZnTCPP‐Al. Yellow (blue) isosurfaces denote an accumulation (depletion) of 0.01 e Å^−3^ for electronic density.

The potential mechanisms underlying the oxidase‐mimicking activity of ZnTCPP MOFs were investigated by DFT calculations. **Figure** **3**A illustrated the schematic of the oxidase‐mimicking activity of nanozymes: O_2_ is catalyzed by MOF to produce ·OH, which further oxidizes the colorless TMB to generate blue oxTMB products. As shown in Figure 3B,C, the free O_2_ molecules readily adsorbed onto the Zn center of ZnTCPP, activing of O_2_ (*). Activated O_2_ (*) undergoes homogeneous cleavage at the Zn sites under photocatalysis, generating two O (*) bonded to the Zn sites. In this rate‐determining step, the energy barriers for ZnTCPP‐Ti, ZnTCPP‐Al, and ZnTCPP‐Zn are 3.68, 2.16, and 1.85 eV, respectively. Subsequently, protonated hydrogen atoms approach the two O (*) atoms, forming two OH (*) radicals, one of which absorbs energy to transform into a free ·OH radical. The energy barriers for this process are 2.02, 1.66, and 0.93 eV for ZnTCPP‐Ti, ZnTCPP‐Al, and ZnTCPP‐Zn, respectively. Finally, the protonated hydrogen atoms combine with the OH (*) radicals to produce H_2_O (*) and release H_2_O, restoring ZnTCPP to its initial state. Throughout the entire process, the free energy required for the rate‐determining step based on the Zn node in ZnTCPP‐Zn is significantly lower than that of the other two MOFs. As shown in Figure 3D, differential charge density calculations reveal that Zn‐TCPP‐Zn exhibits the highest electron transfer at the active Zn atom site. This indicates that the introduction of Zn cation groups significantly enhances the electron cloud density around the central Zn atom in the Zn‐TCPP framework. The increased electron transfer strengthens the interaction between the Zn atom and the Oxygen Reduction Reaction (ORR) intermediates, making adsorption and activation more efficient. Moreover, the enhanced electron supply capability of the Zn cation better supports the electron‐intensive steps of the ORR process. The introduction of Zn cations likely modulates the electronic state of the central Zn atom in Zn‐TCPP, optimizing the adsorption strength of ORR intermediates to achieve a balanced state—neither too strong (hindering desorption) nor too weak (impeding reaction progression). Such optimization facilitates the rapid adsorption and desorption of intermediate species, avoiding reaction bottlenecks. The reduced free energy barrier implies that ZnTCPP‐Zn can perform electron transfer and oxygen reduction more efficiently, resulting in faster reaction kinetics. In contrast, the differential charge density and free energy pathways of Zn‐TCPP‐Al and Zn‐TCPP‐Ti indicate lower electron transfer at the central Zn atom. This is likely due to Al and Ti cations exhibiting stronger electron‐sharing or screening effects, which weaken the activity of the central Zn atom in the Zn‐TCPP framework. Additionally, the significant differences in free energy barriers among the three materials throughout the process are conducive to the establishment of a sensor array.

### 2.4 Photoresponsive Nanoenzyme Sensor Arrays for Neurotransmitters Detection

**Figure 4:**
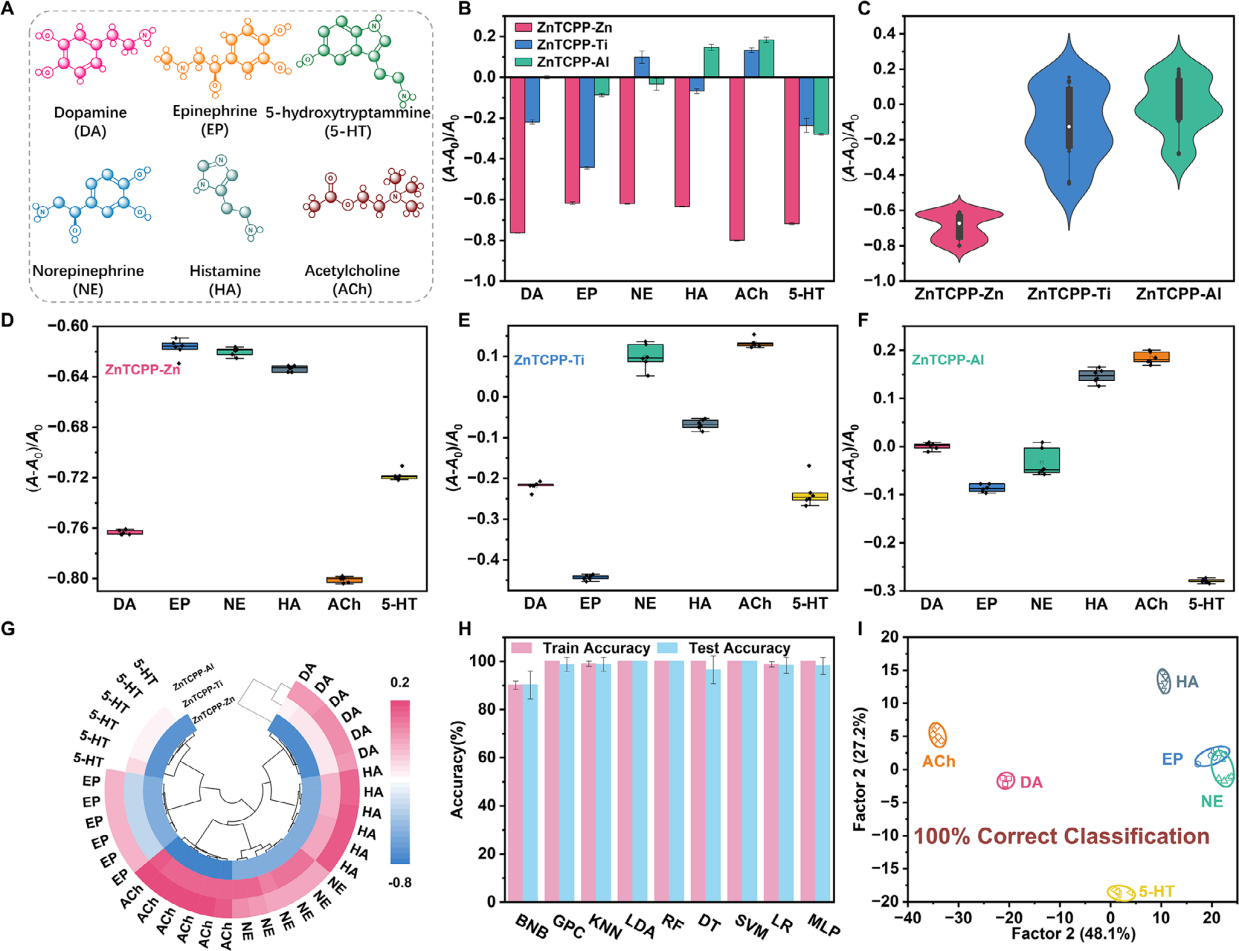
Pattern recognition of neurotransmitters using nanozyme sensor array. A) The scheme of the structure of neurotransmitters. B) Colorimetric response patterns [(*A*‐*A*_0_)/*A*_0_] of the sensor array toward six neurotransmitters. C) Violin plots of the distribution of corimetric response patterns characterization data for neurotransmitters. Distribution boxplots of the colorimetric responses for six neurotransmitters at ZnTCPP‐Zn D), ZnTCPP‐Ti E), and ZnTCPP‐Al F). G) Clustered heat map derived from the changes of the array signal responses for six neurotransmitters. H) Comparison of accuracies for neurotransmitters by employing different machine learning algorithms on the training and prediction. I) LDA canonical score plot using the first two factors obtained from the corimetric response pattern. Each point represents the response pattern for a single neurotransmitter. The ellipse areas represent 95% confidence intervals.

Simultaneous detection of various neurotransmitters remains challenging due to their similar structures and reactivity, complicating their differentiation by a single sensor. The integration of nanozyme sensor array, which leverages specific binding interactions and catalytic activities, generates a cross‐reactive signal, thereby establishing an effective platform for the concurrent detection of multiple neurotransmitters. An oxidase‐like activity sensor array, based on metal node‐modulated ZnTCPP‐M nanozyme, was developed for this purpose. This study explores the modulation effects of neurotransmitters on the oxidase‐like activities of three different ZnTCPP MOFs nanozymes. To verify the feasibility of this sensor array, the colorimetric response variations of the sensing systems after the addition of neurotransmitters were investigated. Six common neurotransmitters, include dopamine (DA), epinephrine (Ep), norepinephrine (NE), serotonin (5‐HT), histamine (HA), acetylcholine (Ach), were analyzed, with their molecular structures presented in **Figure** **4**A. The six neurotransmitters were selected due to their well‐established clinical significance and frequent dysregulation in major neurological and psychiatric disorders, including Alzheimer's disease, Parkinson's disease, schizophrenia, depression, and anxiety. They are routinely assessed in clinical settings and serve as key neurochemical biomarkers for evaluating neuropathological conditions and monitoring therapeutic responses. These neurotransmitters were categorized into three categories: i) catecholamine neurotransmitters including DA, NE, Ep; ii) charged neurotransmitters such as ACh; and iii) monoamine neurotransmitters, like 5‐HT, HA. As shown in Figure 4B, each nanozyme in this array exhibited a diverse colorimetric signal response to the six neurotransmitters. Different neurotransmitters elicited varying effects on the same nanozyme. The colorimetric signal response of ZnTCPP‐Zn nanozyme reduced significantly upon the addition of neurotransmitters, while the colorimetric signal response of ZnTCPP‐Ti and ZnTCPP‐Al were increased or decreased with different degrees. This demonstrated that various neurotransmitters differently affect nanozyme activity (Figures S8 and S9 and Table S2, Supporting Information). This phenomenon can be attributed to the unique molecular structures and chemical properties of different neurotransmitters, which influence their interactions with the metal nodes and porphyrin ligands of the ZnTCPP‐MOFs nanozymes. These interactions can either inhibit or enhance the oxidase‐like catalytic activity of the nanozymes, leading to variations in the oxidation rate of the colorimetric substrate (TMB) and consequently, differences in color intensity. Neurotransmitters with electron‐donating groups may reduce the electron density at the catalytic center, decreasing the oxidation efficiency and resulting in a weaker color response, as observed with ZnTCPP‐Zn. Conversely, neurotransmitters with electron‐withdrawing groups can facilitate electron transfer, enhancing the catalytic activity and producing a stronger colorimetric signal, as seen with ZnTCPP‐Ti and ZnTCPP‐Al. DFT calculations were conducted to investigate the binding modes between ZnTCPP‐Zn and six representative neurotransmitters. The results revealed that ACh exhibits the strong binding affinity, dominated by electrostatic interaction and auxiliary Zn–O coordination. DA and 5‐HT primarily interact through Zn–O coordination combined with π–π or hydrophobic interactions, whereas HA, NE, and EP rely on weaker Zn–N or non‐covalent interactions. These observations highlight the critical role of functional groups (e.g., quaternary ammonium, catechol, indole, imidazole) and their spatial orientation in modulating nanozyme activity and signal output (Figure S10, Supporting Information). This diverse modulation of nanozyme activity by different neurotransmitters is essential for the sensor array's ability to generate unique response patterns and achieve precise discrimination. A violin plot of the raw data was generated (Figure 4C), visually representing the broad variability within the data from the array, and highlighting the significant responsiveness and variability of the colorimetric response. To gain a deeper understanding of the data distribution, a distribution box plot of the original data was generated. The box plot (Figure 4D–F) reveals significant differences in the colorimetric response across various analytes.

In the clustered heat map, each nanozyme within the sensor array demonstrated unique colorimetric changes in response to neurotransmitters (Figure 4G). The colorimetric response patterns of neurotransmitters were transformed into Euclidean distance by a hierarchical clustering algorithm (HCA), leading to the successful classification of all six neurotransmitters with no misclassification, indicating the array's robust ability to distinguish between different types of neurotransmitters. To improve the ability of the sensor array to discriminate six neurotransmitters and predict unknown samples by employed multiple machine learning algorithms for model training, including Bernoulli Naive Bayes (BNB), Gaussian Process Classifier (GPC), K‐nearest Neighbors (KNN), Random Forest (RF), Decision Tree (DT), Support Vector Machine (SVM), Linear Discriminant Analysis (LDA) and Logistic Regression (LR) (Figure 4H). Only the BNB algorithm, which had an accuracy of less than 95%, most algorithms achieved train and test accuracies above 95%. BNB demonstrated high computational efficiency and was well‐suited for high‐dimensional categorical data. However, its assumption of feature independence limited its classification stability due to feature correlations in the dataset. KNN effectively captured the distinct clustering patterns of neurotransmitter responses and performed exceptionally well, although it is sensitive to noise and computationally demanding with larger datasets. LDA exhibited excellent performance, efficiently separating linearly distributed data while maintaining high interpretability and low computational cost. RF provided strong robustness against overfitting by aggregating multiple decision trees, although its interpretability decreased as the ensemble size grew. DT models offered intuitive decision boundaries but were prone to overfitting without proper regularization. SVM effectively handled high‐dimensional sensor data through margin maximization, requiring careful kernel and parameter optimization to achieve optimal results. LR performed well on linearly separable data but was less robust in capturing complex non‐linear patterns. MLP, a neural network model, demonstrated strong capabilities in modeling complex feature interactions but required extensive hyperparameter tuning and greater computational resources. Overall, LDA and SVM offered an optimal balance between accuracy, generalization, and computational efficiency, making them particularly promising for neurotransmitter identification. To comprehensively evaluate the performance of the models, we calculated precision, recall, F1 score, and ROC‐AUC (Figures S11 and S12 and Table S3, Supporting Information). Most algorithms, including KNN, LDA, RF, and SVM, achieved perfect scores across all four metrics (value = 1), indicating exceptional consistency and reliability. In contrast, BNB and DT showed slightly lower values in precision and recall, with corresponding F1 scores of 0.8622±0.1287 and 0.9589±0.0884, and slightly reduced AUCs. These results highlight the robustness and practical applicability of the proposed machine learning‐integrated sensor array strategy for accurate neurotransmitter identification.

A visual discrimination model was implemented using the LDA algorithm. The training matrix (3 ZnTCPP MOFs nanozyme × 6 neurotransmitters × 6 replicates) was converted into typical scores using the LDA algorithm. In the LDA plot, each factor represents the degree of contribution to the overall categorization, showing that Factor 1 and Factor 2 depended on the two contributing factors account for 48.1% and 27.2% of the variance, respectively. The six neurotransmitters were categorized into six different groups. All neurotransmitters were clearly categorized without any misclassification, and the discrimination accuracy was 100% (Figure S13 and Table S4, Supporting Information). 32 unknown neurotransmitters were correctly recognized with 100% accuracy (Table S5, Supporting Information). To further investigate the contribution of individual sensor elements to the classification performance, we conducted a feature importance analysis using both LDA and SHapley Additive exPlanations (SHAP) derived from an RF model. The canonical coefficients obtained from LDA reflect the relative contribution of each MOF‐based sensor to discriminating different neurotransmitters. As shown in Figure S14A (Supporting Information), ZnTCPP‐Zn demonstrated the highest LDA importance, followed by ZnTCPP‐Al and ZnTCPP‐Ti, consistent with their intrinsic catalytic activity and signal intensity. Complementary SHAP analysis supported this trend: ZnTCPP‐Zn exhibited the highest SHAP value, suggesting it plays a dominant role in classification. ZnTCPP‐Al and ZnTCPP‐Ti also contributed meaningfully but to a lesser extent (Figure S14B, Supporting Information). These findings collectively highlight the synergistic effect of the multicomponent array, where ZnTCPP‐Zn offers strong discriminatory power, while ZnTCPP‐Ti and ZnTCPP‐Al increase diversity and orthogonality in pattern recognition. In addition to the six neurotransmitters, the study was expanded to include amino acid neurotransmitters such as γ‐aminobutyric acid (GABA) and glutamate (Glu). Distinct colorimetric responses were observed for all eight neurotransmitters using the nanozyme sensor array. The array demonstrated the capability to detect and discriminate a broader range of neurotransmitters, with model and prediction accuracies reaching 100%. These results not only highlight the multifunctionality and robustness of the sensor array platform but also validate its scalability for broader applications in neurotransmitter detection (Figure S15, Supporting Information).

Furthermore, the detection of lower concentrations of neurotransmitters was investigated, with the sensor array continuing to show distinct responses to various neurotransmitter types even at lower concentrations (0.1 and 1 µm) (Figures S16–S18 and Tables S6 and S7, Supporting Information). Remarkably, the sensor array was able to accurately discriminate between six neurotransmitters at these lower concentrations, with both model accuracy and prediction accuracy from the jackknifed classification of the array reaching 100% (Figures S19 and S20 and Tables S8–S11, Supporting Information). The sensor array demonstrated excellent performance in neurotransmitter detection.

### 2.5 Differentiation of Individual Neurotransmitters in Different Concentrations

**Figure 5:**
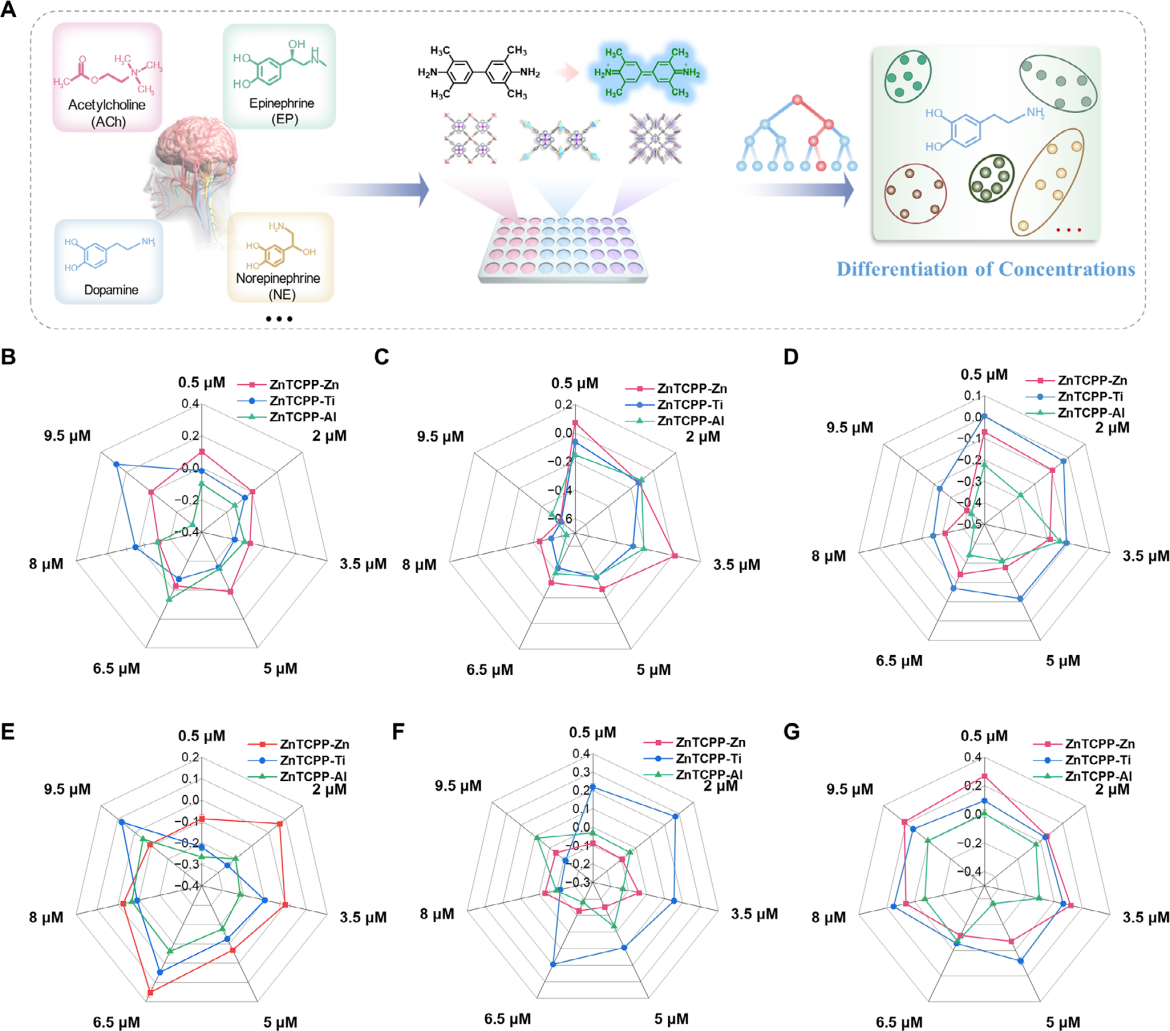
Differentiation of individual neurotransmitters in different concentrations. A) Schematic illustration of the mechanism of neurotransmitter sensing at different concentrations. Radar maps of ratiometric fluorescence response patterns toward different concentrations of B) DA, C) EP, D) 5‐HT, E) NE, F) HA, and G) ACh.

**Figure 6:**
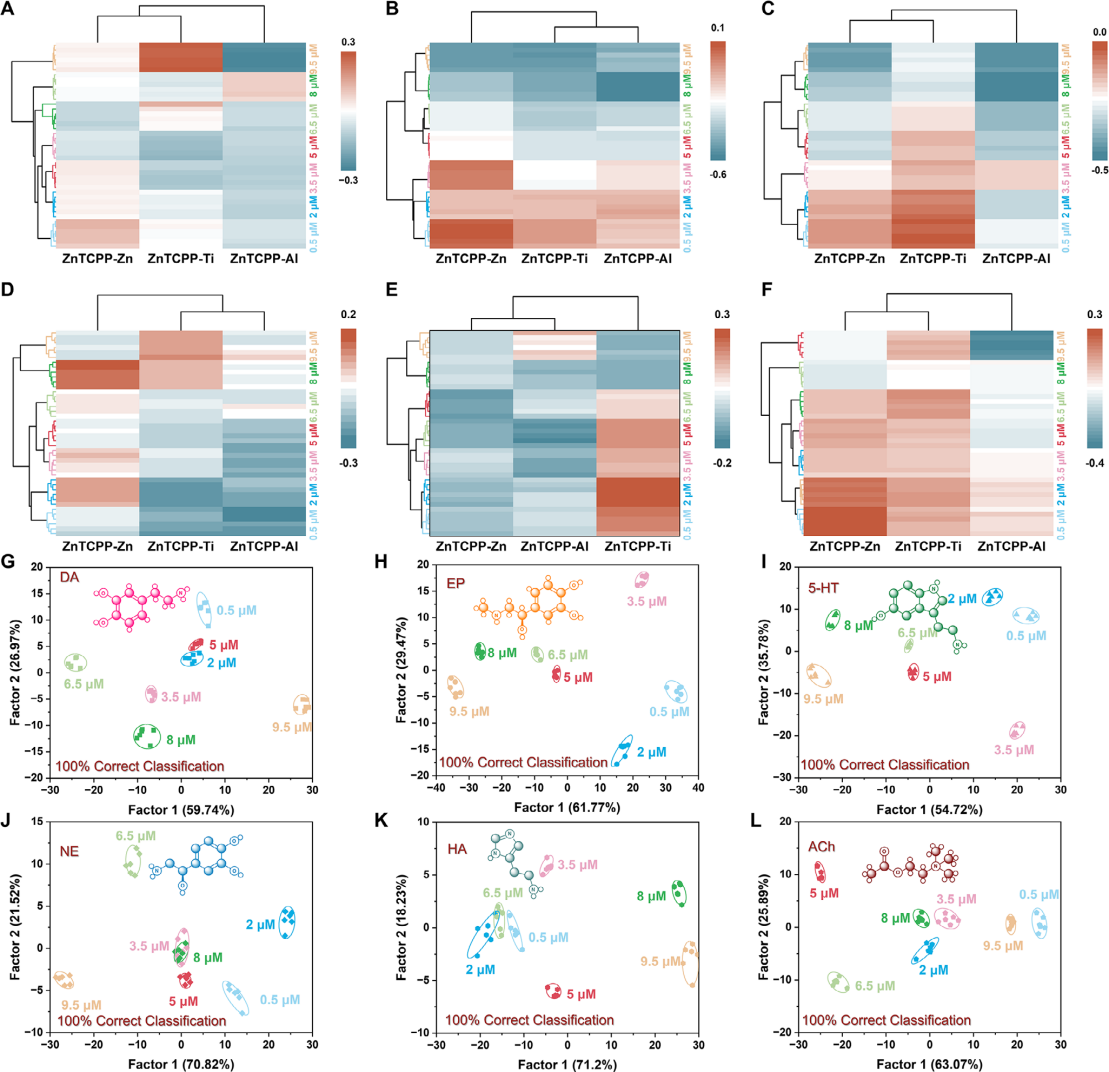
Visualization of six neurotransmitters at different concentrations. Clustered heat map derived from the changes of the array signal responses for A) DA, B) EP, C) 5‐HT, D) NE, E) HA, and F) ACh with different concentrations. LDA canonical score plots of ratiometric sensor array for G) DA, H) EP, I) 5‐HT, J) NE, K) HA and L) ACh with different concentrations.

After successfully identifying different types of neurotransmitters, the sensor array was further evaluated for its ability to differentiate varying concentrations. Six neurotransmitters were selected, covering a range of concentrations from 0.5 to 9.5 µm (**Figure** **5**A). The colorimetric response patterns at different concentrations of each analyte with the sensor array were recorded. As shown in Figure 5B–G, the sensor array exhibited distinct colorimetric responses to individual neurotransmitters at various concentrations. The radius transformation of the radar plot reflected the relative colorimetric response change. The shape and size of the radar maps at seven concentrations of each neurotransmitters were different, indicating that the seven concentrations could be easily distinguished by their diverse three‐signal radius (Tables S12–S17, Supporting Information). In the clustered heatmap, each channel displayed unique colorimetric changes in response to six neurotransmitters at different concentration (**Figure** **6**A–F). The different concentrations of each neurotransmitter were clearly categorized into seven distinct clusters, with the same concentration grouped into one category without misclassified. The data matrix, consisting of 7 concentrations × 3 sensor elenments × 6 replicates, was further analyzed using LDA algorithm. As shown in Figure 6G–L., the seven concentrations of each neurotransmitter were successfully distinguished from one another and were distinctly clustered into separate groups. Although NE and HA slightly overlapped in Factor 1 and Factor 2, they were well discriminated in Factor 1 and Factor 3. The jackknifed classification matrix revealed a discrimination accuracy of 100% (Tables S18–S23 and Figures S21–S26, Supporting Information). The prediction accuracy for the six neurotransmitters at different concentrations was 97%, 100%, 100%, 100%, 96%, and 100%, respectively (Tables S24–S29, Supporting Information). This demonstrated that the array is capable of semi‐quantitative detection of various neurotransmitters.

To further assess the performance of the sensor array, we focused on the differentiation of DA analogue mixtures with different molar ratios (the total analogue concentration was 5 µm). The mixtures investigated consisted of different ratios of DA and EP, as well as DA and NE. For each mixture, the sensor array displayed unique colorimetric response patterns that were clearly distinguishable by LDA (Figures S27–S29 and Tables S30 and S31, Supporting Information). The model successfully categorized the mixtures without any misclassification, achieving a classification accuracy of 100% (Tables S32 and S33, Supporting Information). Additionally, the prediction accuracy for all tested concentrations of the DA analogue mixtures also reached 100%, confirming the robustness and precision of the sensor array in differentiating between the various mixtures (Tables S34 and S35, Supporting Information). This high performance was consistent across different machine learning models, solidifying the sensor array's reliability for real‐time, multiplexed detection of neurotransmitter mixtures.

### 2.6 Identification of Neurotransmitters in Real Samples

**Figure 7:**
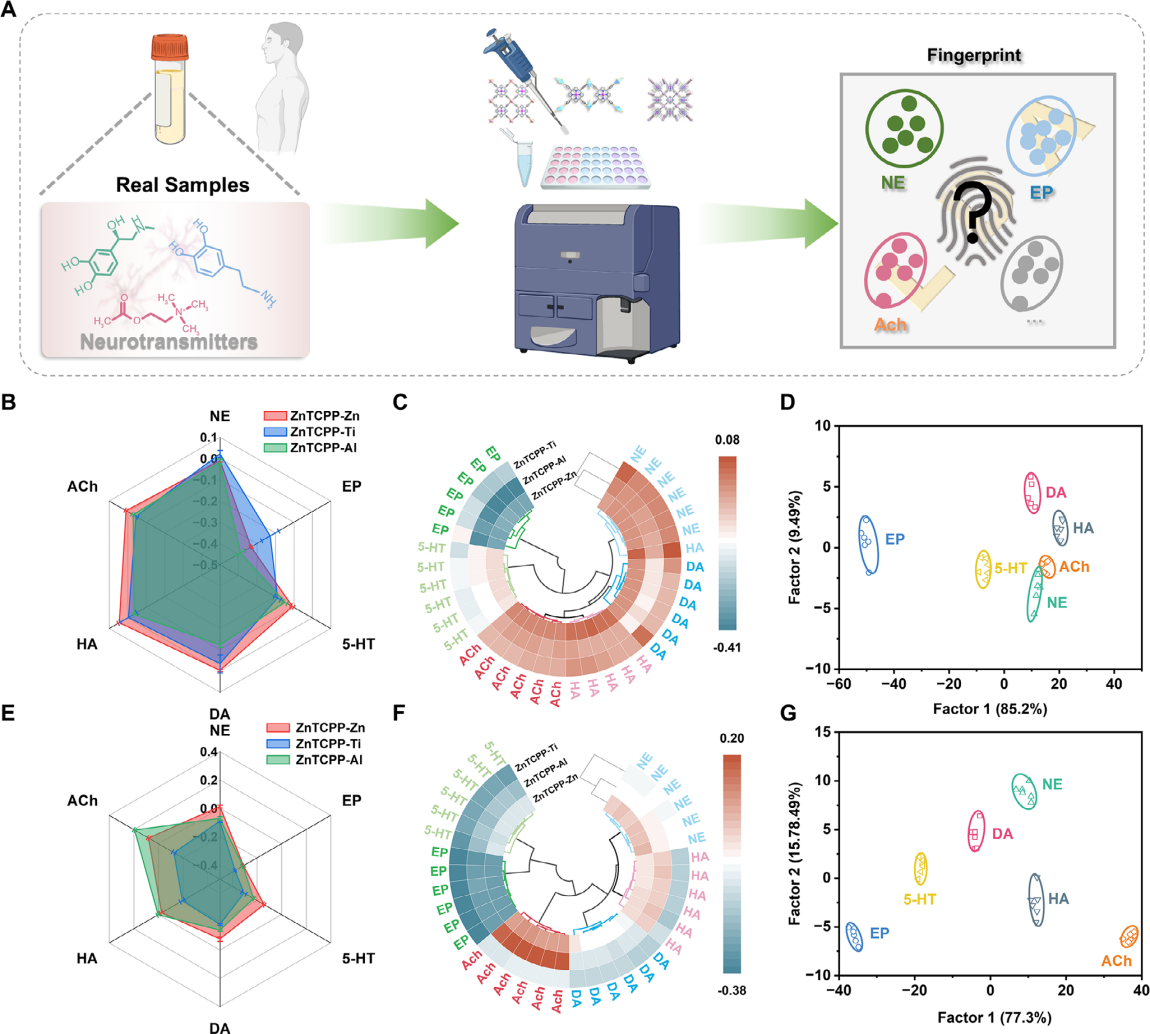
Nanozyme identification of humoral neurotransmitters. A) Schematic diagram of the detection process for neurotransmitters in real samples. Radar maps of colorimetric response patterns toward different kinds of neurotransmitters B) in cerebro‐spinal fluid and E) in serum. Clustered heat map derived from the changes of the array signal responses for different kinds of neurotransmitters C) in cerebro‐spinal fluid and F) in serum. LDA canonical score plots of ratiometric sensor array for different kinds of neurotransmitters D) in cerebro‐spinal fluid and G) in serum.

The sample matrix for neurotransmitters detection primarily includes cerebro‐spinal fluid and serum. The complexity of the composition of the sample matrix, along with the presence of numerous interfering substances, increases the difficulty of analyzing neurotransmitters. Based on the prior experiments, the applicability of the sensor array was further investigated with real samples. Real samples from different sources, spiked with six neurotransmitters each at the concentration of 10 µM, were analyzed using the sensor system (**Figure** **7**A; Figures S30 and S31 and Tables S36 and S37, Supporting Information). The shape and size of each neurotransmitters in the complex matrix, as depicted in radar maps, indicated that the six neurotransmitters can be easily distinguished by their diverse three‐signal radius (Figure 7B,E). In the clustered heatmap, the six neurotransmitters exhibited varying degrees of influence on three enzyme‐like activities in erebro‐spinal fluid and serum, respectively (Figure 7C,F). The neurotransmitters were clearly categorized into six distinct clusters without any misclassification in either cerebrospinal fluid or serum. LDA results showed the six neurotransmitters in different matrix were obviously separated from each other (Figure 7D,G; Figures S32 and S33, Supporting Information). The jackknifed classification matrix with cross‐validation revealed 100% accuracy in real sample (Tables S38 and S39, Supporting Information). Blind tests also demonstrated 100% accuracy (Tables S40 and S41, Supporting Information). These results indicate that the sensor array possesses anti‐interference capability and successfully realizes the detection of neurotransmitters in complex samples. To assess the anti‐interference capability of the array, representative interfering substances were introduced, including human serum albumin (HSA), amyloid β‐protein 40 (Aβ40), γ‐globulins (γ‐glob), cholesterol (Chol), and triolein (TO), each at a concentration of 10 µm. As shown in Figure S34A (Supporting Information), while the addition of interferents induced noticeable changes in the colorimetric signals of the nanozymes, distinct response patterns were observed. Specifically, ZnTCPP‐Zn exhibited an enhanced colorimetric response toward interferents, and ZnTCPP‐Ti and ZnTCPP‐Al also showed enhanced responses to most of them. These trends were substantially different from those triggered by neurotransmitters, enabling effective differentiation based on the direction and magnitude of signal modulation. Subsequent LDA of the dataset (3 sensor elements × (6 neurotransmitters + 5 interfering substances) × 6 replicates) demonstrated clear separation of all analytes with a classification accuracy of 100% (Figure S34B, Supporting Information). Neurotransmitters and interferents were distinctly clustered on the LDA score plot, with neurotransmitters predominantly located on the left and interferents on the right. Moreover, lipid‐type interferents (Chol and TO) and protein‐type interferents (HSA, γ‐glob, and Aβ40) clustered closely within their respective categories, confirming the array's excellent discrimination ability. The presence of these common biological interferents had minimal impact on the identification accuracy for neurotransmitters, highlighting the strong anti‐interference robustness of the array. The nanozyme sensor array demonstrated comparable sensitivity to conventional methods while offering faster detection, higher specificity, and simpler operation without requiring complex sample preparation or highly trained personnel. Moreover, the array is cost‐effective due to the facile synthesis and stability of MOF‐based nanozymes. A detailed comparison between the method described in this study and various conventional detection methods is summarized in Table S42 (Supporting Information). These advantages make it a promising tool for neurotransmitter analysis in clinical applications.

### 2.7 Machine Learning for Diagnosis of Neurological Diseases

**Figure 8:**
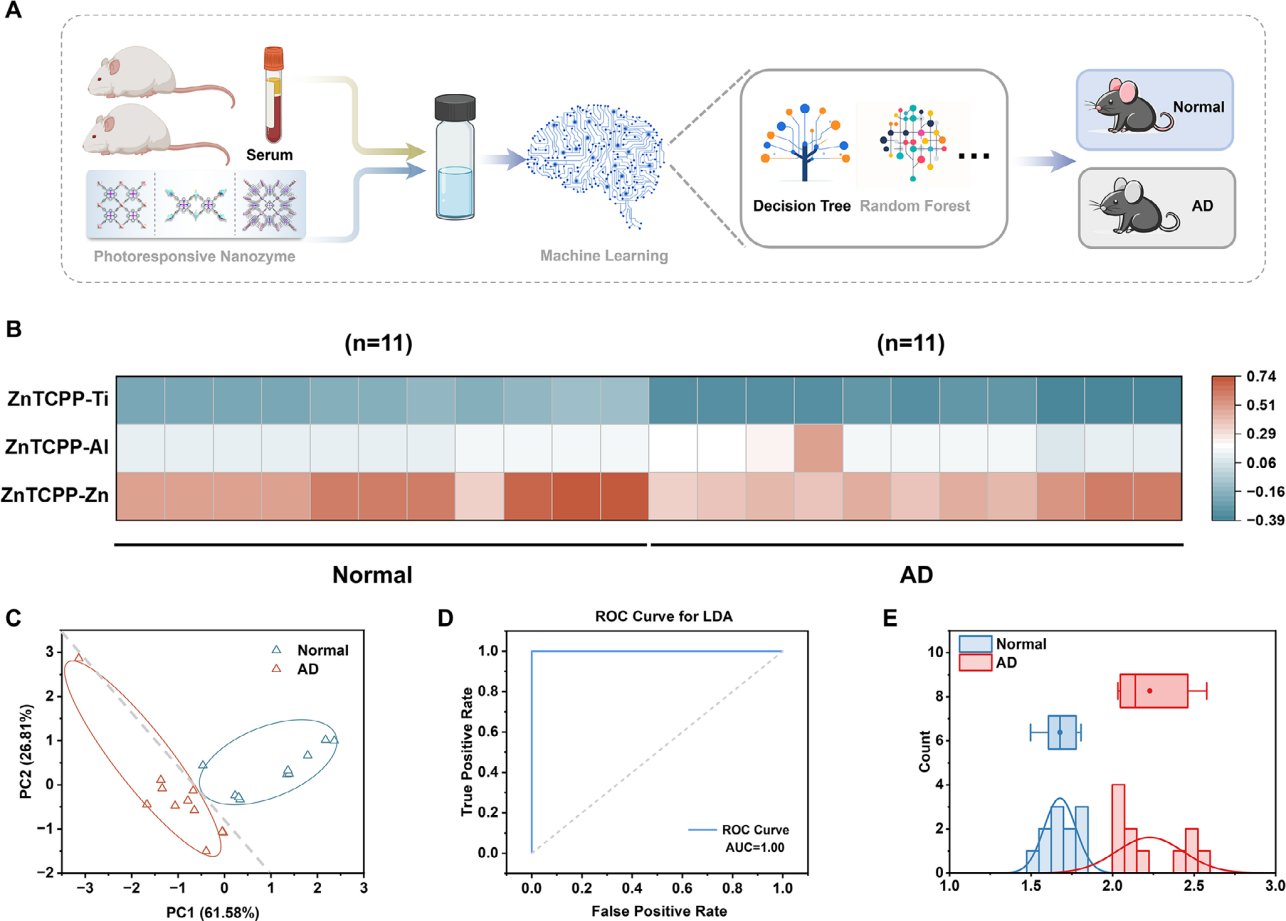
Machine learning for diagnosis of AD. A) Schematic illustration of detection in normal and AD Mice. B) Heatmap derived from the changes of the array signal responses for normal and AD samples (11 normal samples and 11 AD sample). C) PCA score plots for the identification of AD sample and normal sample individuals. D) ROC curves for LDA in identifying disease samples. E) Distribution of differentiation results for the actual sample.

Alzheimer's disease (AD) is a neurodegenerative disorder that significantly impacts public health, making early detection critical for improving patient outcomes. To demonstrate the real‐world application, we employed a sensor array combined with machine learning to distinguish serum from AD model mice using healthy mice as a control group for clinical diagnosis (**Figure** **8**A). We obtained serum samples from 22 mice and generated a dataset (3 sensor elements × 22 samples) for AD diagnosis (Table S43, Supporting Information). A heatmap generated from the sensor array responses clearly demonstrated distinct changes in the signal patterns between the two groups, illustrating the sensor's capability to detect subtle differences indicative of AD (Figure 8B). We applied principal component analysis (PCA) to generate independent principal components. PCA score plots effectively distinguish between normal and AD samples, with the first two principal components explaining a significant portion of the variance, reinforcing the sensor array's discriminatory power (Figure 8C). To evaluate the performance of this sensor array in diagnosing AD, we employed several machine learning algorithms. For a more rigorous assessment of model performance and generalizability, we adopted a stratified random train‐test split strategy that preserved class distributions across all subsets. Multiple test set ratios were systematically evaluated to identify the most stable and reliable partitioning scheme. As shown in Figure S35 (Supporting Information), a 60/40 split achieved the highest and most consistent classification accuracy across different algorithms. This ratio was therefore selected for all subsequent analyses. The comparison of training and prediction accuracies revealed that LDA and DT achieved the highest performance, with 100% accuracy in distinguishing normal from AD samples (Figure S36, Supporting Information). To further evaluate the predictive performance of each model, confusion matrices based on test set predictions were generated and are provided (Figure S37, Supporting Information). Both LDA and DT offered an optimal combination of accuracy, interpretability, and generalization ability. Models such as RF, SVM, and MLP effectively captured complex feature interactions but their performance was slightly affected by noise and class overlaps. Simpler models like BNB, KNN, and LR demonstrated good efficiency but exhibited limitations when handling feature correlations or non‐linear patterns. These performance differences were further reflected in the quantitative classification metrics. As shown in Figures S38 and S39 (Supporting Information), LDA and DT achieved perfect scores across all evaluation metrics (1.0000), indicating outstanding discriminative power. BNB and RF also demonstrated strong performance, with F1 scores of 0.9775 ± 0.0756 and 0.9232 ± 0.0959, and corresponding AUCs of 0.9990 ± 0.0070 and 0.9895 ± 0.0258, respectively. In contrast, KNN exhibited slightly lower stability, with an F1 score of 0.886 ± 0.1331 and a precision of 0.9182 ± 0.0867 (Table S44, Supporting Information). LDA showed the largest AUC, indicating superior sensitivity and specificity (Figure 8D). These results highlight the effectiveness of combining sensor array technology with machine learning for diagnosing AD. Additionally, we explored the distribution of differentiation results for actual samples, which further validated the high accuracy and robustness of the model in practical applications (Figure 8E). To support algorithm selection in this diagnostic context, we performed a comprehensive comparison across multiple commonly used classifiers. While several models demonstrated competitive accuracy, Linear LDA provided the most balanced combination of performance, simplicity, and interpretability. LDA achieved 100% classification accuracy in both jackknife and independent test‐set validations, and maintained excellent stability across repeated stratified train‐test splits. Importantly, the assumptions underlying LDA were consistent with the characteristics of our dataset, which comprised standardized sensor responses, balanced binary labels, and moderate feature dimensionality. These findings strongly support the selection of LDA as the most appropriate and practical classifier for use in diagnosing Alzheimer's disease in mouse models.

To assess the clinical applicability of the developed sensor array, we also collected clinical serum samples from individuals diagnosed with neurological disorders, including AD (*n* = 10) and PD (*n* = 9), as well as healthy controls (*n* = 20), totaling 39 participants. The colorimetric responses of each nanozyme sensor in the array toward these serum samples were systematically evaluated. As illustrated in Figure S40A (Supporting Information), the sensor array exhibited distinct response patterns for the three groups (Table S45, Supporting Information). Box plots of the raw absorbance data further reveal notable variations in sensor responses, with all nanozymes showing significantly elevated signals in PD samples compared to healthy controls (Figure S40B, Supporting Information). To quantitatively assess the array's diagnostic performance, various machine learning algorithms were applied to classify the clinical samples. As shown in Figure S40C (Supporting Information), models such as RF, LR, and GPC demonstrated strong classification performance. Among them, the RF model yielded the best overall results, achieving an accuracy greater than 98%, an F1 score of 0.962, a recall of 0.958, and the lowest standard deviation, demonstrating excellent stability and generalization capability (Figure S40D, Supporting Information). The ROC curve further confirms the outstanding performance of RF, with an AUC value of 0.996, close to 1, underscoring the potential of the array for the clinical diagnosis of neurodegenerative diseases (Figure S40E, Supporting Information). The sensor array, along with machine learning algorithms, offers a reliable and efficient method for early detection, crucial for improving patient care and therapeutic outcomes in AD. This approach not only demonstrates the potential of sensor arrays in neurological diagnostics but also opens new avenues for real‐time, non‐invasive monitoring of diseases like Alzheimer's.

## 3 Conclusion

In conclusion, the ZnTCPP MOF‐ based photoresponsive nanozyme sensor array offers a powerful detection tool for the rapid detection of neurotransmitters. The integration of ZnTCPP MOFs enhances the sensor's sensitivity, while the light‐driven catalytic properties provide fast and accurate results. Coupled with machine learning, the array enables multiplexed detection, allowing for the differentiation and quantification of neurotransmitter levels in complex samples such as serum and cerebrospinal fluid. Additionally, the practical applications of this sensor array were validated by discriminating the normal and AD samples. This technology represents a significant advancement in diagnostic capabilities, offering a quicker, cost‐effective, and more sensitive alternative to traditional methods. More importantly, the combination of sensor array technology and the photoresponsive nanozyme provides a preeminent method for the simultaneous detection of lowabundance biomolecules, especially for large‐scale screening of diseases.

## Conflict of Interest

The authors declare no conflict of interest.

## Author Contributions

K.Y., S.L., and K.Q. contributed equally to this work. All authors have given approval to the final version of the manuscript.

## Supporting information



Supporting Information

## Data Availability

The data that support the findings of this study are available from the corresponding author upon reasonable request.
