# Supplementary material for: Biomimetic Analysis of Neurotransmitters for Disease Diagnosis through Light‐Driven Nanozyme Sensor Array and Machine Learning
Source: Adv Sci (Weinh). 2025 Jul 6;12(34):e05333. doi: 10.1002/advs.202505333 (PMC12442603; doi:10.1002/advs.202505333)
Supplement: Supplementary file 1 — Supporting Information [file ADVS-12-e05333-s001.docx]

Supporting Information

Biomimetic Analysis of Neurotransmitters for Disease Diagnosis through Light-Driven Nanozyme Sensor Array and Machine Learning

Kun Yu^†b^, Siyuan Lu^†d^, Kaiwen Qiu^†d^, Yuanzun Zhang^d^, Aobing Sun^d^, Shiqi Gong^d^, Kai Wang^e^, Xuzhu Gao*^b^, Xiangyu Xu*^c^, and Hao Wang*^a^

^a^ College of Medical Engineering, Jining Medical University, Jining, 272067, P. R. China

^b^ Lianyungang Clinical College, Jiangsu University & The Second People's Hospital of Lianyungang, Lianyungang, 222006, P. R. China

^c^ College of Basic Medicine, Jining Medical University, Jining 272067, P. R. China

^d^ College of Clinical Medicine, Jining Medical University, Jining 272067, P. R. China

^e^ School of Pharmaceutical Sciences & Institute of Materia Medica, Science and Technology Innovation Center, Shandong First Medical University & Shandong Academy of Medical Sciences, Jinan, 250062, P. R. China

*Corresponding author

E-mail address: [alexgwan@163.com](mailto:alexgwan@163.com); xuxiangyu1212@163.com; wanghao20220819@mail.jnmc.edu.cn

Content List

[MATERIALS AND METHODS 2](#_Toc193486732)

[Supporting Figures 8](#_Toc193486733)

[Supporting Tables 12](#_Toc193486734)

[References 35](#_Toc193486735)

# MATERIALS AND METHODS

**Chemicals and reagents:** Methyl *p*-formylbenzoate, propionic acid, pyrrole, Zincnitrate hexahydrate (Zn(NO_3_)_2_⋅6H_2_O), anhydrous magnesium sulfate, AlCl_3_·6H_2_O, 4-aminobenzoic acid, isopropanol, titanium(IV) isopropoxide, tetrahydrofuran (THF), potassium hydroxide (KOH), 3,3′,5,5′-tetramethylbenzidine (TMB), sodium acetate trihydrate (NaAc), acetic acid (HAc), absolute ethanol (EtOH), N,N-dimethylformamide (DMF), ethyl acetate (EtOAc), L-norepinephrine (NE), histamine (HA), L-epinephrine (Ep), acetylcholine chloride (ACh), serum amine hydrochloride (5-HT), L-isoleucine (ILE), dopamine hydrochloride (DA) were purchased from Shanghai Aladdin Bio-Chem Technology Co., Ltd. Serum and artificial cerebrospinal fluid purchased from Sigma and Source Leaf, respectively. The rats were purchased from Sibefo (Beijing) Biotechnology Co., Ltd, license number SCXK (Beijing) 2022-0009. The serum samples from patients and healthy donors were collected from The Second People's Hospital of Lianyungang (Ethic 2025K061).

**Apparatus:** Transmission electron microscopy (TEM) images were collected from Hitachi HT7700 microscope operating at 120 kV. X-ray photoelectron spectroscopy (XPS) was carried out on Thermo Fisher ESCALAB 250xi spectrometer. Fourier infrared spectrometer (FT–IR, Thermo, USA) was employed to characterize the functional groups on the obtained materials. X−ray diffraction (XRD, Smartlab SE, Japan) data were collected in the 2*θ* range of 5−35^o^ at a step size of 0.01^o^. Microplate reader (BioTek Instruments, USA) was employed to collect absorbance and test kinetics. The ^1^H NMR were acquired using the Bruker Avance (800 MHz). The microplates, consisting of 96 wells, were manufactured by Costar (3590, USA). CEL-PE300L-3A Xenon lamp as a light source for photocatalysis (PerkinElmer300W, China).

**Computational Details:** We used the DFT as implemented in the Vienna Ab initio simulation package (VASP) in all calculations. The exchange-correlation potential is described by using the generalized gradient approximation of Perdew-Burke-Ernzerhof (GGA-PBE). The projector augmented-wave (PAW) method is employed to treat interactions between ion cores and valence electrons. The plane-wave cutoff energy was fixed to 500 eV. Given structural models were relaxed until the Hellmann–Feynman forces smaller than -0.02 eV/Å and the change in energy smaller than 10^-6^ eV was attained. The vacuum thickness was set to be 15 Å to minimize interlayer interactions. During the relaxation, the Brillouin zone was represented by a Γ centered k-point grid of 7×7×1. Grimme’s DFT-D3 methodology was used to describe the dispersion interactions among all the atoms in adsorption models.

**Preparation of metalloporphyrin ZnTCPP**

**
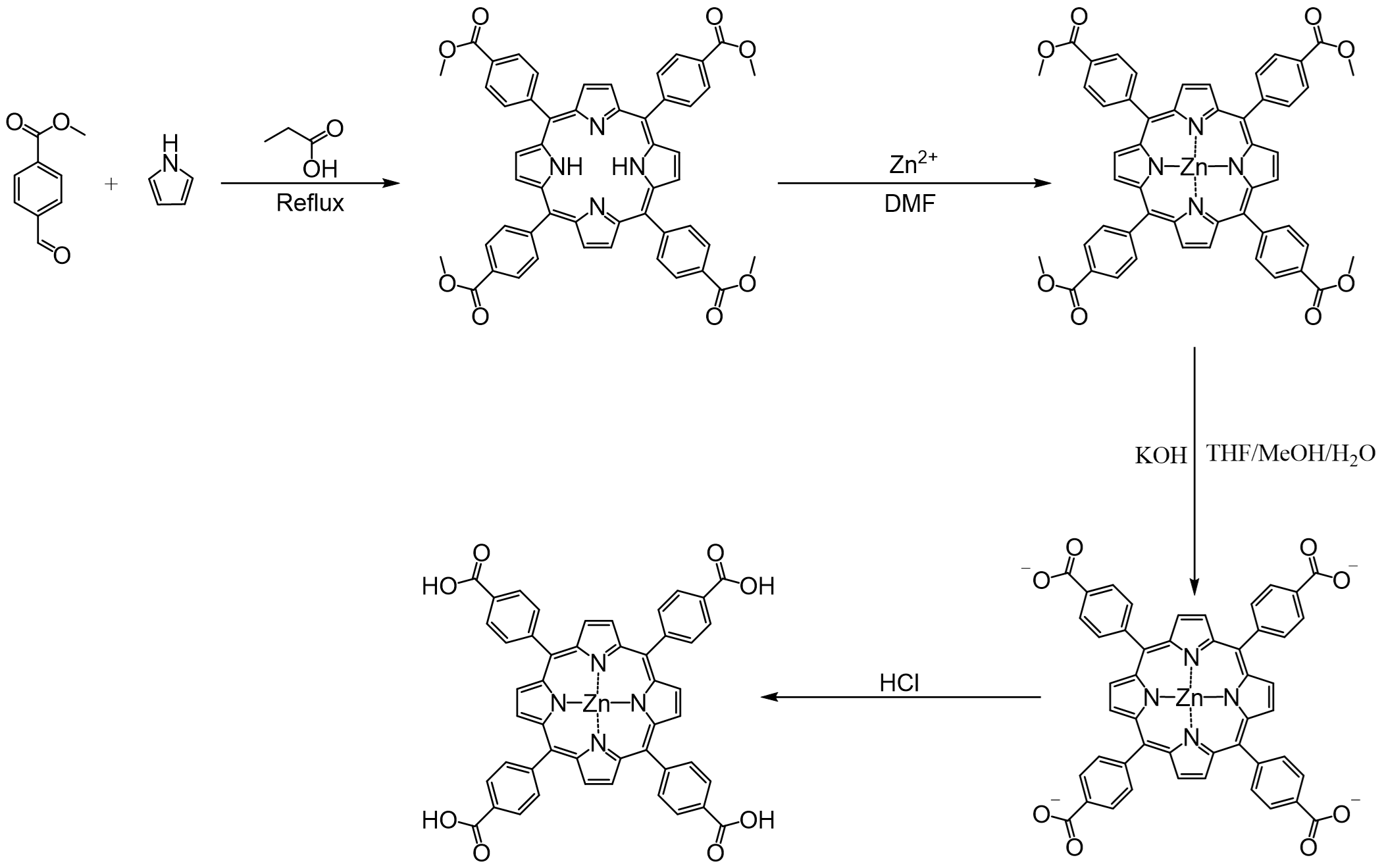
**

**Scheme S1.** Synthesis strategy for ZnTCPP ligand.

The synthesis of ZnTCPP was optimized based on prior literature.[1] First, 6.9 g of methyl *p*-formylbenzoate was fully dissolved in 100 mL of propionic acid at 60 °C. Subsequently, 3 mL of pyrrole was slowly added to the solution. The mixture was then refluxed for 24 hours. After cooling the reaction mixture to room temperature, the product was collected by vacuum filtration to yield purple crystals. Next, 0.5 g of the purple product was refluxed with 2 g of Zn(NO_3_)_2_⋅6H_2_O in 100 mL of DMF for 6 hours. After cooling the mixture to room temperature, 200 mL of water was added. The precipitate was collected by centrifugation and washed twice with 100 mL of water. The obtained solid was dissolved in EtOAc, and the solution was washed three times with water. The organic layer was dried over anhydrous magnesium sulfate and evaporated to obtain a purple solid. Then, 0.7 g of the purple solid was dissolved in 25 mL of THF and 25 mL of MeOH, and a solution of KOH (2.5 g in 20 mL of water) was added. The mixture was refluxed overnight. After the reaction, the mixture was cooled to room temperature, and THF and MeOH were removed under reduced pressure. Additional water and EtOAc were added to the aqueous phase. The solution was then acidified with 1M HCl to transfer the porphyrin to the organic phase, which was subsequently washed three times with water. The organic phase was dried over anhydrous magnesium sulfate and evaporated to yield the purple solid ZnTCPP. FT-IR (KBr): *v* = 1681(s), 1606 (s), 1562 (m), 1500 (m), 1403 (s), 1314 (m), 1178 (m), 993 (s), 865 (m), 793 (s), 764 (m), 719 (m) cm^-1^. ^1^H NMR (400 MHz, DMSO-*d*_6_, ppm) *δ* 8.81 (s, 8H), 8.38-8.33 (m, 16H).


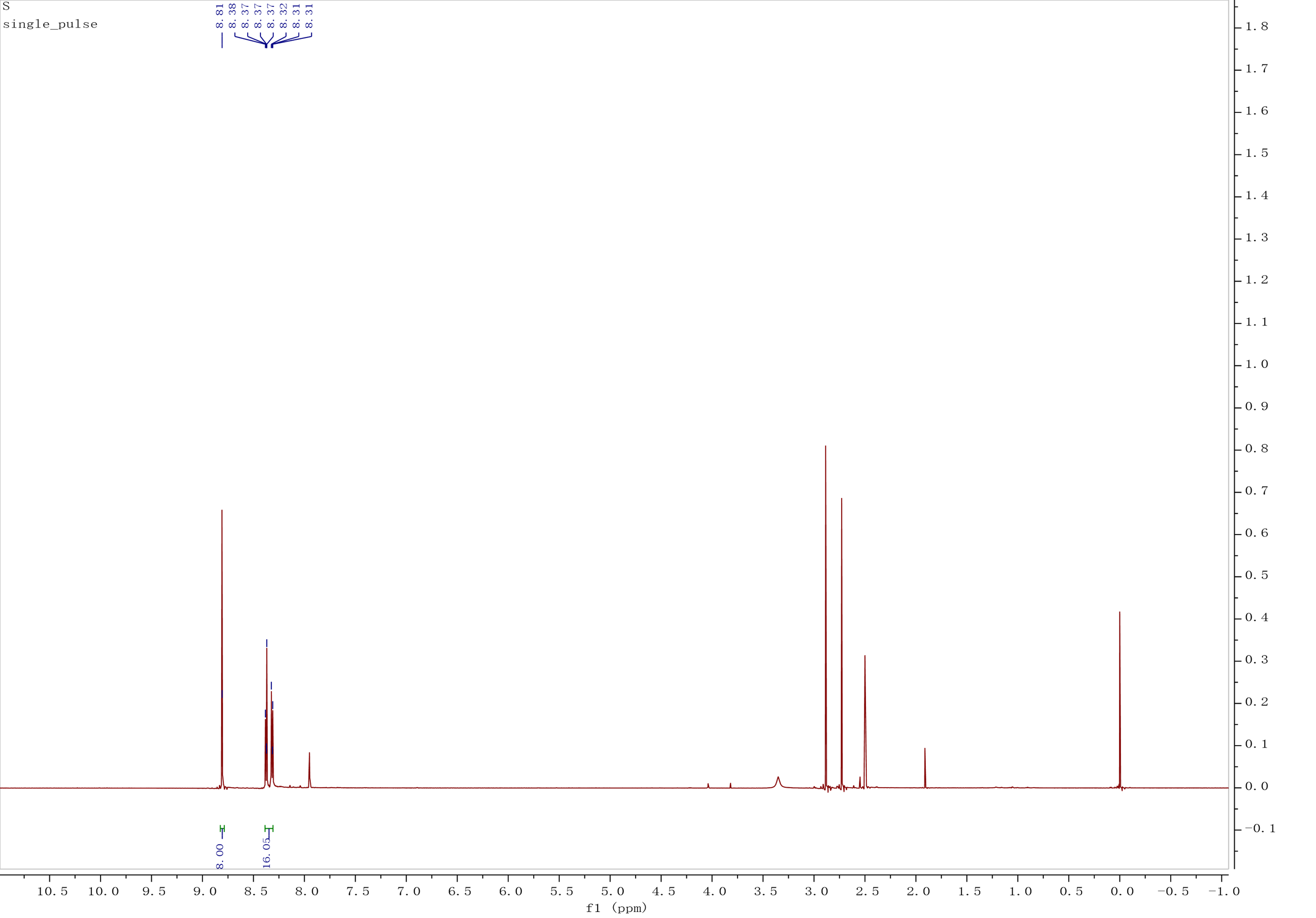


^1^H NMR spectrum of ZnTCPP

**Preparation of metalloporphyrin MOFs: ZnTCPP-Zn, ZnTCPP-Ti, and ZnTCPP-Al:** ZnTCPP-Zn was prepared by a solvothermal method. 2.0 mg of ZnTCPP, 10 mg BA and 5 mg of Zn(NO_3_)_2_⋅6H_2_O (dispersed in 2 mL water) were dissolved in 12 mL of DMF by ultrasound in a 25 mL Schlenk tube, heated and stirred at 150 ℃ for 3 h. Then, the mixture was cooled to room temperature, and ZnTCPP-Zn was obtained by centrifugation and washing five times with ethanol.

ZnTCPP-Al was prepared through a one-step solvothermal method according to the previous report with a little modifications.[2] 10 mg of ZnTCPP and 10 mg of AlCl_3_·6H_2_O were added to 4 mL of deionized water and stirred for 30 minutes to ensure complete dissolution. The suspension was then transferred to a 10-mL Teflon-lined autoclave and heated at 180°C for 16 hours. At the end of heating, the solution was slowly cooled at a rate of 1.5 °C per minute. The solid product was recovered by centrifugation and washed three times with 10 mL of DMF and ethanol to remove unreacted porphyrin and AlCl_3_·6H_2_O. After drying, the ZnTCPP-Al was obtained.

ZnTCPP-Ti was synthesized using a solvothermal method.[3] A mixture of 192 mg of 4-aminobenzoic acid, 5.0 mL of isopropanol, and 104 μL titanium(IV) isopropoxide was introduced into a Teflon-lined autoclave. The mixture was stirred for 30 minutes and then heated at 100°C for 78 hours in an oven. The resulting solid was thoroughly washed with isopropanol to remove any unreacted starting materials. The yellow crystals obtained were subsequently dried at 60°C for 12 hours. Then, 4 mg of yellow crystals , 20 mg of ZnTCPP ligands, and 1 mL of acetic acid were dissolved in 2 mL of a mixed solvent (acetonitrile/tetrahydrofuran = 3 : 1). The resulting solution was stirred in a Teflon autoclave for 15 minutes and then heated at 160°C for 48 hours. The resulting solid was washed with acetonitrile and DMF to remove unreacted ligands and metal clusters, respectively. The dark purple rod-shaped crystals were finally dried at 60 °C for 12 hours.

**Study on the effect of nanozymes on TMB:** To determine the effect of nanozymes on TMB under light conditions, we prepared three sets of experiments mixed with ZnTCPP-M MOF (50 μL), TMB (3.84 mM, 200 μL), and HAc-NaAc buffer solution (650 μL, 0.1 M) (pH = 4.0). The control group repeats the above steps, and the blank group replaces the above medium TMB (50 μL) with DMF (50 μL) and repeats the above steps. The light group and the blank group were irradiated with a 30W flashlight for five minutes to observe the color change of the samples in each group.

Using a UV-Vis spectrophotometer, the scanning spectra of each group of samples in the range of 500-750 nm were determined with DMF (50 μL), TMB (3.84 mM, 200 μL), HAc-NaAc buffer solution (650 μL, 0.1 M) (pH=4.0), and the absorbance of each group of samples at 652 nm was determined simultaneously.

**Kinetic assay of nanozymes:** In order to determine the oxidation of different concentrations of TMB by nanozymes, we prepared different concentrations of TMB with the following steps: 200 μL of different concentrations of TMB, 50 μL of ZnTCPP-M MOF and 650 μL of HAc-NaAc buffer solution (0.1 M, pH = 4.0) were thoroughly mixed to maintain a total volume of 900 μL. After 5 min of light, 200 μL of the solution to be detected was taken in a 96-well plate, and the absorbance of each group at 652 nm was measured in a microplate reader, and each group was measured in parallel for 3 times.

**Detection of different types of neurotransmitters:** Three diverse nanozymes with oxidase-like activities were selected as sensor elements to identify six kinds of neurotransmitters (DA, EP, 5-HT, NE, HA, ACh). In a 96-well plate, 10 μL of MOF, 40 μL of TMB (3.84 mM), 130 μL of HAc-NaAc buffer solution (0.1 mM, pH = 4.0) and 20 μL of neurotransmitter solution were added. The terminal concentration of neurotransmitters was 10 μM. Next, the samples were exposed to light for 5 minutes at room temperature. The absorbance at 652 nm was measured, and each sample was repeated for 6 times. The change in absorbance was expressed as (*A*−*A*_0_)/A_0_. *A* and *A*_0_ represent the absorbance with and without the addition of neurotransmitters, respectively. The identification process for low concentration samples (0.1 μM and 1 μM) are the same as the procedure described above.

**Detection of different concentrations of neurotransmitters:** Six neurotransmitters were diluted to different concentrations with deionized water, respectively. The final concentrations of neurotransmitters were 0.5 μM, 2 μM, 3.5 μM, 5 μM, 6.5 μM, 8 μM and 9.5 μM. Next, repeat the operations described in above. 20 μL of neurotransmitters were mixed with sensor elements in a 96-well plate (6 replicates). The samples were exposed to light for 5 minutes at room temperature. The instrument setup parameters are the same as above.

**Detection of the DA analogue mixture:** To evaluate the ability of the array to differentiate neurotransmitter mixtures, we investigated two binary solutions: DA and NE, as well as DA and Ep. Taking DA and NE as an example, the mixed solutions were prepared with five different ratios. The final concentration ratios of the two neurotransmitters in the mixed solution were DA: NE=5μM: 0μM, 3μM: 1.5μM, 2.5μM: 2.5μM, 1.5μM: 3.5μM, and 0μM: 5μM, respectively. 20 μL of neurotransmitter mixtures with different ratios were mixed with sensor elements in a 96-well plate (6 replicates). The samples were exposed to light for 5 minutes at room temperature. The instrument setup parameters are the same as above. The identification process for the mixed solutions of different ratios of DA and Ep is the same as the procedure described above.

**Different types of neurotransmitters are detected in real samples:** Different types of neurotransmitters were added to real samples (cerebrospinal fluid and serum). The identification process for these samples followed the same procedure as outlined above. The change in absorbance at 652 nm was assessed using the same instrument settings.

**Machine learning for diagnosis of neurological Diseases:** Serum samples were collected from 11 Alzheimer’s disease (AD) model mice and 11 normal mice. The sera were categorized into the disease group and normal group, and each serum was diluted uniformly to prepare the serum dilution solution. For the disease group, 20 μL of the disease group serum dilution were mixed with sensor elements in a 96-well plate. The samples were then exposed to light for 5 minutes at room temperature. Instrument setup parameters were consistent with those described previously. In the normal group, the disease group serum dilution was replaced with the normal group serum dilution, while all other procedures remained unchanged from those of the disease group.

To ensure the robustness of classification under small sample conditions, we employed a set of nine machine learning algorithms and evaluated them over 100 independently stratified train-test splits, recording both training and test performance. Performance metrics including accuracy, F1 score, precision, recall, and ROC-AUC were computed for each classifier. Confusion matrices were used to visualize class-specific predictions. Moreover, we examined different test set ratios to determine the optimal train-test split and enhance evaluation stability .

**Data processing analysis:** Hierarchical cluster analysis, Heatmap, and Radar map were used to analyze the collected data by using Origin 2024b. Machine learning algorithms, including bernoulli naive bayes (BNB), gaussian process classifier (GPC), K-nearest neighbors (KNN), random forest (RF), decision tree (DT), support vector machine (SVM), logistic regression (LR) were built in Python using the scikit-learn package, which is an open-source tool for data analysis and machine learning (https://github.com/scikit-learn/scikit-learn).

Linear discriminant analysis was carried out using classical linear discriminant analysis (LDA) in SYSTAT (version 13.0). In LDA, all variables were used in the model (complete model) and the tolerance was set as 0.001. The fluorescence response patterns were transformed into canonical patterns. The Mahalanobis distances of each individual pattern to the centroid of each group in a multidimensional space were calculated and the assignment of the case was based on the shortest Mahalanobis distance.

# Supporting Figures


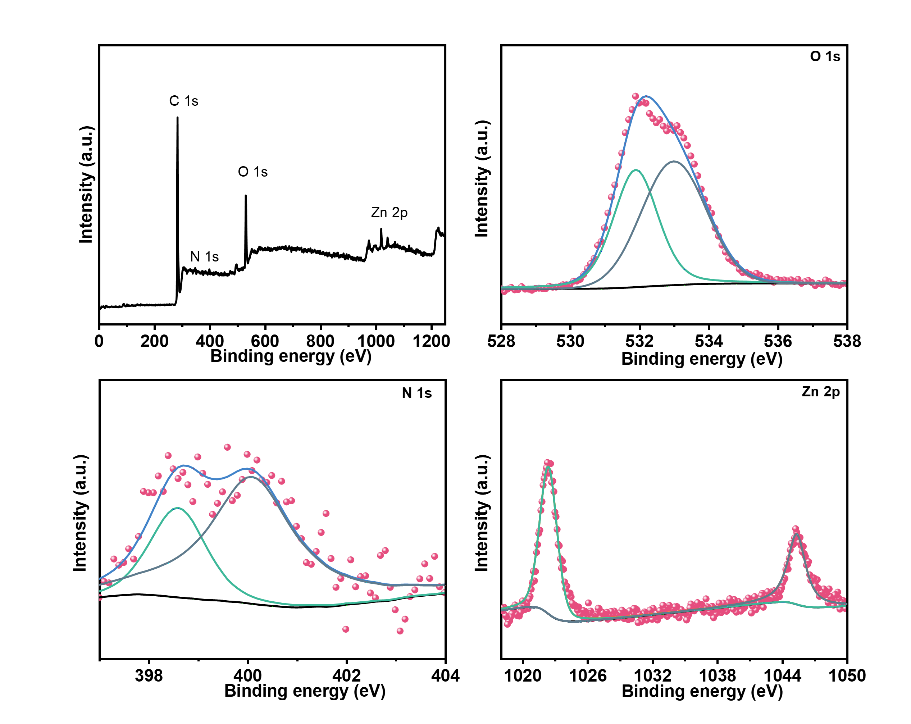


**Figure S1.** Full XPS, O 1s, N 1s, Zn 2p of ZnTCPP-Zn.


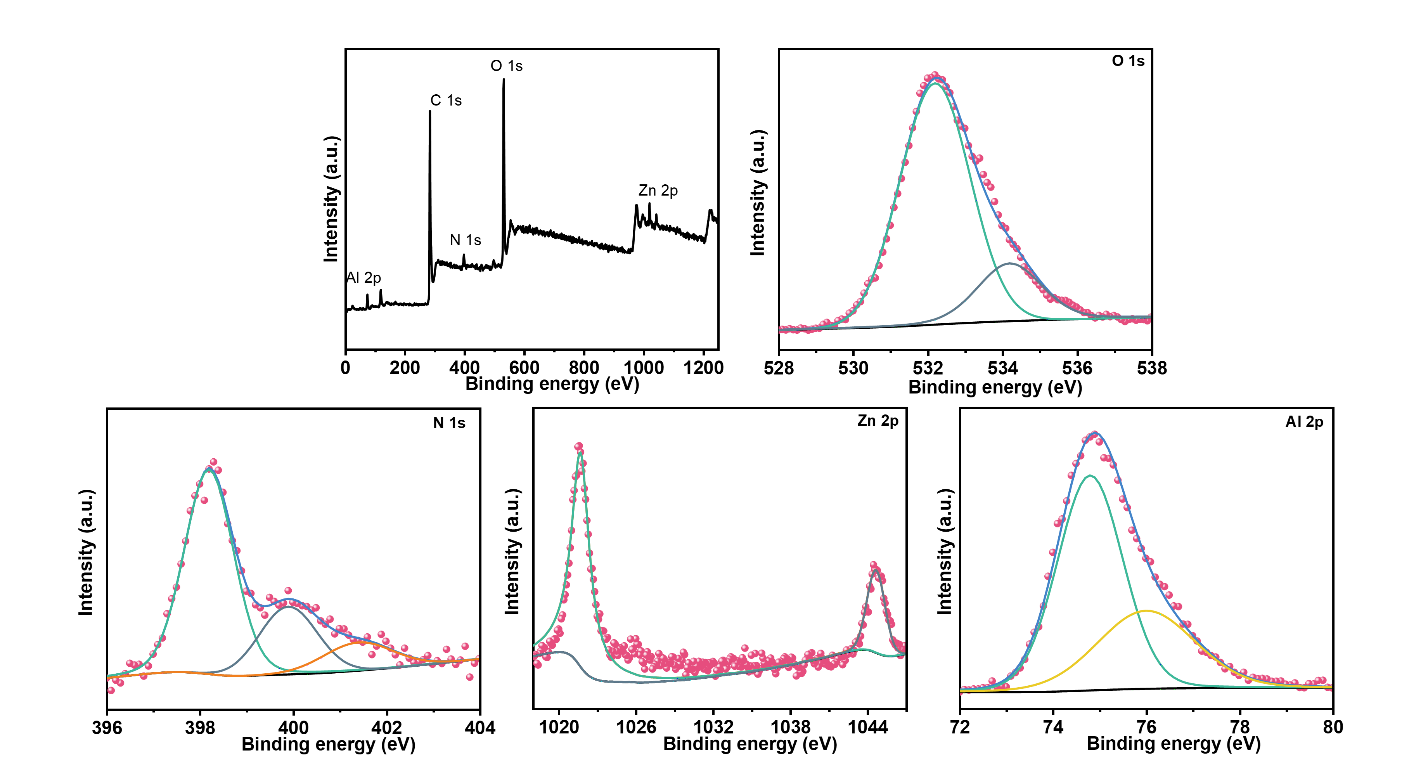


**Figure S2.** Full XPS, O 1s, N 1s, Zn 2p and Al 2p of ZnTCPP-Al.


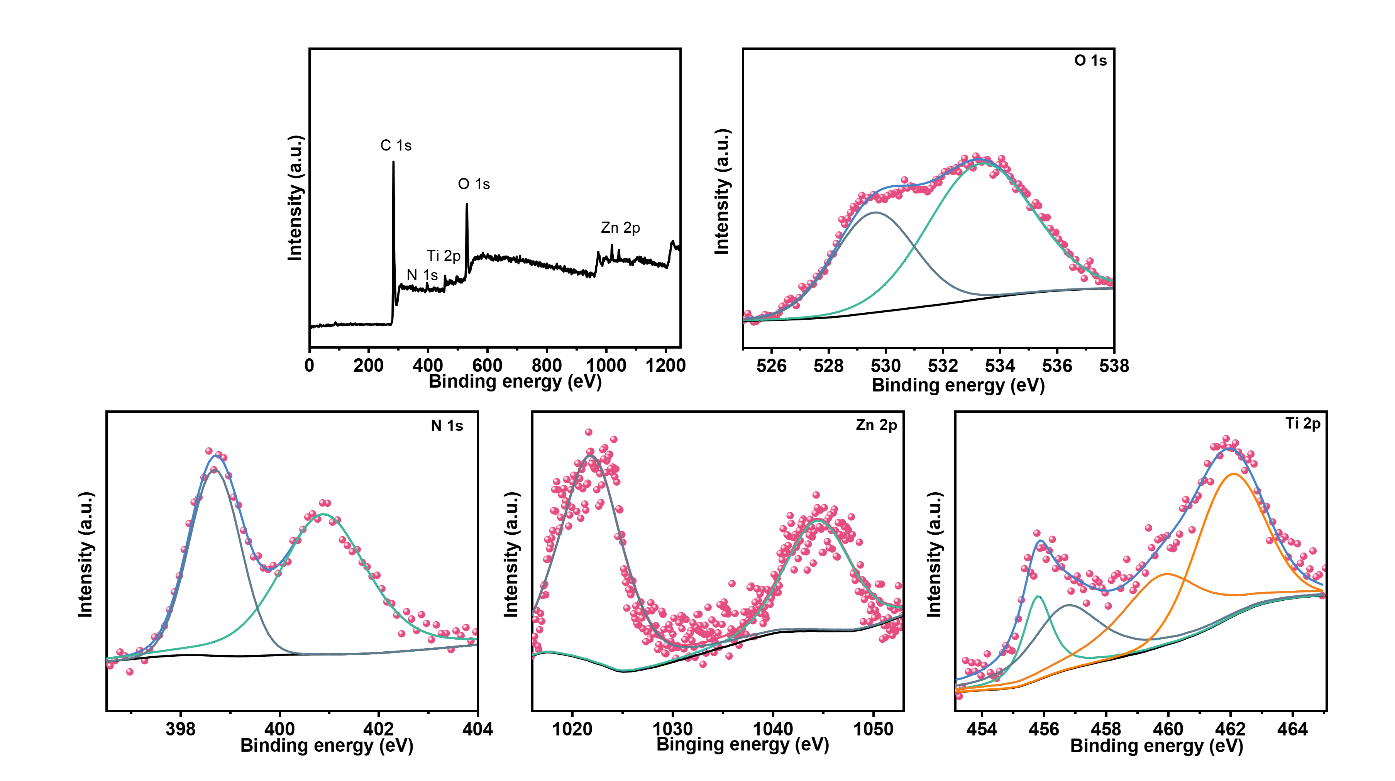


**Figure S3.** Full XPS, O 1s, N 1s, Zn 2p and Ti 2p of ZnTCPP-Ti.


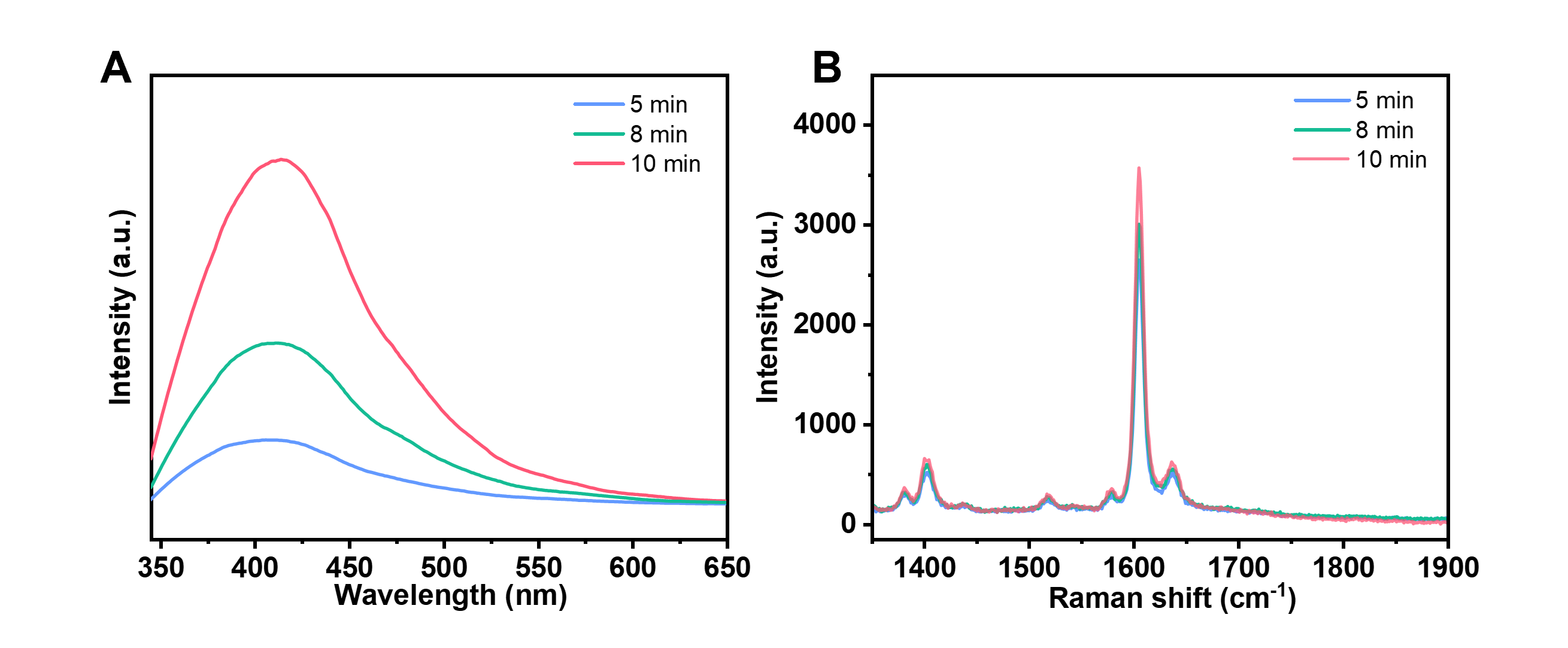


**Figure S4.** (A) Fluorescence spectra of ZnTCPP-Zn+TA and (B) in situ Raman spectroscopy of ZnTCPP-Zn+TMB after 5, 8, and 10 minutes of illumination.


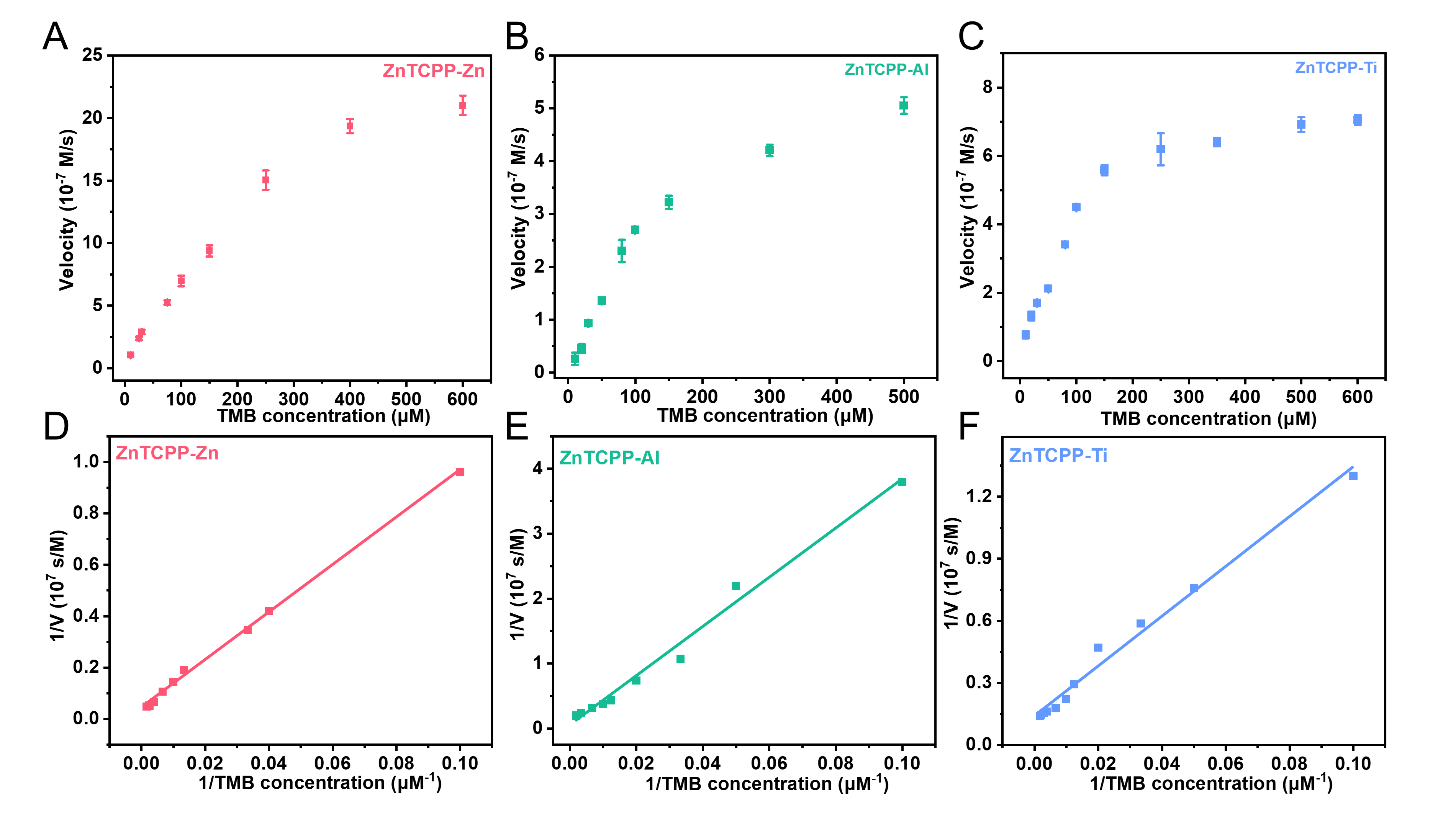


**Figure S5.** Steady-state kinetic assay and Lineweaver−Burk plot of photocatalytic activity of ZnTCPP-Zn (A and D), ZnTCPP-Al (B and E), and ZnTCPP-Ti (C and F) with TMB as the substrate.


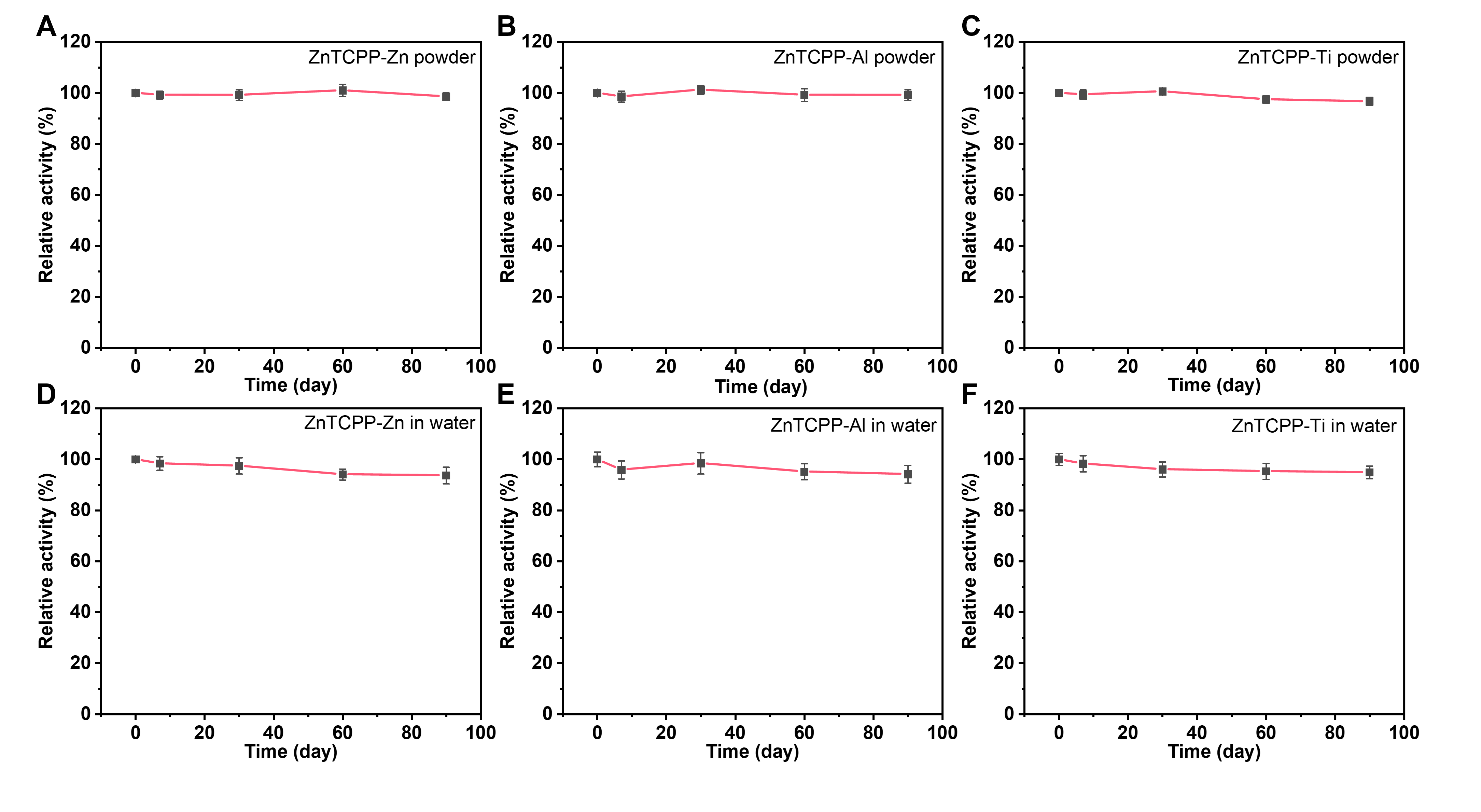


**Figure S6.** Long-term storage stability of ZnTCPP-Zn powder (A) and dispersed in water (D), ZnTCPP-Al powder (B) and dispersed in water (E), ZnTCPP-Al powder (C) and dispersed in water (F).


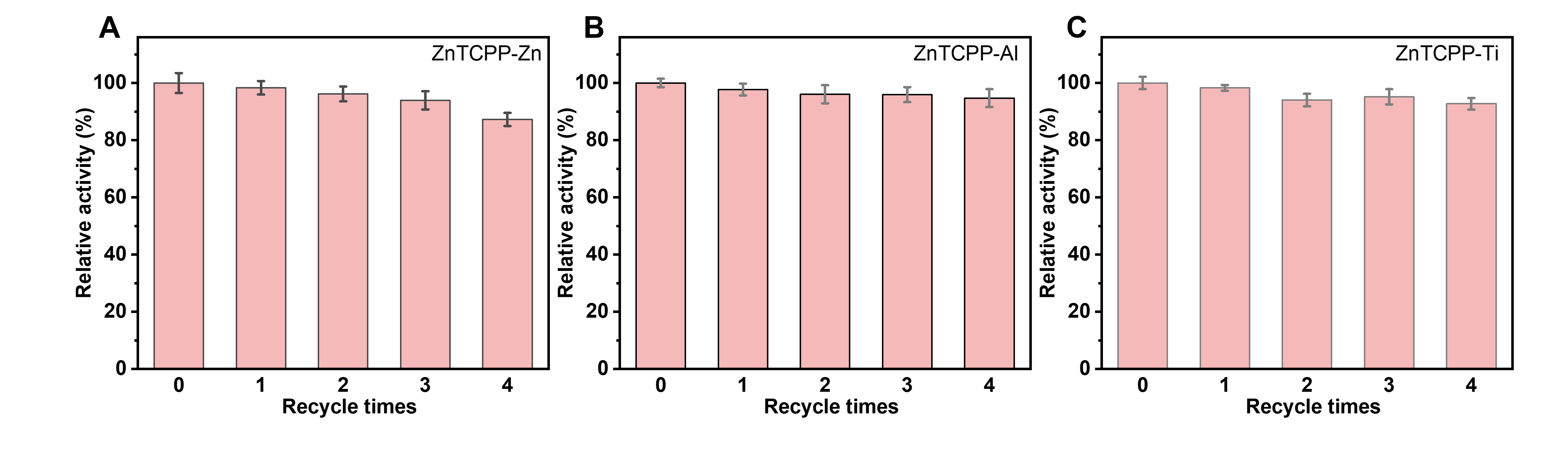


**Figure S7.** Recycling performance teats of (A) ZnTCPP-Zn, (B) ZnTCPP-Al, and (C) ZnTCPP-Ti colorimetric sensor.


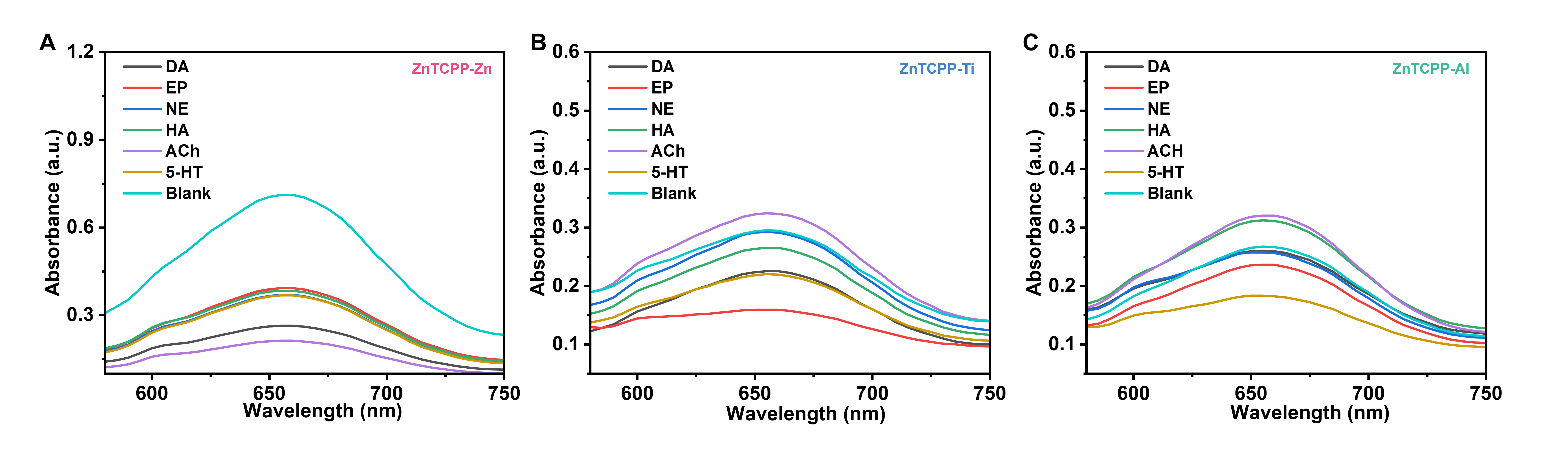


**Figure S8.** UV-vis absorption spectra of TMB catalyzed by ZnTCPP-Zn (A), ZnTCPP-Al (B), and ZnTCPP-Ti (C) in the absence and presence of diverse neurotransmitters at 10 μM.


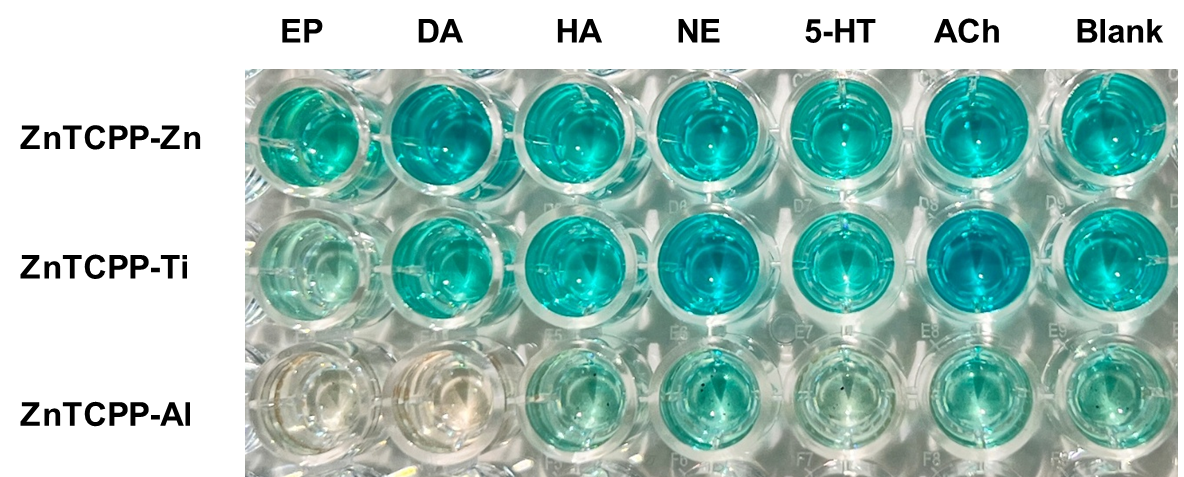


**Figure S9.** Photographic representation of the colorimetric responses of the sensor array upon exposure to different neurotransmitters.


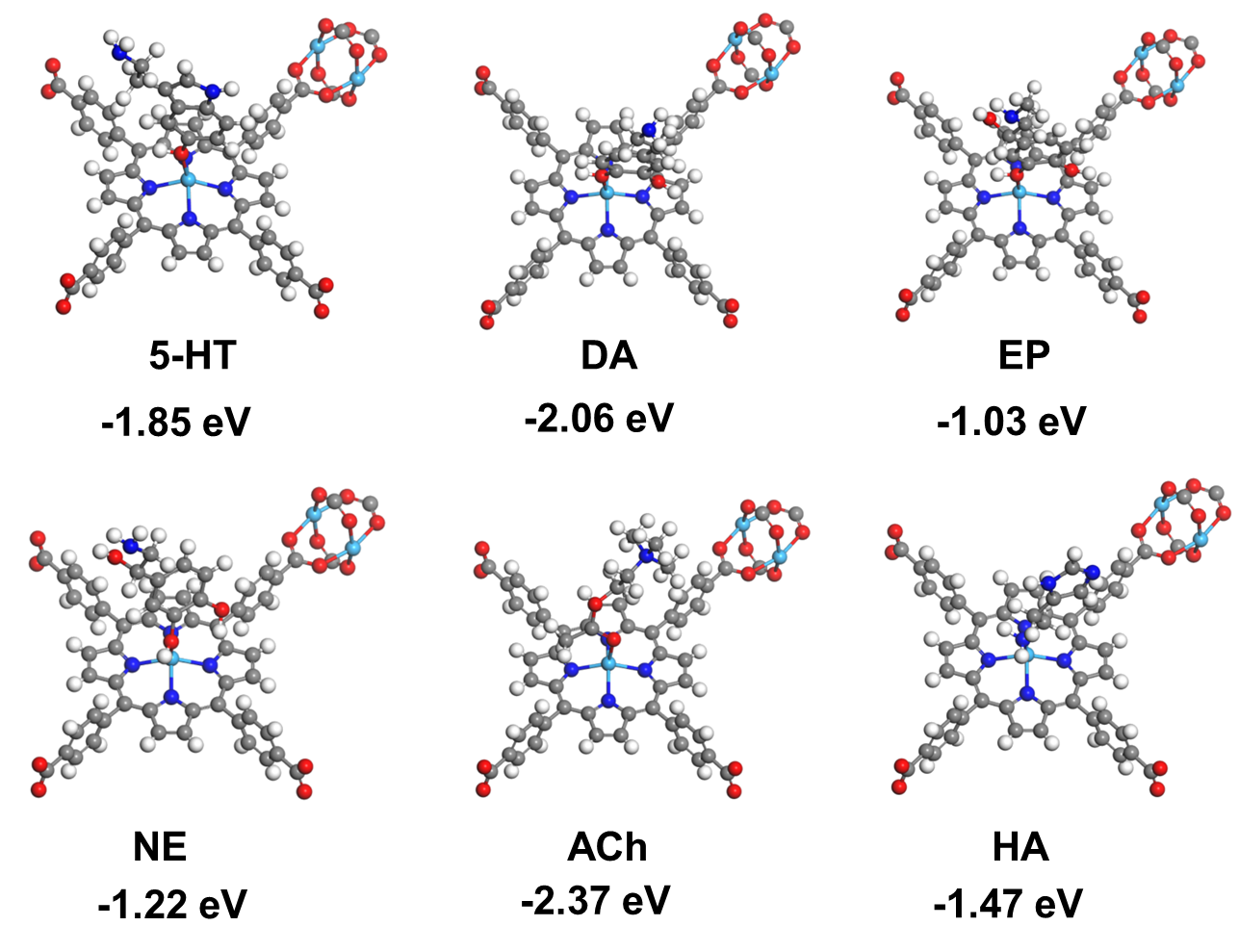


**Figure S10.** Optimized molecular structures of six representative neurotransmitters (5-HT, DA, EP, NE, ACh, and HA) interacting with the ZnTCPP-Zn nanozyme, obtained via DFT calculations.


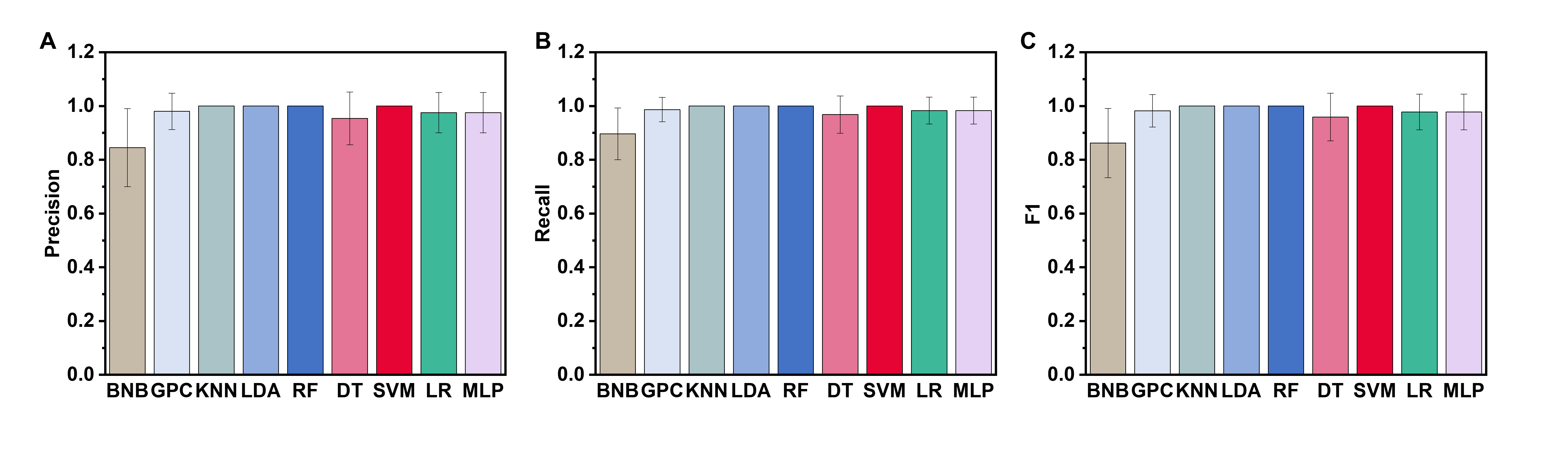


**Figure S11.** Comparison of performance metrics of machine learning algorithms for neurotransmitters classification: (A) precision, (B) recall, and (C) F1 score.


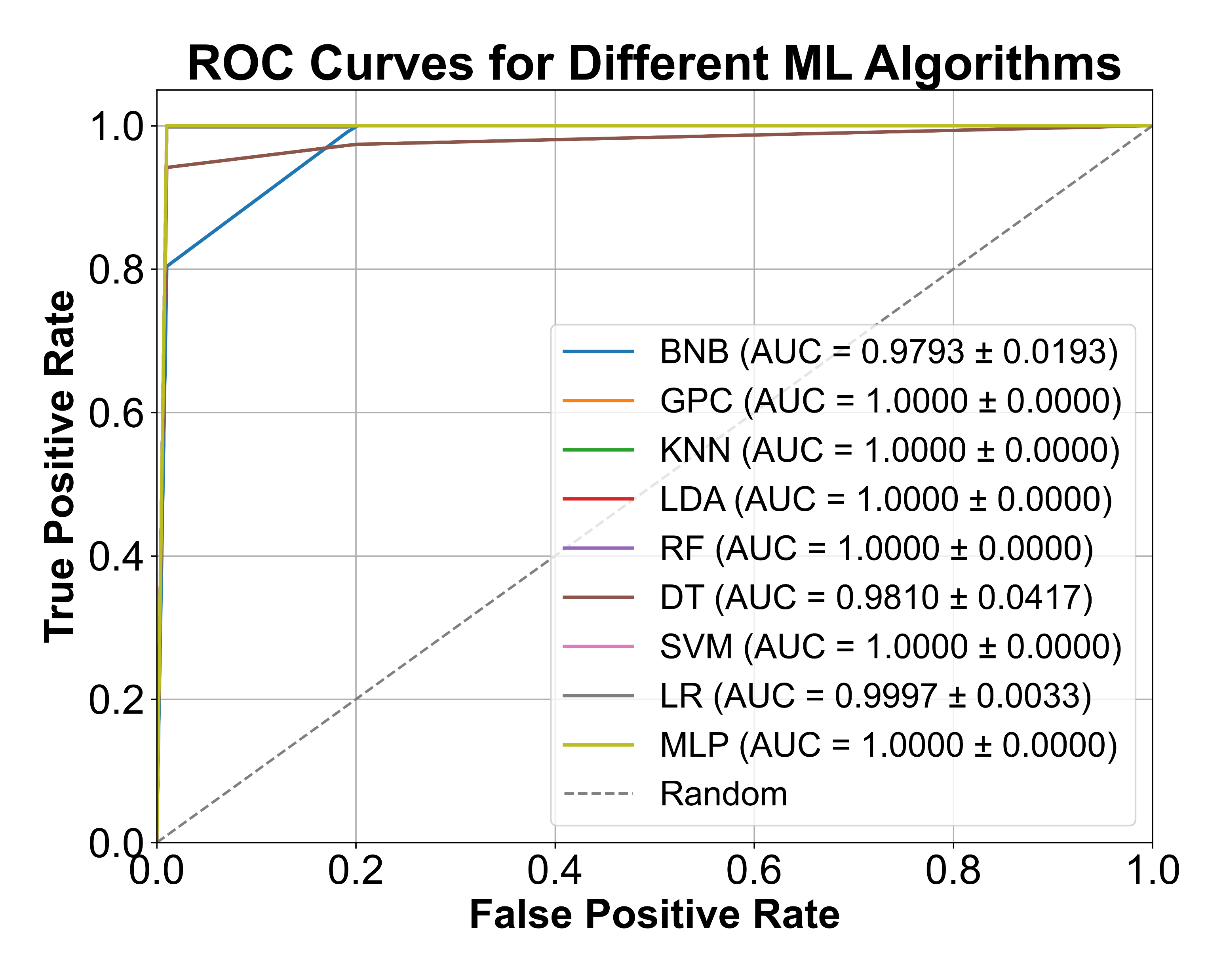


**Figure S12.** Receiver operating characteristic (ROC) curves of different machine learning models for neurotransmitter identification. Random: performance baseline in a random state.

**Figure S13.** Correlations of canonical colorimetric response patterns from the array of nanozymes against 6 neurotransmitters at 10 μM. The 95% confidence ellipses for the individual analytes are shown.


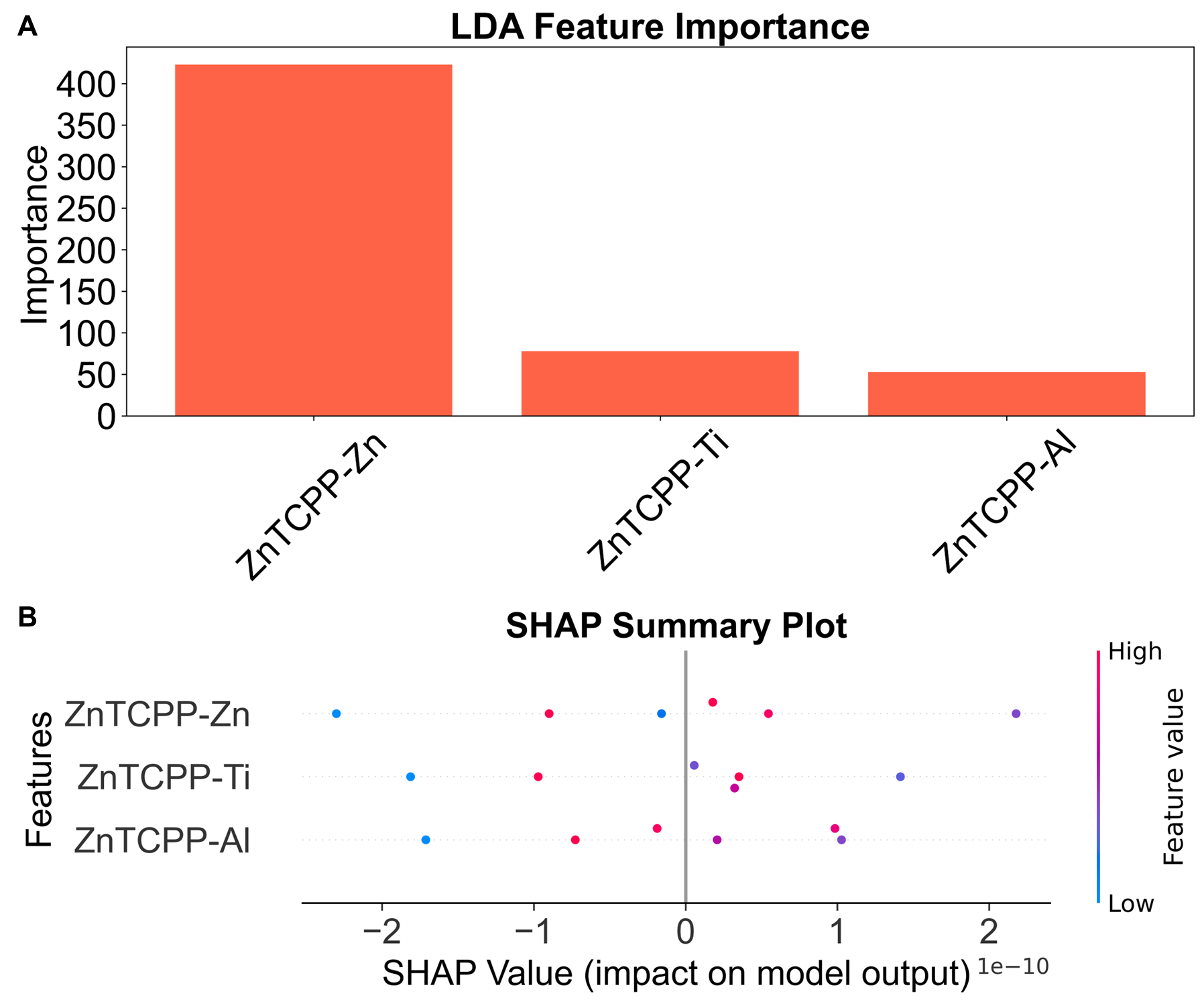


**Figure S14.** Feature contribution analysis of the sensor array using LDA and SHAP. (A) Linear Discriminant Analysis (LDA) revealed the relative importance of each MOF-based nanozyme sensing element in distinguishing analytes. (B) SHAP summary plot based on a Random Forest model.


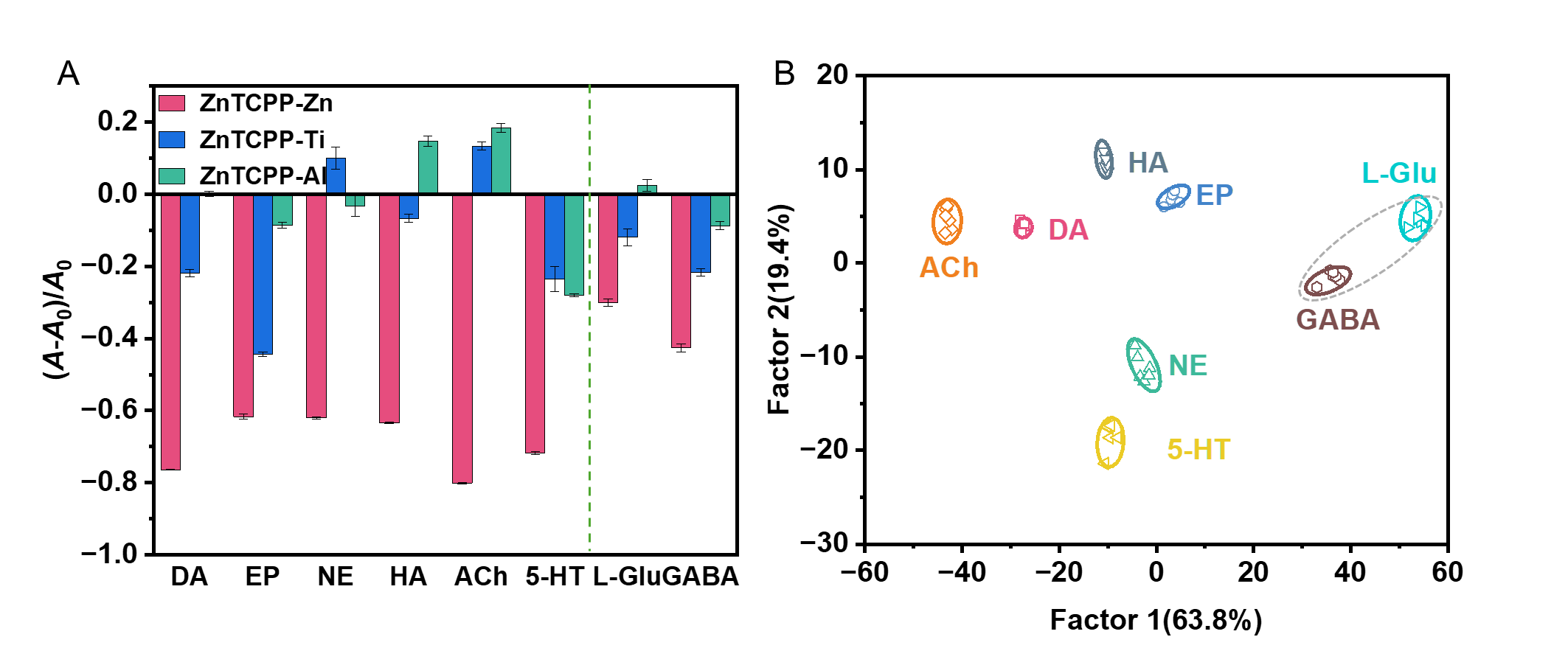


**Figure S15.** (A) Colorimetric response patterns [(*A*- *A*_0_)/*A*_0_] of the sensor array toward eight neurotransmitters. (B) LDA canonical score plot using the first two factors obtained from corimetric response pattern from eight neurotransmitters.


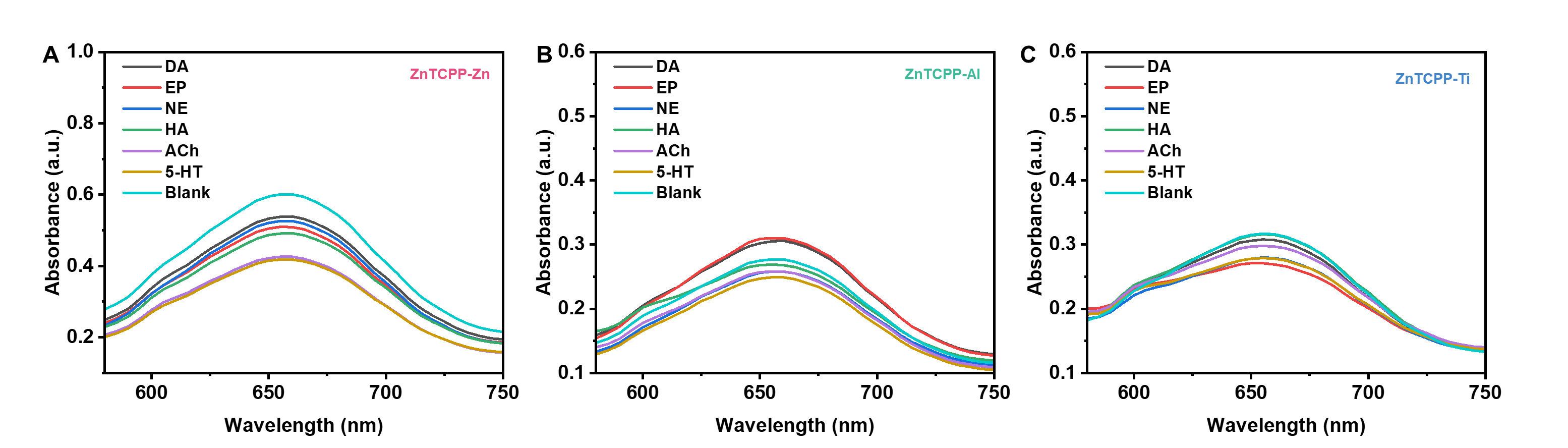


**Figure S16.** UV−vis absorption spectra of TMB catalyzed by ZnTCPP-Zn (A), ZnTCPP-Al (B), and ZnTCPP-Ti (C) in the absence and presence of diverse neurotransmitters at 0.1 μM.


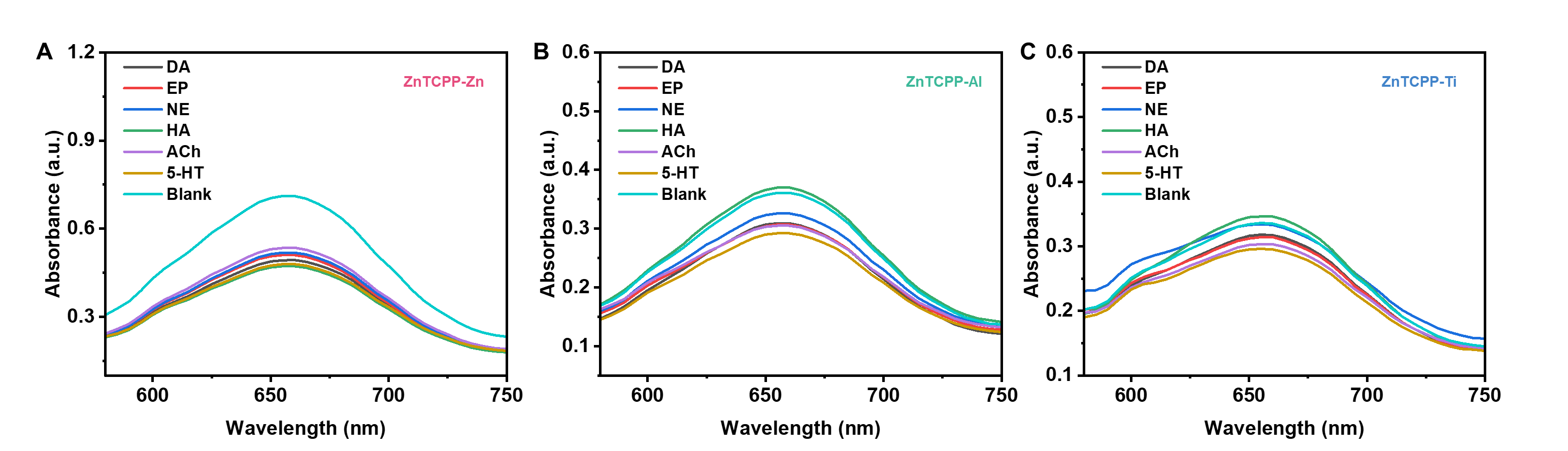


**Figure S17.** UV−vis absorption spectra of TMB catalyzed by ZnTCPP-Zn (A), ZnTCPP-Al (B), and ZnTCPP-Ti (C) in the absence and presence of diverse neurotransmitters at 1 μM.

**Figure S18.** Colorimetric response patterns [(*A*- *A*_0_)/*A*_0_] of the sensor array toward six neurotransmitters at 0.1 μM (A)and 1 μM (C). LDA canonical score plot using the first two factors obtained from corimetric response pattern at 0.1 μM (B)and 1 μM (D).

**Figure S19.** Correlations of canonical colorimetric response patterns from the array of nanozymes against 6 neurotransmitters at 1 μM. The 95% confidence ellipses for the individual analytes are shown.

**Figure S20.** Correlations of canonical colorimetric response patterns from the array of nanozymes against 6 neurotransmitters at 0.1 μM. The 95% confidence ellipses for the individual analytes are shown.

**Figure S21.** Correlations of canonical colorimetric response patterns from the array of nano-enzyme against DA with various concentrations. The 95% confidence ellipses for the individual analytes are shown.

**Figure S22.** Correlations of canonical colorimetric response patterns from the array of nano-enzyme against EP with various concentrations. The 95% confidence ellipses for the individual analytes are shown.

**Figure S23.** Correlations of canonical colorimetric response patterns from the array of nano-enzyme against 5-HT with various concentrations. The 95% confidence ellipses for the individual analytes are shown.

**Figure S24.** Correlations of canonical colorimetric response patterns from the array of nano-enzyme against NE with various concentrations. The 95% confidence ellipses for the individual analytes are shown.

**Figure S25.** Correlations of canonical colorimetric response patterns from the array of nano-enzyme against HA with various concentrations. The 95% confidence ellipses for the individual analytes are shown.

**Figure S26.** Correlations of canonical colorimetric response patterns from the array of nano-enzyme against ACh with various concentrations. The 95% confidence ellipses for the individual analytes are shown.

**
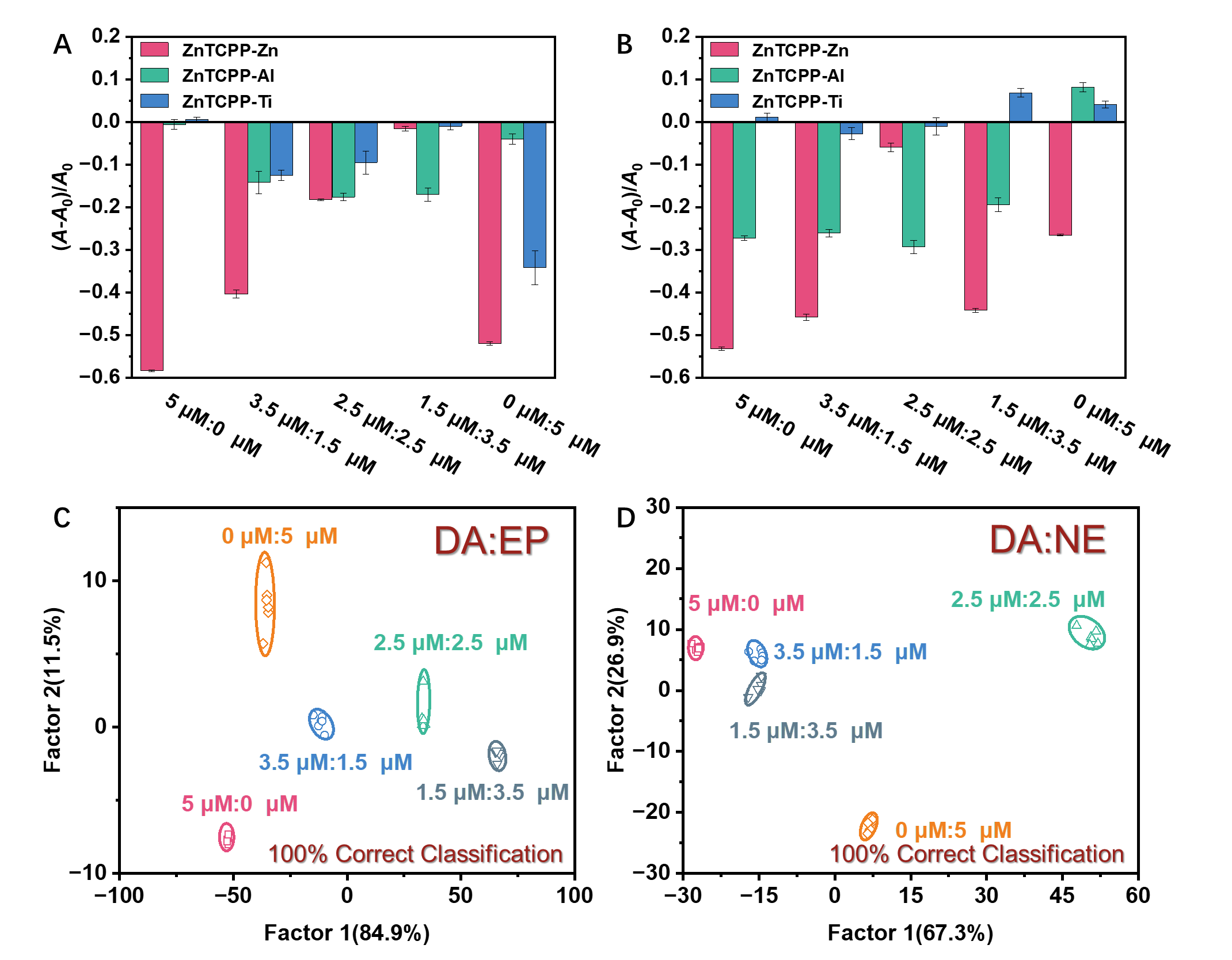
**

**Figure S27.** Colorimetric response patterns [(A- A_0_)/A_0_] of the sensor array toward the DA/EP mixture (A) and DA/NE mixture (B). LDA canonical score plot using the first two factors obtained from corimetric response pattern from the DA/EP mixture (C) and DA/NE mixture (D).

**Figure S28.** Correlations of canonical colorimetric response patterns from the array of nanozymes against DA/EP with different molar ratios. The 95% confidence ellipses for the individual analytes are shown.

**Figure S29.** Correlations of canonical colorimetric response patterns from the array of nanozymes against DA/NE with different molar ratios. The 95% confidence ellipses for the individual analytes are shown.


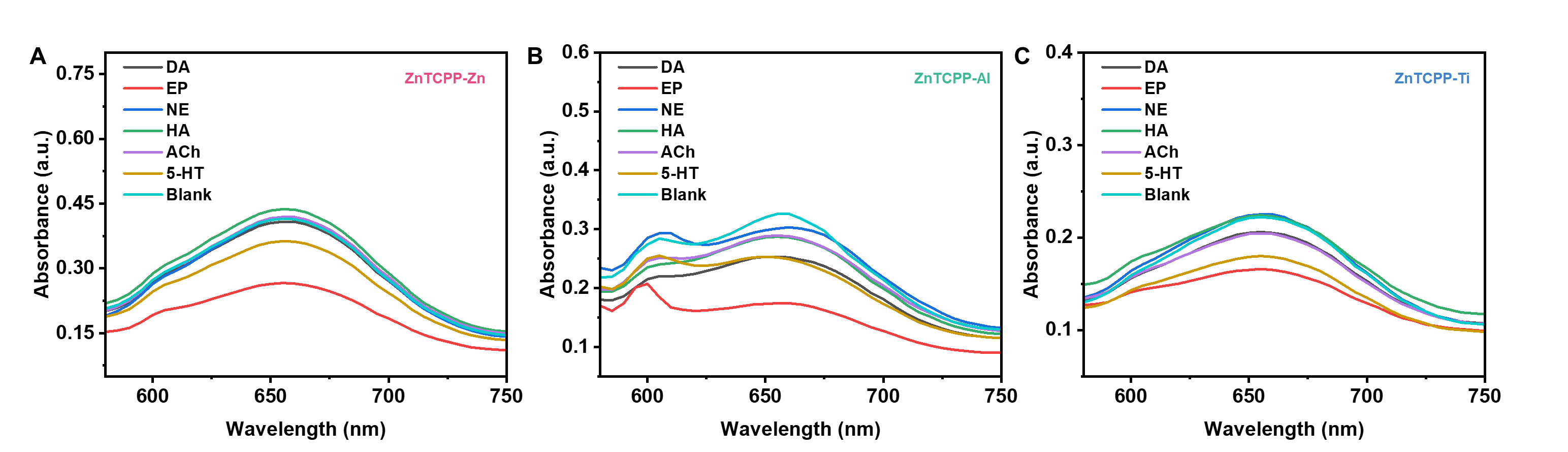


**Figure S30.** UV−vis absorption spectra of TMB catalyzed by ZnTCPP-Zn (A), ZnTCPP-Al (B), and ZnTCPP-Ti (C) in the absence and presence of diverse neurotransmitters in cerebro-spinal fluid.


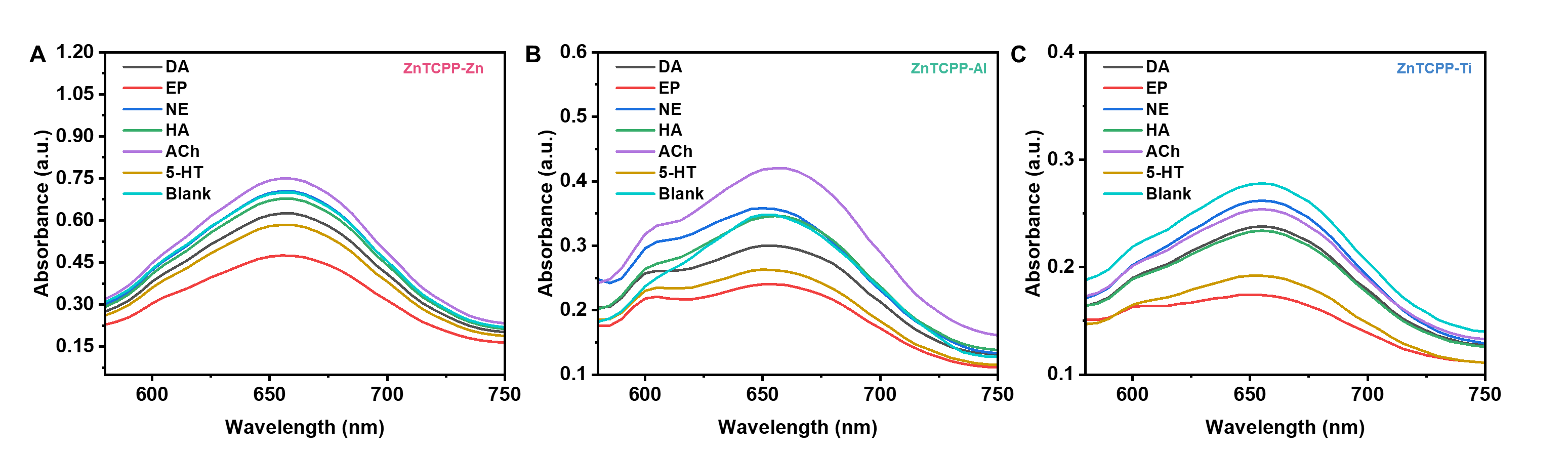


**Figure S31.** UV−vis absorption spectra of TMB catalyzed by ZnTCPP-Zn (A), ZnTCPP-Al (B), and ZnTCPP-Ti (C) in the absence and presence of diverse neurotransmitters in serum.

**Figure S32.** Correlations of canonical colorimetric response patterns from the array of nanozymes against 6 neurotransmitters a in cerebro-spinal fluid. The 95% confidence ellipses for the individual analytes are shown.

**Figure S33.** Correlations of canonical colorimetric response patterns from the array of nanozymes against 6 neurotransmitters a in serum. The 95% confidence ellipses for the individual analytes are shown.


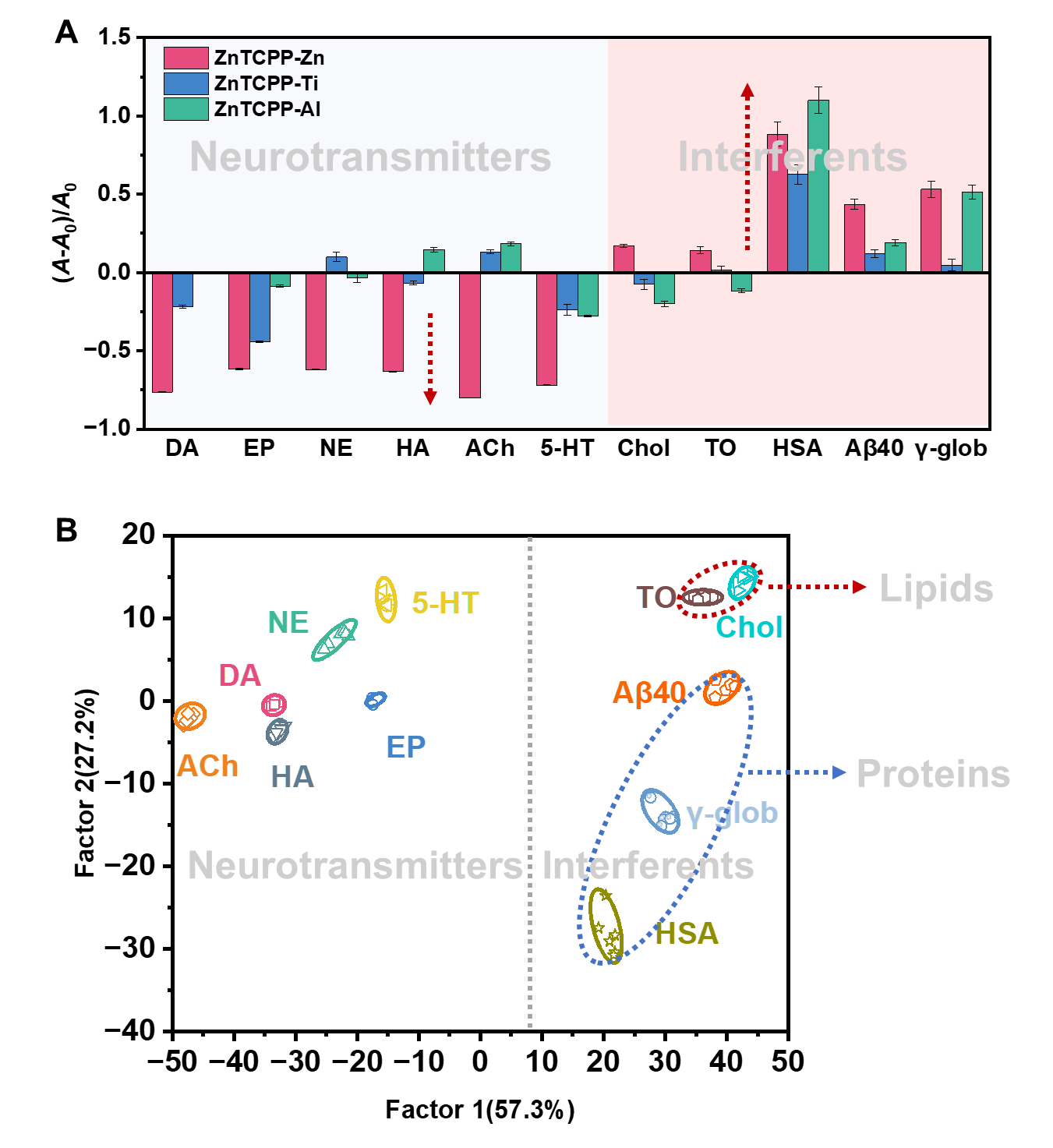


**Figure S34.** (A) Colorimetric response patterns [(*A*- *A*_0_)/*A*_0_] of the sensor array toward six neurotransmitters and other interfering substances. (B) Canonical score plot for the response patterns obtained against the six neurotransmitters and other interfering substances (10 µM).


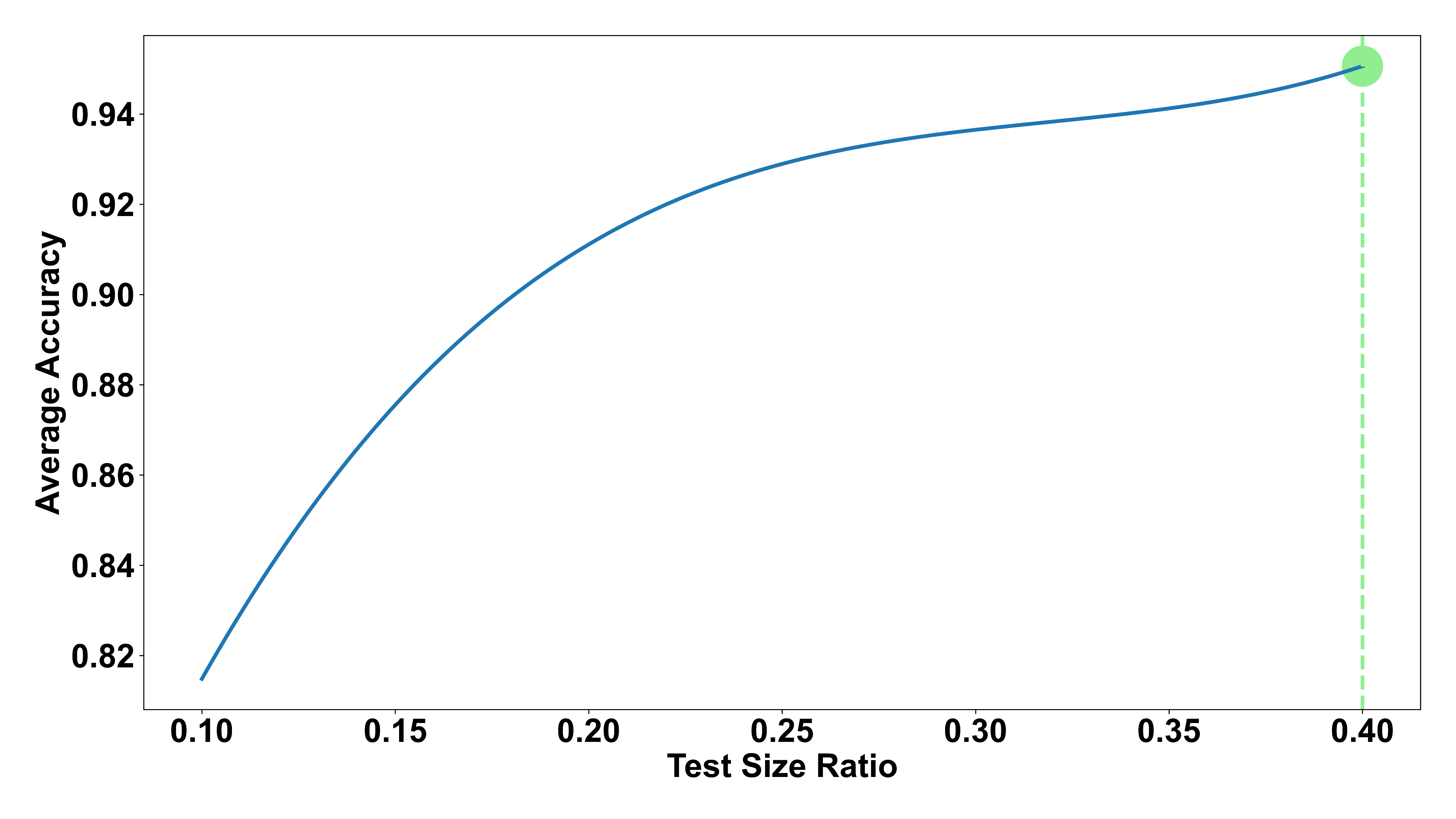


**Figure S35.** Relationship between test set ratio and average classification accuracy.


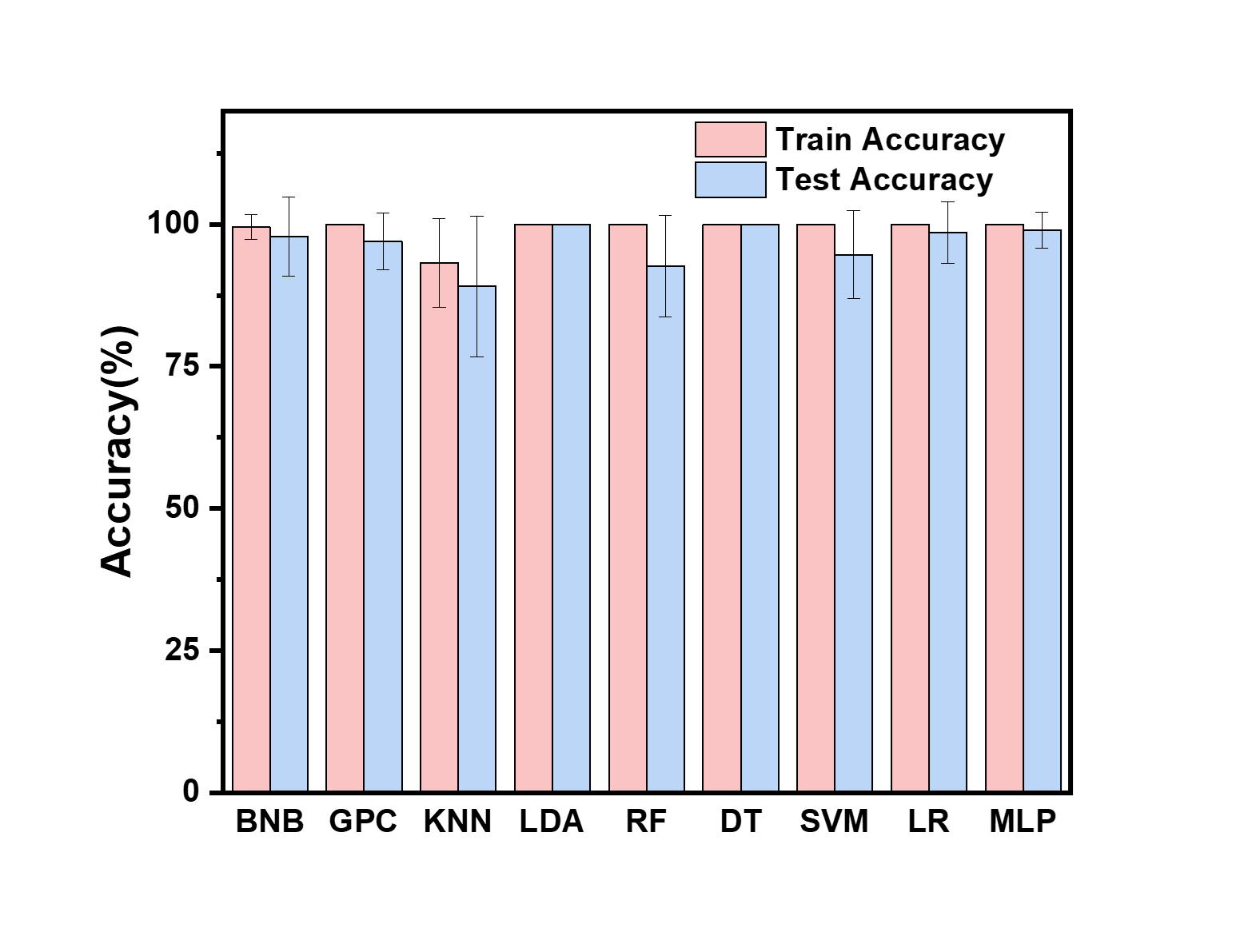


**Figure S36.** Comparison of accuracies for neurological diseases by employing different machine learning algorithms on the training and prediction.


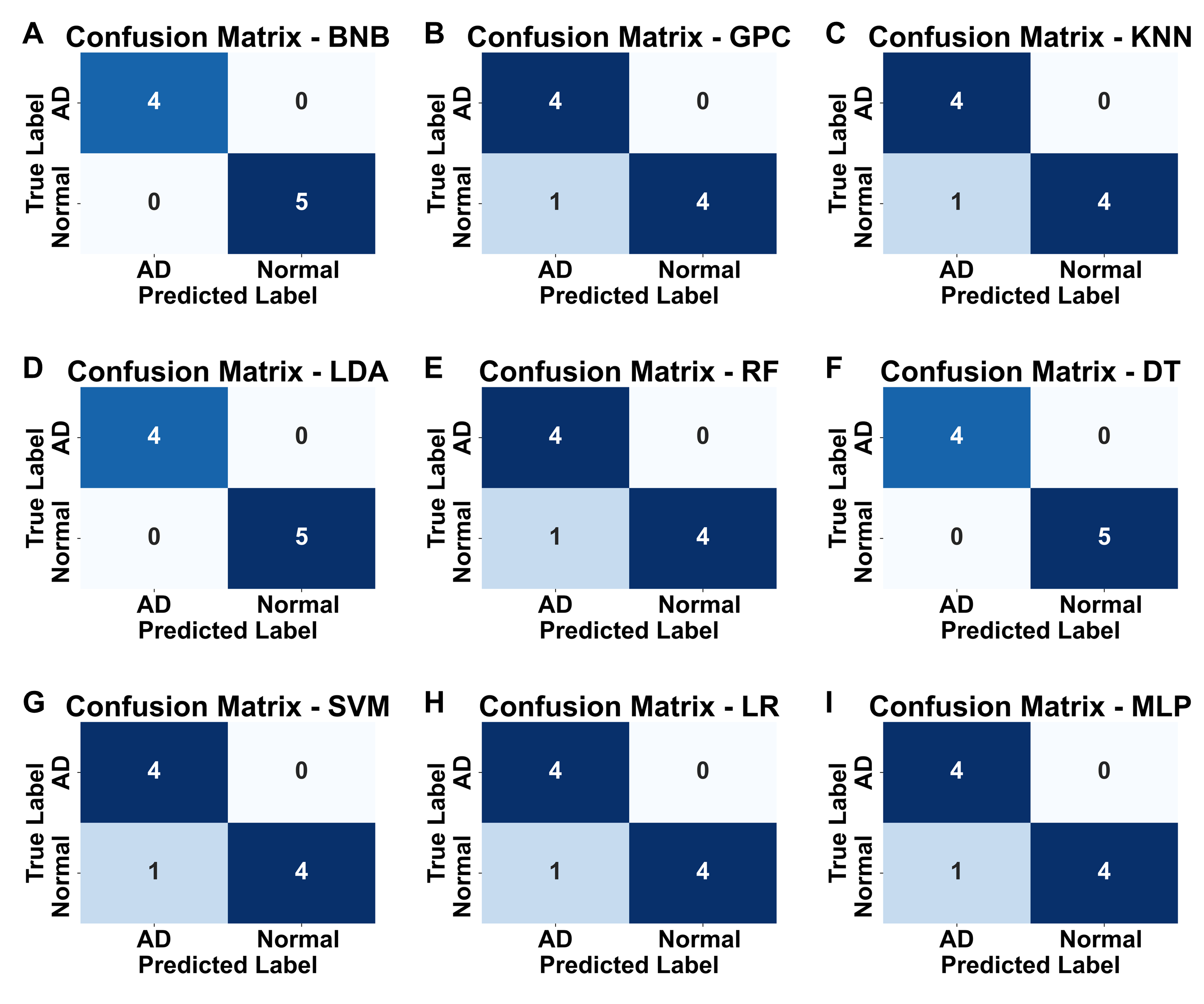


**Figure S37.** Confusion matrices of test set predictions for AD versus normal classification using nine different machine learning algorithms.


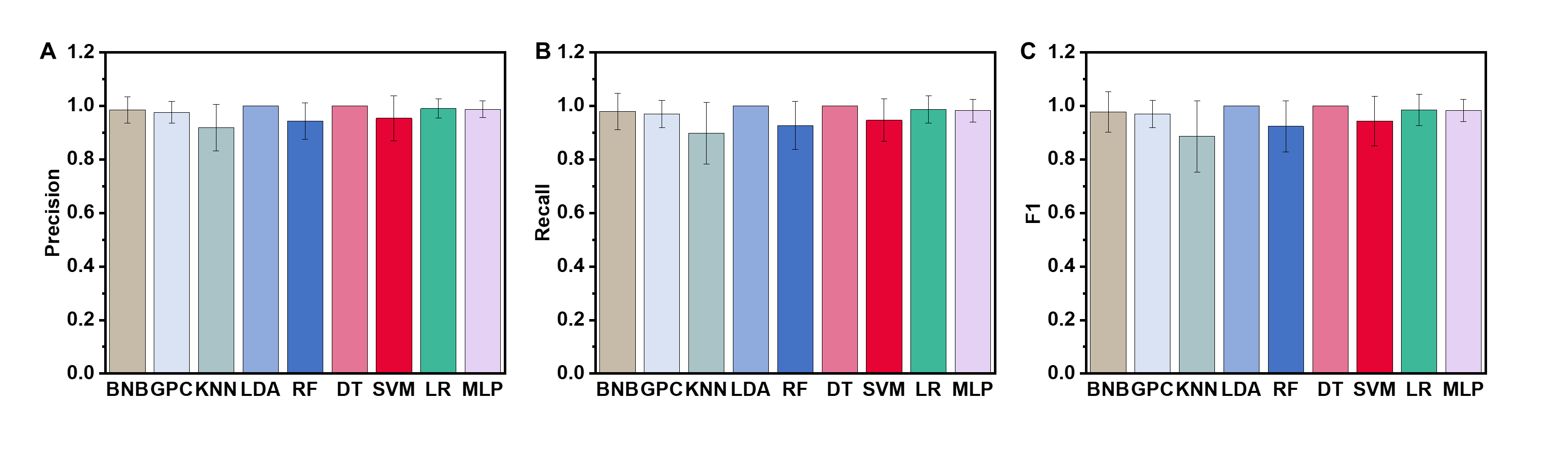


**Figure S38.** Comparison of performance metrics of machine learning algorithms for AD diagnosis: (A) precision, (B) recall, and (C) F1 score.


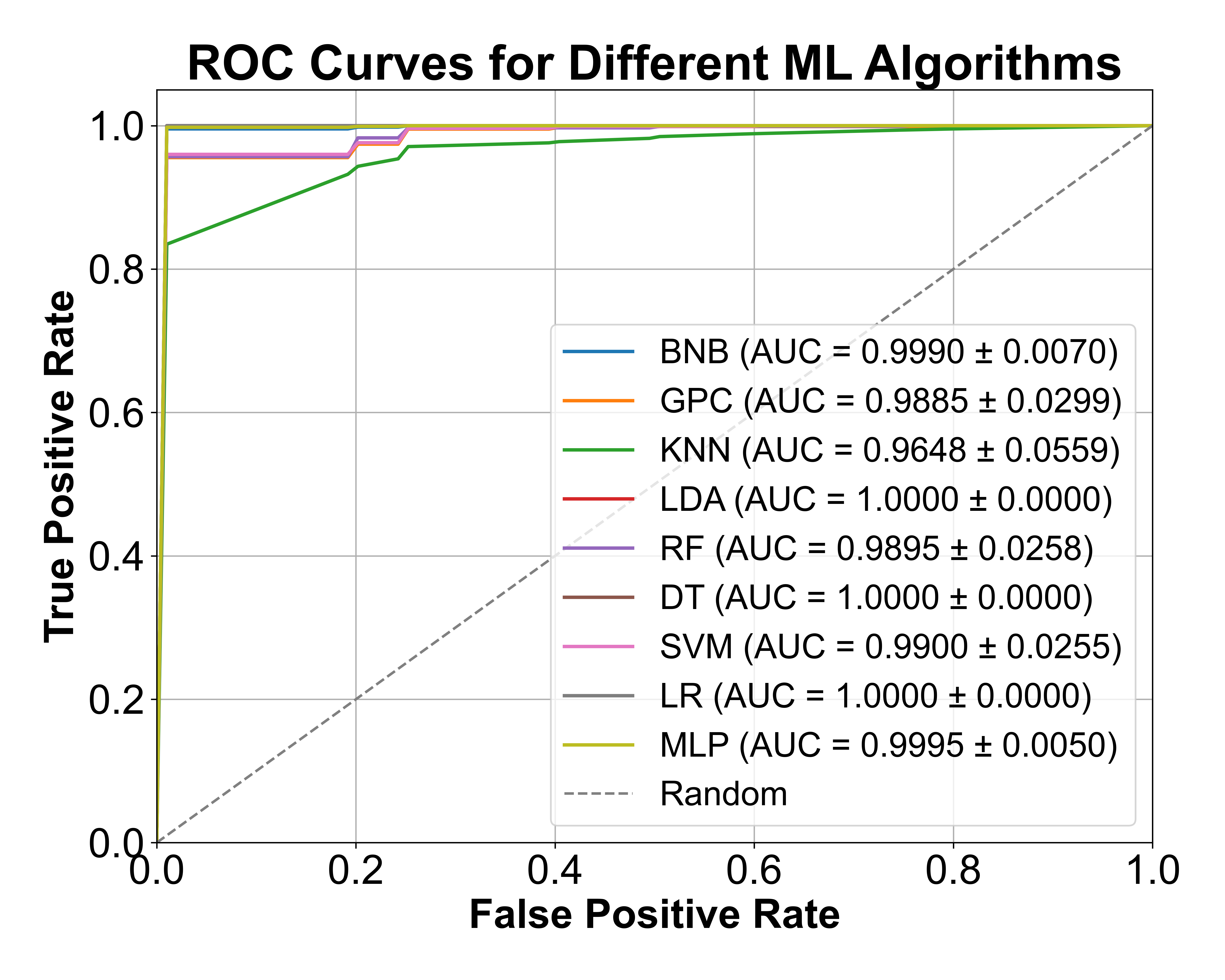


**Figure S39.** Receiver operating characteristic (ROC) curves of different machine learning models for AD diagnosis. Random: performance baseline in a random state.


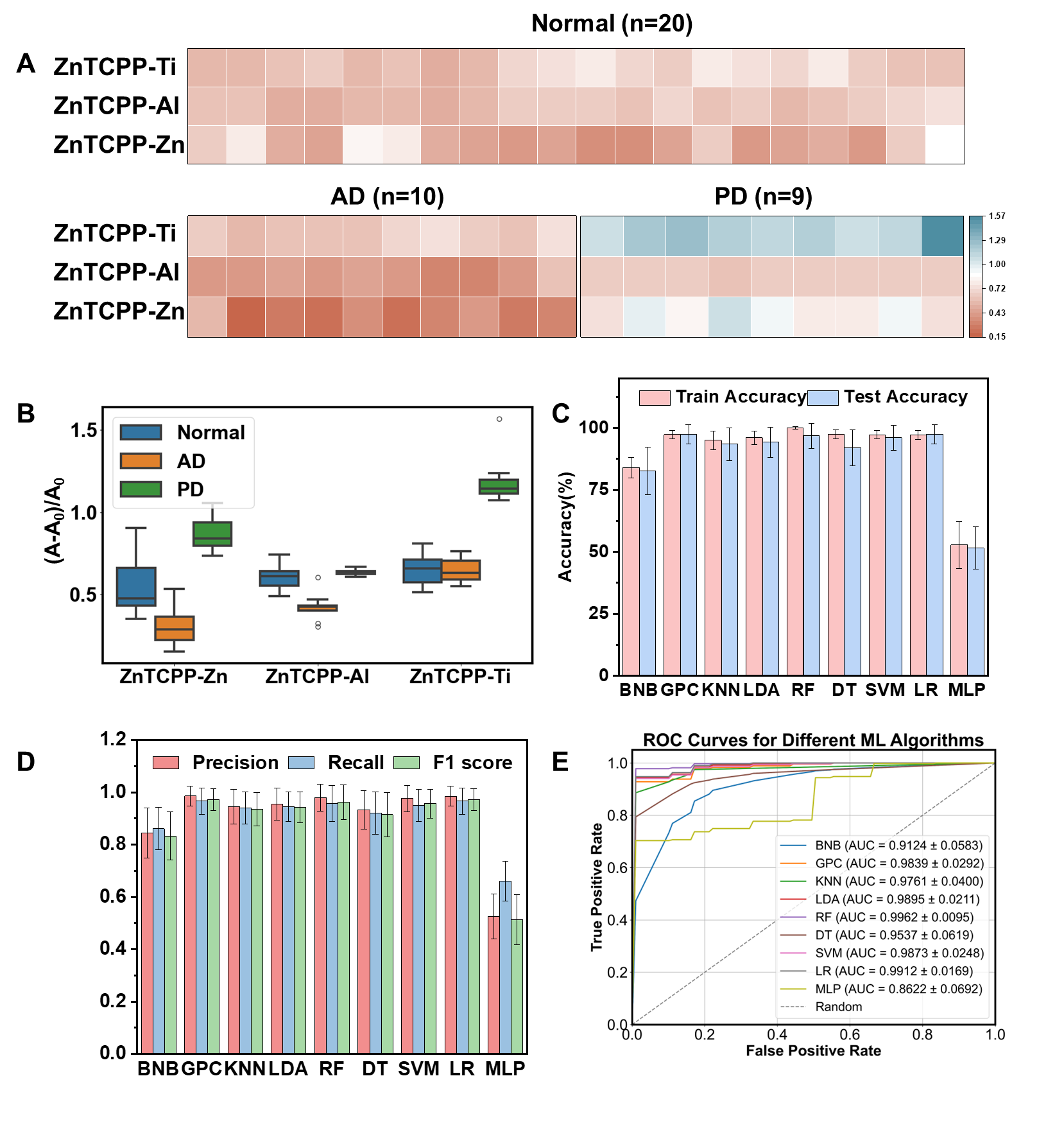


**Figure S40.** (A) Heatmap derived from the changes of the array signal responses toward clinical serum samples from healthy controls, AD, and PD patients. (B) Box plots of absorbance responses for each nanozyme across different groups. (C) Comparison of accuracies for clinical serum samples by employing different machine learning algorithms on the training and prediction. (D) Comparison of performance metrics of machine learning algorithms for clinical serum samples classification. (E) Receiver operating characteristic (ROC) curves of different machine learning models for clinical serum samples identification. Random: performance baseline in a random state.

# Supporting Tables

**Table S1.** Kinetic parameters of ZnTCPP-Zn, ZnTCPP-Al and ZnTCPP-Ti.

| MOFs | *v*_max_(M/s) | *K*_m_(mM) |
| --- | --- | --- |
| ZnTCPP-Zn | 2.384×10^-6^ | 0.219 |
| ZnTCPP-Al | 2.752×10^-6^ | 1.041 |
| ZnTCPP-Ti | 7.388×10^-7^ | 0.089 |

**Table S2**. Training matrix of the colorimetric response patterns obtained from an array of nanozymes against 6 neurotransmitters at 10 μM. LDA was carried out and resulting in 3 factors of the canonical scores.

|  | Colorimetric response patterns | | | LDA canonical scores | | | |
| --- | --- | --- | --- | --- | --- | --- | --- |
|  | ZnTCPP-Zn | ZnTCPP-Ti | ZnTCPP-Al | Factor 1 | Factor 2 | Factor 3 | Group |
| DA | -0.76 | -0.24 | -0.01 | -20.80 | -2.40 | -8.47 | 3 |
| DA | -0.76 | -0.22 | 0.00 | -20.77 | -1.77 | -7.59 | 3 |
| DA | -0.76 | -0.22 | 0.01 | -21.33 | -1.27 | -7.84 | 3 |
| DA | -0.76 | -0.21 | 0.00 | -20.80 | -2.20 | -6.90 | 3 |
| DA | -0.76 | -0.22 | 0.00 | -20.37 | -1.39 | -7.65 | 3 |
| DA | -0.76 | -0.21 | 0.00 | -20.06 | -1.38 | -7.42 | 3 |
| EP | -0.63 | -0.45 | -0.10 | 17.26 | 0.79 | -14.33 | 4 |
| EP | -0.62 | -0.45 | -0.09 | 20.59 | 1.80 | -14.51 | 4 |
| EP | -0.62 | -0.44 | -0.09 | 19.89 | 2.09 | -13.99 | 4 |
| EP | -0.62 | -0.44 | -0.08 | 20.45 | 2.75 | -13.95 | 4 |
| EP | -0.61 | -0.44 | -0.09 | 22.48 | 2.28 | -13.66 | 4 |
| EP | -0.61 | -0.45 | -0.08 | 20.90 | 2.97 | -14.49 | 4 |
| NE | -0.63 | 0.10 | -0.05 | 21.41 | -1.41 | 15.16 | 6 |
| NE | -0.62 | 0.05 | -0.06 | 23.34 | -1.36 | 13.09 | 6 |
| NE | -0.62 | 0.09 | -0.05 | 22.49 | -1.76 | 15.26 | 6 |
| NE | -0.62 | 0.14 | 0.00 | 22.08 | 1.52 | 16.29 | 6 |
| NE | -0.62 | 0.13 | 0.01 | 21.31 | 2.36 | 15.52 | 6 |
| NE | -0.62 | 0.09 | -0.05 | 23.91 | -1.10 | 14.90 | 6 |
| HA | -0.64 | -0.08 | 0.13 | 10.40 | 11.98 | -0.43 | 5 |
| HA | -0.64 | -0.07 | 0.14 | 10.07 | 12.68 | -0.18 | 5 |
| HA | -0.63 | -0.06 | 0.15 | 10.95 | 13.91 | 0.10 | 5 |
| HA | -0.63 | -0.07 | 0.14 | 10.77 | 13.06 | -0.01 | 5 |
| HA | -0.63 | -0.06 | 0.16 | 10.30 | 14.59 | 0.13 | 5 |
| HA | -0.63 | -0.05 | 0.16 | 10.90 | 14.06 | 0.58 | 5 |
| Ach | -0.80 | 0.13 | 0.17 | -33.35 | 4.34 | 6.39 | 2 |
| Ach | -0.80 | 0.12 | 0.18 | -33.98 | 4.91 | 5.74 | 2 |
| Ach | -0.80 | 0.13 | 0.20 | -34.61 | 6.15 | 5.77 | 2 |
| Ach | -0.80 | 0.13 | 0.20 | -34.21 | 6.53 | 5.71 | 2 |
| Ach | -0.80 | 0.13 | 0.18 | -35.05 | 5.26 | 5.64 | 2 |
| Ach | -0.80 | 0.15 | 0.18 | -34.78 | 4.36 | 7.43 | 2 |
| 5-HT | -0.72 | -0.17 | -0.28 | 1.52 | -19.72 | 4.39 | 1 |
| 5-HT | -0.72 | -0.24 | -0.28 | 1.73 | -18.84 | 0.68 | 1 |
| 5-HT | -0.71 | -0.24 | -0.28 | 3.99 | -18.62 | 0.60 | 1 |
| 5-HT | -0.72 | -0.25 | -0.28 | 1.15 | -18.42 | -0.45 | 1 |
| 5-HT | -0.72 | -0.25 | -0.27 | 0.77 | -18.24 | -0.39 | 1 |
| 5-HT | -0.72 | -0.27 | -0.28 | 1.44 | -18.49 | -1.11 | 1 |

**Table S3.** Predictive performance of different machine learning models for neurotransmitter classification.

| Model | Precision | Recall | F1 score | AUC |
| --- | --- | --- | --- | --- |
| BNB | 0.8450±0.1448 | 0.8967±0.0966 | 0.8622±0.1287 | 0.9793±0.0193 |
| GPC | 0.9800±0.0678 | 0.9867±0.0452 | 0.9822±0.0603 | 1.0000±0.0000 |
| KNN | 1.0000±0.0000 | 1.0000±0.0000 | 1.0000±0.0000 | 1.0000±0.0000 |
| LDA | 1.0000±0.0000 | 1.0000±0.0000 | 1.0000±0.0000 | 1.0000±0.0000 |
| RF | 1.0000±0.0000 | 1.0000±0.0000 | 1.0000±0.0000 | 1.0000±0.0000 |
| DT | 0.9542±0.0982 | 0.9683±0.0695 | 0.9589±0.0884 | 0.9810+0.0417 |
| SVM | 1.0000±0.0000 | 1.0000±0.0000 | 1.0000±0.0000 | 1.0000±0.0000 |
| LR | 0.9750±0.0750 | 0.9833±0.0500 | 0.9778±0.0667 | 0.9997+0.0033 |
| MLP | 0.9750±0.0750 | 0.9833±0.0500 | 0.9778±0.0667 | 1.0000±0.0000 |

**Table S4**. LDA jackknifed classification matrix table obtained from an array of nanozymes against 6 neurotransmitters at 10 μM. The jackknifed classification matrix with cross-validation reveals a 100% accuracy.

|  | 5-HT | Ach | DA | EP | HA | NE | %correct |
| --- | --- | --- | --- | --- | --- | --- | --- |
| 5-HT | 6 | 0 | 0 | 0 | 0 | 0 | 100 |
| Ach | 0 | 6 | 0 | 0 | 0 | 0 | 100 |
| DA | 0 | 0 | 6 | 0 | 0 | 0 | 100 |
| EP | 0 | 0 | 0 | 6 | 0 | 0 | 100 |
| HA | 0 | 0 | 0 | 0 | 6 | 0 | 100 |
| NE | 0 | 0 | 0 | 0 | 0 | 6 | 100 |
| Total | 6 | 6 | 6 | 6 | 6 | 6 | 100 |

**Table S5**. Detection and identification of unknown samples at 10 μM using LDA from the array of nanozymes. According to the verification, 24 among 24 unknown samples were correctly identified, representing an accuracy of 100%.

| Sample | Colorimetric response patterns | | | Results LDA | | | Analyte | |
| --- | --- | --- | --- | --- | --- | --- | --- | --- |
| # | ZnTCPP-Zn | ZnTCPP-Ti | ZnTCPP-Al | Factor 1 | Factor 2 | Factor 3 | Identification | Verification |
| 1 | -0.76 | -0.20 | 0.01 | -20.62 | -1.36 | -6.79 | 3 | 3 |
| 2 | -0.76 | -0.21 | 0.01 | -19.33 | -1.03 | -7.06 | 3 | 3 |
| 3 | -0.76 | -0.22 | 0.01 | -20.66 | -0.85 | -7.87 | 3 | 3 |
| 4 | -0.76 | -0.20 | 0.00 | -20.21 | -1.58 | -6.65 | 3 | 3 |
| 5 | -0.62 | -0.45 | -0.06 | 19.50 | 3.90 | -15.02 | 4 | 4 |
| 6 | -0.62 | -0.45 | -0.08 | 19.47 | 2.79 | -15.02 | 4 | 4 |
| 7 | -0.62 | -0.47 | -0.07 | 19.18 | 3.81 | -16.13 | 4 | 4 |
| 8 | -0.61 | -0.45 | -0.07 | 20.48 | 3.19 | -14.63 | 4 | 4 |
| 9 | -0.61 | 0.13 | 0.10 | 19.32 | 8.91 | 12.86 | 6 | 6 |
| 10 | -0.61 | 0.04 | 0.03 | 22.13 | 5.37 | 10.30 | 6 | 6 |
| 11 | -0.62 | 0.11 | 0.01 | 22.06 | 3.07 | 14.32 | 6 | 6 |
| 12 | -0.61 | 0.08 | -0.02 | 23.69 | 0.99 | 13.99 | 6 | 6 |
| 13 | -0.63 | -0.07 | 0.01 | 15.57 | 4.06 | 3.91 | 5 | 5 |
| 14 | -0.63 | -0.07 | 0.16 | 10.77 | 14.22 | -0.22 | 5 | 5 |
| 15 | -0.63 | -0.03 | 0.14 | 12.39 | 12.54 | 2.59 | 5 | 5 |
| 16 | -0.63 | -0.06 | 0.15 | 11.25 | 13.92 | 0.32 | 5 | 5 |
| 17 | -0.80 | 0.14 | 0.18 | -34.62 | 5.12 | 6.66 | 2 | 2 |
| 18 | -0.80 | 0.12 | 0.18 | -34.67 | 5.08 | 5.58 | 2 | 2 |
| 19 | -0.80 | 0.14 | 0.16 | -34.17 | 3.71 | 7.20 | 2 | 2 |
| 20 | -0.80 | 0.15 | 0.17 | -34.09 | 4.19 | 7.59 | 2 | 2 |
| 21 | -0.72 | -0.25 | -0.29 | 0.56 | -19.52 | 0.18 | 1 | 1 |
| 22 | -0.72 | -0.24 | -0.28 | 0.48 | -19.33 | 0.47 | 1 | 1 |
| 23 | -0.72 | -0.24 | -0.30 | 1.49 | -20.08 | 1.06 | 1 | 1 |
| 24 | -0.72 | -0.18 | -0.30 | 2.58 | -20.62 | 4.49 | 1 | 1 |

**Table S6**. Training matrix of the colorimetric response patterns obtained from an array of nanozymes against 6 neurotransmitters at 1 μM. LDA was carried out and resulting in 3 factors of the canonical scores.

|  | Colorimetric response patterns | | | LDA canonical scores | | | |
| --- | --- | --- | --- | --- | --- | --- | --- |
|  | ZnTCPP-Zn | ZnTCPP-Ti | ZnTCPP-Al | Factor 1 | Factor 2 | Factor 3 | Group |
| DA | -0.33 | -0.17 | -0.07 | 1.46 | -3.36 | -1.07 | 3 |
| DA | -0.31 | -0.15 | -0.05 | 0.41 | -1.50 | -1.29 | 3 |
| DA | -0.32 | -0.15 | -0.09 | 0.25 | -2.90 | 1.88 | 3 |
| DA | -0.31 | -0.15 | -0.06 | 1.02 | -1.63 | -0.72 | 3 |
| DA | -0.32 | -0.15 | -0.05 | 0.01 | -1.97 | -1.82 | 3 |
| DA | -0.32 | -0.15 | -0.04 | -0.41 | -1.89 | -2.81 | 3 |
| EP | -0.28 | -0.17 | -0.10 | 8.39 | 1.03 | 2.24 | 4 |
| EP | -0.28 | -0.17 | -0.07 | 8.17 | 1.06 | -0.08 | 4 |
| EP | -0.27 | -0.14 | -0.06 | 5.62 | 2.70 | 1.01 | 4 |
| EP | -0.28 | -0.16 | -0.08 | 7.07 | 0.93 | 0.85 | 4 |
| EP | -0.28 | -0.17 | -0.07 | 8.06 | 1.63 | 0.12 | 4 |
| EP | -0.28 | -0.17 | -0.08 | 7.88 | 1.13 | 1.00 | 4 |
| NE | -0.30 | -0.12 | -0.02 | -0.96 | 0.97 | -1.81 | 6 |
| NE | -0.29 | -0.11 | -0.03 | -1.17 | 2.31 | -0.05 | 6 |
| NE | -0.29 | -0.11 | -0.04 | -1.47 | 1.64 | 0.13 | 6 |
| NE | -0.28 | -0.10 | -0.01 | -2.51 | 3.13 | -1.00 | 6 |
| NE | -0.28 | -0.10 | -0.02 | -0.68 | 3.12 | -0.20 | 6 |
| NE | -0.28 | -0.10 | -0.04 | 0.24 | 3.30 | 1.11 | 6 |
| HA | -0.36 | 0.01 | 0.02 | -25.53 | -1.63 | 0.60 | 5 |
| HA | -0.34 | 0.02 | 0.05 | -25.46 | 0.17 | -0.09 | 5 |
| HA | -0.33 | 0.02 | 0.03 | -23.25 | 1.35 | 1.22 | 5 |
| HA | -0.33 | 0.04 | 0.05 | -26.51 | 2.41 | 1.50 | 5 |
| HA | -0.33 | 0.02 | 0.06 | -24.08 | 1.69 | -1.21 | 5 |
| HA | -0.33 | 0.01 | 0.05 | -23.87 | 1.28 | -0.71 | 5 |
| Ach | -0.25 | -0.19 | -0.07 | 14.71 | 3.68 | -0.46 | 2 |
| Ach | -0.26 | -0.19 | -0.08 | 13.21 | 2.72 | -0.05 | 2 |
| Ach | -0.27 | -0.19 | -0.08 | 12.82 | 1.89 | -0.57 | 2 |
| Ach | -0.26 | -0.18 | -0.08 | 12.29 | 3.29 | 0.45 | 2 |
| Ach | -0.26 | -0.18 | -0.09 | 12.71 | 2.47 | 0.54 | 2 |
| Ach | -0.25 | -0.19 | -0.07 | 14.61 | 3.51 | -1.16 | 2 |
| 5-HT | -0.35 | -0.22 | -0.14 | 4.89 | -7.91 | 0.33 | 1 |
| 5-HT | -0.33 | -0.20 | -0.12 | 5.05 | -5.21 | 0.32 | 1 |
| 5-HT | -0.33 | -0.19 | -0.10 | 4.03 | -5.00 | -0.33 | 1 |
| 5-HT | -0.34 | -0.18 | -0.11 | 2.62 | -5.24 | 0.31 | 1 |
| 5-HT | -0.33 | -0.19 | -0.12 | 5.25 | -4.69 | 1.26 | 1 |
| 5-HT | -0.33 | -0.19 | -0.11 | 5.14 | -4.49 | 0.55 | 1 |

**Table S7**. Training matrix of the colorimetric response patterns obtained from an array of nanozymes against 6 neurotransmitters at 0.1 μM. LDA was carried out and resulting in 3 factors of the canonical scores.

|  | Colorimetric response patterns | | | LDA canonical scores | | | |
| --- | --- | --- | --- | --- | --- | --- | --- |
|  | ZnTCPP-Zn | ZnTCPP-Ti | ZnTCPP-Al | Factor 1 | Factor 2 | Factor 3 | Group |
| DA | -0.11 | 0.10 | -0.04 | 8.64 | -1.06 | -2.34 | 3 |
| DA | -0.09 | 0.10 | 0.00 | 9.99 | 0.28 | -3.46 | 3 |
| DA | -0.09 | 0.12 | 0.01 | 10.86 | -0.67 | -3.93 | 3 |
| DA | -0.08 | 0.13 | -0.02 | 11.73 | -1.42 | -3.09 | 3 |
| DA | -0.09 | 0.12 | 0.01 | 10.94 | -1.11 | -3.99 | 3 |
| DA | -0.09 | 0.12 | 0.02 | 10.62 | -1.70 | -4.56 | 3 |
| EP | -0.17 | 0.12 | -0.23 | 5.57 | -7.50 | 4.27 | 4 |
| EP | -0.16 | 0.12 | -0.20 | 6.38 | -6.42 | 3.20 | 4 |
| EP | -0.14 | 0.15 | -0.25 | 8.65 | -7.66 | 4.94 | 4 |
| EP | -0.15 | 0.14 | -0.22 | 7.84 | -7.94 | 3.94 | 4 |
| EP | -0.14 | 0.14 | -0.18 | 8.10 | -6.70 | 2.29 | 4 |
| EP | -0.14 | 0.14 | -0.23 | 7.94 | -7.21 | 4.17 | 4 |
| NE | -0.14 | -0.07 | -0.12 | -1.11 | 10.22 | 2.41 | 6 |
| NE | -0.11 | -0.07 | -0.10 | 0.48 | 12.60 | 2.04 | 6 |
| NE | -0.11 | -0.04 | -0.12 | 1.67 | 10.06 | 2.50 | 6 |
| NE | -0.11 | -0.04 | -0.12 | 1.97 | 11.07 | 2.44 | 6 |
| NE | -0.11 | -0.05 | -0.12 | 1.46 | 11.39 | 2.47 | 6 |
| NE | -0.10 | -0.05 | -0.12 | 2.24 | 12.09 | 2.84 | 6 |
| HA | -0.19 | -0.02 | -0.01 | -1.30 | 1.83 | -3.31 | 5 |
| HA | -0.18 | -0.03 | -0.02 | -1.28 | 4.20 | -2.48 | 5 |
| HA | -0.17 | -0.02 | -0.06 | -0.63 | 3.78 | -0.90 | 5 |
| HA | -0.17 | 0.01 | -0.05 | 1.43 | 1.29 | -1.67 | 5 |
| HA | -0.17 | 0.00 | -0.04 | 0.46 | 1.91 | -2.31 | 5 |
| HA | -0.18 | -0.01 | -0.05 | -0.37 | 2.42 | -1.56 | 5 |
| Ach | -0.31 | -0.05 | -0.09 | -9.15 | -5.55 | -1.90 | 2 |
| Ach | -0.28 | -0.03 | -0.13 | -7.19 | -4.41 | 0.33 | 2 |
| Ach | -0.27 | -0.05 | -0.11 | -7.40 | -2.09 | -0.47 | 2 |
| Ach | -0.27 | -0.05 | -0.13 | -7.64 | -2.34 | 0.29 | 2 |
| Ach | -0.28 | -0.06 | -0.16 | -8.25 | -1.96 | 1.60 | 2 |
| Ach | -0.27 | -0.05 | -0.06 | -6.91 | -2.19 | -2.32 | 2 |
| 5-HT | -0.31 | -0.08 | -0.15 | -11.30 | -3.41 | 1.02 | 1 |
| 5-HT | -0.31 | -0.08 | -0.15 | -11.30 | -3.41 | 1.02 | 1 |
| 5-HT | -0.30 | -0.09 | -0.12 | -11.00 | -2.01 | -0.02 | 1 |
| 5-HT | -0.31 | -0.09 | -0.06 | -11.18 | -2.09 | -2.35 | 1 |
| 5-HT | -0.30 | -0.09 | -0.11 | -10.44 | -1.76 | -0.42 | 1 |
| 5-HT | -0.30 | -0.08 | -0.11 | -10.53 | -2.50 | -0.66 | 1 |

**Table S8**. LDA jackknifed classification matrix table obtained from an array of nanozymes against 6 neurotransmitters at 1 μM. The jackknifed classification matrix with cross-validation reveals a 100% accuracy.

|  | 5-HT | Ach | DA | EP | HA | NE | %correct |
| --- | --- | --- | --- | --- | --- | --- | --- |
| 5-HT | 6 | 0 | 0 | 0 | 0 | 0 | 100 |
| Ach | 0 | 6 | 0 | 0 | 0 | 0 | 100 |
| DA | 0 | 0 | 6 | 0 | 0 | 0 | 100 |
| EP | 0 | 0 | 0 | 6 | 0 | 0 | 100 |
| HA | 0 | 0 | 0 | 0 | 6 | 0 | 100 |
| NE | 0 | 0 | 0 | 0 | 0 | 6 | 100 |
| Total | 6 | 6 | 6 | 6 | 6 | 6 | 100 |

**Table S9**. Detection and identification of unknown samples at 1 μM using LDA from the array of nanozymes. According to the verification, 24 among 24 unknown samples were correctly identified, representing an accuracy of 100%.

| Sample | Colorimetric response patterns | | | Results LDA | | | Analyte | |
| --- | --- | --- | --- | --- | --- | --- | --- | --- |
| # | ZnTCPP-Zn | ZnTCPP-Ti | ZnTCPP-Al | Factor 1 | Factor 2 | Factor 3 | Identification | Verification |
| 1 | -0.31 | -0.14 | -0.04 | -0.62 | -1.11 | -2.40 | 3 | 3 |
| 2 | -0.30 | -0.15 | -0.03 | 0.78 | -0.32 | -2.60 | 3 | 3 |
| 3 | -0.31 | -0.15 | -0.04 | 0.70 | -1.02 | -1.92 | 3 | 3 |
| 4 | -0.30 | -0.14 | -0.04 | 1.18 | 0.15 | -2.08 | 3 | 3 |
| 5 | -0.29 | -0.16 | -0.05 | 4.73 | 0.68 | -1.18 | 4 | 4 |
| 6 | -0.28 | -0.15 | -0.06 | 5.73 | 1.76 | -0.10 | 4 | 4 |
| 7 | -0.28 | -0.15 | -0.08 | 7.36 | 1.97 | 1.57 | 4 | 4 |
| 8 | -0.31 | -0.17 | -0.08 | 3.47 | -2.14 | -0.25 | 3 | 3 |
| 9 | -0.28 | -0.12 | -0.04 | 1.26 | 2.53 | 0.41 | 6 | 6 |
| 10 | -0.28 | -0.11 | -0.04 | 1.15 | 3.10 | 0.61 | 6 | 6 |
| 11 | -0.28 | -0.11 | -0.03 | -0.17 | 2.83 | -0.32 | 6 | 6 |
| 12 | -0.28 | -0.09 | -0.03 | -2.40 | 3.13 | 1.07 | 6 | 6 |
| 13 | -0.33 | 0.02 | 0.05 | -24.57 | 1.63 | 0.74 | 5 | 5 |
| 14 | -0.34 | 0.04 | 0.05 | -27.41 | 1.32 | 0.66 | 5 | 5 |
| 15 | -0.35 | 0.03 | 0.06 | -28.51 | 0.26 | -0.67 | 5 | 5 |
| 16 | -0.34 | 0.03 | 0.07 | -26.91 | 1.01 | -1.29 | 5 | 5 |
| 17 | -0.28 | -0.17 | -0.11 | 10.12 | 1.04 | 2.79 | 4 | 4 |
| 18 | -0.25 | -0.19 | -0.08 | 14.62 | 3.70 | 0.20 | 2 | 2 |
| 19 | -0.25 | -0.19 | -0.07 | 14.41 | 3.74 | -0.29 | 2 | 2 |
| 20 | -0.26 | -0.18 | -0.07 | 12.69 | 3.38 | 0.07 | 2 | 2 |
| 21 | -0.34 | -0.19 | -0.12 | 2.83 | -5.64 | 0.81 | 1 | 1 |
| 22 | -0.33 | -0.20 | -0.11 | 5.14 | -5.05 | 0.11 | 1 | 1 |
| 23 | -0.34 | -0.20 | -0.11 | 3.44 | -5.96 | 0.02 | 1 | 1 |
| 24 | -0.34 | -0.20 | -0.11 | 3.74 | -6.03 | -0.14 | 1 | 1 |

**Table S10**. LDA jackknifed classification matrix table obtained from an array of nanozymes against 6 neurotransmitters at 0.1 μM. The jackknifed classification matrix with cross-validation reveals a 100% accuracy.

|  | 5-HT | Ach | DA | EP | HA | NE | %correct |
| --- | --- | --- | --- | --- | --- | --- | --- |
| 5-HT | 6 | 0 | 0 | 0 | 0 | 0 | 100 |
| Ach | 0 | 6 | 0 | 0 | 0 | 0 | 100 |
| DA | 0 | 0 | 6 | 0 | 0 | 0 | 100 |
| EP | 0 | 0 | 0 | 6 | 0 | 0 | 100 |
| HA | 0 | 0 | 0 | 0 | 6 | 0 | 100 |
| NE | 0 | 0 | 0 | 0 | 0 | 6 | 100 |
| Total | 6 | 6 | 6 | 6 | 6 | 6 | 100 |

**Table S11**. Detection and identification of unknown samples at 0.1 μM using LDA from the array of nanozymes. According to the verification, 24 among 24 unknown samples were correctly identified, representing an accuracy of 100%.

| Sample | Colorimetric response patterns | | | Results LDA | | | Analyte | |
| --- | --- | --- | --- | --- | --- | --- | --- | --- |
| # | ZnTCPP-Zn | ZnTCPP-Ti | ZnTCPP-Al | Factor 1 | Factor 2 | Factor 3 | Identification | Verification |
| 1 | -0.08 | 0.12 | 0.00 | 11.44 | -0.83 | -3.58 | 3 | 3 |
| 2 | -0.08 | 0.11 | 0.01 | 10.66 | 0.66 | -4.07 | 3 | 3 |
| 3 | -0.09 | 0.11 | -0.15 | 9.72 | 0.22 | 2.30 | 3 | 3 |
| 4 | -0.10 | 0.11 | -0.12 | 9.62 | -0.70 | 0.99 | 3 | 3 |
| 5 | -0.15 | 0.15 | -0.19 | 8.44 | -8.91 | 2.54 | 4 | 4 |
| 6 | -0.13 | 0.15 | -0.19 | 9.10 | -6.74 | 2.91 | 4 | 4 |
| 7 | -0.14 | 0.14 | -0.20 | 8.36 | -7.27 | 3.14 | 4 | 4 |
| 8 | -0.15 | 0.14 | -0.20 | 7.83 | -7.53 | 3.19 | 4 | 4 |
| 9 | -0.10 | -0.05 | -0.17 | 2.02 | 12.02 | 4.55 | 6 | 6 |
| 10 | -0.10 | -0.06 | -0.16 | 1.19 | 13.54 | 4.64 | 6 | 6 |
| 11 | -0.10 | -0.04 | -0.15 | 2.14 | 11.55 | 3.91 | 6 | 6 |
| 12 | -0.10 | -0.05 | -0.14 | 2.19 | 12.11 | 3.42 | 6 | 6 |
| 13 | -0.17 | -0.03 | -0.05 | -0.58 | 4.35 | -1.38 | 5 | 5 |
| 14 | -0.17 | 0.02 | -0.09 | 1.24 | 0.61 | -0.52 | 5 | 5 |
| 15 | -0.18 | -0.01 | 0.00 | 0.04 | 2.18 | -3.55 | 5 | 5 |
| 16 | -0.17 | -0.02 | -0.04 | -0.40 | 3.44 | -1.87 | 5 | 5 |
| 17 | -0.28 | -0.06 | -0.08 | -7.94 | -2.54 | -1.60 | 2 | 2 |
| 18 | -0.28 | -0.06 | -0.13 | -8.53 | -1.98 | 0.66 | 2 | 2 |
| 19 | -0.27 | -0.06 | -0.14 | -7.91 | -0.98 | 1.07 | 2 | 2 |
| 20 | -0.28 | -0.06 | -0.09 | -7.89 | -2.38 | -1.00 | 2 | 2 |
| 21 | -0.31 | -0.08 | -0.16 | -11.04 | -2.98 | 1.21 | 1 | 1 |
| 22 | -0.31 | -0.08 | -0.14 | -11.19 | -3.29 | 0.70 | 1 | 1 |
| 23 | -0.30 | -0.08 | -0.16 | -10.63 | -2.85 | 1.60 | 1 | 1 |
| 24 | -0.30 | -0.07 | -0.09 | -9.79 | -3.16 | -1.54 | 1 | 1 |

**Table S12**. Training matrix of colorimetric response patterns obtained from an array of nano-enzyme against DA with various concentrations. LDA was carried out and resulting in 3 actors of the canonical scores.

|  | Colorimetric response patterns | | | LDA canonical scores | | | |
| --- | --- | --- | --- | --- | --- | --- | --- |
|  | ZnTCPP-Zn | ZnTCPP-Ti | ZnTCPP-Al | Factor 1 | Factor 2 | Factor 3 | Group |
| 0.5 μM | 0.07 | -0.02 | -0.12 | 6.22 | 8.86 | 1.23 | 1 |
| 0.5 μM | 0.10 | -0.02 | -0.11 | 6.17 | 11.76 | 2.31 | 1 |
| 0.5 μM | 0.10 | -0.04 | -0.09 | 4.30 | 12.27 | 2.18 | 1 |
| 0.5 μM | 0.10 | -0.03 | -0.09 | 4.51 | 12.41 | 2.37 | 1 |
| 0.5 μM | 0.12 | 0.00 | -0.09 | 5.31 | 13.28 | 3.60 | 1 |
| 0.5 μM | 0.12 | -0.01 | -0.09 | 5.72 | 13.34 | 3.31 | 1 |
| 2 μM | -0.01 | -0.07 | -0.14 | 2.53 | 1.71 | -2.09 | 2 |
| 2 μM | 0.01 | -0.07 | -0.14 | 4.14 | 3.74 | -1.73 | 2 |
| 2 μM | 0.01 | -0.04 | -0.13 | 1.83 | 2.75 | 0.01 | 2 |
| 2 μM | 0.00 | -0.05 | -0.14 | 2.95 | 2.20 | -1.06 | 2 |
| 2 μM | 0.01 | -0.06 | -0.13 | 2.72 | 3.41 | -1.04 | 2 |
| 2 μM | 0.00 | -0.06 | -0.12 | 0.89 | 2.79 | -0.71 | 2 |
| 3.5 μM | -0.11 | -0.21 | -0.14 | -6.63 | -5.21 | -7.61 | 3 |
| 3.5 μM | -0.09 | -0.20 | -0.13 | -6.39 | -3.93 | -6.89 | 3 |
| 3.5 μM | -0.10 | -0.20 | -0.13 | -6.10 | -4.29 | -6.89 | 3 |
| 3.5 μM | -0.09 | -0.19 | -0.12 | -7.28 | -3.55 | -6.14 | 3 |
| 3.5 μM | -0.08 | -0.16 | -0.12 | -6.31 | -3.70 | -5.25 | 3 |
| 3.5 μM | -0.08 | -0.18 | -0.12 | -6.87 | -3.28 | -5.77 | 3 |
| 5 μM | 0.00 | -0.16 | -0.14 | 2.61 | 4.61 | -5.23 | 4 |
| 5 μM | 0.01 | -0.17 | -0.15 | 3.50 | 5.48 | -5.61 | 4 |
| 5 μM | 0.02 | -0.14 | -0.15 | 4.46 | 5.65 | -4.23 | 4 |
| 5 μM | 0.02 | -0.14 | -0.14 | 4.02 | 5.68 | -4.10 | 4 |
| 5 μM | 0.01 | -0.17 | -0.14 | 3.10 | 5.42 | -5.32 | 4 |
| 5 μM | 0.01 | -0.16 | -0.15 | 4.53 | 5.96 | -5.35 | 4 |
| 6.5 μM | -0.03 | -0.06 | 0.05 | -23.08 | 0.82 | 5.33 | 5 |
| 6.5 μM | -0.02 | -0.09 | 0.07 | -24.17 | 2.81 | 4.83 | 5 |
| 6.5 μM | -0.03 | -0.07 | 0.08 | -25.84 | 1.71 | 5.96 | 5 |
| 6.5 μM | -0.03 | -0.06 | 0.07 | -25.29 | 1.08 | 5.82 | 5 |
| 6.5 μM | -0.02 | -0.09 | 0.07 | -23.96 | 2.95 | 5.01 | 5 |
| 6.5 μM | -0.03 | -0.09 | 0.07 | -25.19 | 1.30 | 4.86 | 5 |
| 8 μM | -0.12 | -0.01 | -0.14 | -6.30 | -10.92 | -0.64 | 6 |
| 8 μM | -0.14 | -0.01 | -0.12 | -10.14 | -12.38 | -0.02 | 6 |
| 8 μM | -0.12 | 0.13 | -0.12 | -7.07 | -13.82 | 4.88 | 6 |
| 8 μM | -0.12 | 0.00 | -0.12 | -9.06 | -11.06 | 0.28 | 6 |
| 8 μM | -0.12 | -0.01 | -0.12 | -8.92 | -10.73 | 0.14 | 6 |
| 8 μM | -0.13 | 0.02 | -0.11 | -9.48 | -11.85 | 1.35 | 6 |
| 9.5 μM | 0.02 | 0.29 | -0.32 | 28.75 | -5.10 | 4.56 | 7 |
| 9.5 μM | 0.00 | 0.28 | -0.34 | 29.17 | -6.58 | 3.40 | 7 |
| 9.5 μM | -0.01 | 0.28 | -0.33 | 28.21 | -7.26 | 3.46 | 7 |
| 9.5 μM | 0.01 | 0.27 | -0.32 | 27.80 | -5.05 | 3.84 | 7 |
| 9.5 μM | 0.00 | 0.28 | -0.32 | 26.17 | -6.77 | 3.97 | 7 |
| 9.5 μM | 0.00 | 0.27 | -0.33 | 28.46 | -6.50 | 3.02 | 7 |

**Table S13**. Training matrix of colorimetric response patterns obtained from an array of nano-enzyme against EP with various concentrations. LDA was carried out and resulting in 3 actors of the canonical scores.

|  | Colorimetric response patterns | | | LDA canonical scores | | | |
| --- | --- | --- | --- | --- | --- | --- | --- |
|  | ZnTCPP-Zn | ZnTCPP-Ti | ZnTCPP-Al | Factor 1 | Factor 2 | Factor 3 | Group |
| 0.5 μM | 0.04 | -0.10 | -0.14 | 30.37 | -3.27 | 1.69 | 1 |
| 0.5 μM | 0.08 | -0.06 | -0.17 | 33.43 | -4.05 | 4.91 | 1 |
| 0.5 μM | 0.08 | -0.06 | -0.14 | 34.32 | -3.31 | 3.07 | 1 |
| 0.5 μM | 0.07 | -0.05 | -0.15 | 33.76 | -5.53 | 4.05 | 1 |
| 0.5 μM | 0.09 | -0.05 | -0.16 | 34.69 | -4.60 | 4.97 | 1 |
| 0.5 μM | 0.09 | -0.05 | -0.16 | 34.76 | -3.20 | 4.81 | 1 |
| 2 μM | -0.16 | -0.13 | -0.10 | 15.14 | -17.85 | -2.82 | 2 |
| 2 μM | -0.12 | -0.13 | -0.08 | 18.55 | -14.20 | -3.64 | 2 |
| 2 μM | -0.13 | -0.14 | -0.12 | 16.94 | -14.53 | -2.01 | 2 |
| 2 μM | -0.13 | -0.14 | -0.14 | 16.47 | -14.43 | -0.83 | 2 |
| 2 μM | -0.13 | -0.14 | -0.10 | 17.49 | -14.42 | -3.01 | 2 |
| 2 μM | -0.13 | -0.12 | -0.11 | 17.65 | -16.35 | -1.83 | 2 |
| 3.5 μM | 0.00 | -0.29 | -0.21 | 21.43 | 16.11 | -2.97 | 3 |
| 3.5 μM | 0.01 | -0.29 | -0.21 | 22.93 | 17.86 | -3.05 | 3 |
| 3.5 μM | 0.01 | -0.28 | -0.21 | 22.79 | 16.48 | -2.73 | 3 |
| 3.5 μM | 0.02 | -0.28 | -0.21 | 23.81 | 17.65 | -2.84 | 3 |
| 3.5 μM | 0.01 | -0.28 | -0.21 | 23.20 | 16.38 | -2.54 | 3 |
| 3.5 μM | 0.03 | -0.28 | -0.20 | 24.19 | 18.02 | -2.99 | 3 |
| 5 μM | -0.27 | -0.35 | -0.35 | -3.23 | -1.39 | 0.11 | 4 |
| 5 μM | -0.27 | -0.36 | -0.36 | -3.84 | -1.25 | 0.42 | 4 |
| 5 μM | -0.27 | -0.36 | -0.36 | -3.62 | -1.07 | 0.26 | 4 |
| 5 μM | -0.26 | -0.36 | -0.37 | -2.96 | -0.06 | 0.65 | 4 |
| 5 μM | -0.27 | -0.35 | -0.35 | -3.23 | -1.39 | 0.11 | 4 |
| 5 μM | -0.27 | -0.36 | -0.37 | -3.81 | -0.32 | 0.78 | 4 |
| 6.5 μM | -0.32 | -0.42 | -0.32 | -8.12 | 1.96 | -4.55 | 5 |
| 6.5 μM | -0.32 | -0.43 | -0.39 | -9.54 | 3.45 | -1.34 | 5 |
| 6.5 μM | -0.32 | -0.43 | -0.40 | -9.51 | 2.97 | -1.00 | 5 |
| 6.5 μM | -0.31 | -0.42 | -0.40 | -9.11 | 2.87 | -0.81 | 5 |
| 6.5 μM | -0.31 | -0.43 | -0.41 | -9.48 | 3.90 | -0.64 | 5 |
| 6.5 μM | -0.32 | -0.43 | -0.40 | -9.60 | 3.47 | -1.17 | 5 |
| 8 μM | -0.44 | -0.52 | -0.64 | -26.11 | 2.50 | 6.45 | 6 |
| 8 μM | -0.46 | -0.54 | -0.63 | -27.29 | 3.26 | 5.37 | 6 |
| 8 μM | -0.44 | -0.52 | -0.63 | -25.82 | 3.37 | 6.13 | 6 |
| 8 μM | -0.45 | -0.53 | -0.64 | -26.58 | 3.32 | 6.43 | 6 |
| 8 μM | -0.45 | -0.54 | -0.64 | -27.08 | 3.91 | 6.07 | 6 |
| 8 μM | -0.45 | -0.54 | -0.64 | -27.01 | 4.60 | 5.91 | 6 |
| 9.5 μM | -0.56 | -0.56 | -0.47 | -32.64 | -4.28 | -4.08 | 7 |
| 9.5 μM | -0.57 | -0.59 | -0.46 | -34.17 | -2.24 | -5.84 | 7 |
| 9.5 μM | -0.57 | -0.58 | -0.53 | -35.70 | -3.31 | -1.98 | 7 |
| 9.5 μM | -0.56 | -0.56 | -0.50 | -34.02 | -4.42 | -2.60 | 7 |
| 9.5 μM | -0.57 | -0.56 | -0.48 | -34.03 | -5.11 | -3.81 | 7 |
| 9.5 μM | -0.57 | -0.59 | -0.52 | -35.39 | -1.50 | -3.14 | 7 |

**Table S14**. Training matrix of colorimetric response patterns obtained from an array of nano-enzyme against 5-HT with various concentrations. LDA was carried out and resulting in 3 actors of the canonical scores.

|  | Colorimetric response patterns | | | LDA canonical scores | | | |
| --- | --- | --- | --- | --- | --- | --- | --- |
|  | ZnTCPP-Zn | ZnTCPP-Ti | ZnTCPP-Al | Factor 1 | Factor 2 | Factor 3 | Group |
| 0.5 μM | -0.08 | -0.24 | -0.01 | 19.91 | 8.74 | -0.76 | 1 |
| 0.5 μM | -0.06 | -0.22 | 0.02 | 23.63 | 9.19 | -1.95 | 1 |
| 0.5 μM | -0.07 | -0.22 | 0.00 | 22.93 | 8.12 | -0.85 | 1 |
| 0.5 μM | -0.07 | -0.22 | 0.00 | 23.36 | 7.50 | -0.79 | 1 |
| 0.5 μM | -0.07 | -0.22 | 0.01 | 22.69 | 7.86 | -1.68 | 1 |
| 0.5 μM | -0.06 | -0.22 | 0.01 | 24.36 | 8.31 | -1.15 | 1 |
| 2 μM | -0.10 | -0.29 | -0.01 | 13.72 | 13.60 | -2.48 | 2 |
| 2 μM | -0.09 | -0.28 | -0.02 | 16.02 | 12.99 | -0.57 | 2 |
| 2 μM | -0.10 | -0.28 | -0.04 | 13.69 | 12.17 | -0.49 | 2 |
| 2 μM | -0.08 | -0.28 | -0.03 | 15.20 | 14.19 | 0.33 | 2 |
| 2 μM | -0.10 | -0.28 | -0.04 | 13.49 | 11.87 | -0.32 | 2 |
| 2 μM | -0.10 | -0.28 | -0.03 | 14.22 | 12.90 | -0.41 | 2 |
| 3.5 μM | -0.19 | -0.14 | -0.12 | 19.86 | -19.54 | 4.15 | 3 |
| 3.5 μM | -0.19 | -0.14 | -0.09 | 19.37 | -19.32 | 0.98 | 3 |
| 3.5 μM | -0.20 | -0.14 | -0.10 | 19.08 | -19.79 | 1.74 | 3 |
| 3.5 μM | -0.18 | -0.14 | -0.09 | 21.31 | -17.92 | 1.85 | 3 |
| 3.5 μM | -0.18 | -0.14 | -0.11 | 19.83 | -17.71 | 3.55 | 3 |
| 3.5 μM | -0.19 | -0.14 | -0.12 | 19.41 | -20.10 | 3.49 | 3 |
| 5 μM | -0.28 | -0.30 | -0.12 | -3.85 | -6.41 | -4.04 | 4 |
| 5 μM | -0.27 | -0.31 | -0.11 | -3.34 | -4.46 | -4.37 | 4 |
| 5 μM | -0.28 | -0.31 | -0.10 | -3.53 | -5.25 | -5.30 | 4 |
| 5 μM | -0.28 | -0.31 | -0.10 | -4.20 | -4.89 | -6.19 | 4 |
| 5 μM | -0.28 | -0.31 | -0.13 | -4.29 | -5.31 | -3.00 | 4 |
| 5 μM | -0.27 | -0.32 | -0.12 | -4.48 | -4.31 | -3.52 | 4 |
| 6.5 μM | -0.24 | -0.34 | -0.16 | -4.48 | 1.89 | 1.21 | 5 |
| 6.5 μM | -0.24 | -0.34 | -0.16 | -4.48 | 1.89 | 1.21 | 5 |
| 6.5 μM | -0.24 | -0.34 | -0.19 | -4.74 | 1.26 | 4.28 | 5 |
| 6.5 μM | -0.25 | -0.34 | -0.16 | -4.80 | 0.85 | 1.17 | 5 |
| 6.5 μM | -0.24 | -0.34 | -0.17 | -4.98 | 1.12 | 2.15 | 5 |
| 6.5 μM | -0.24 | -0.34 | -0.17 | -4.37 | 1.90 | 2.93 | 5 |
| 8 μM | -0.30 | -0.45 | -0.25 | -21.37 | 8.93 | 4.75 | 6 |
| 8 μM | -0.31 | -0.45 | -0.27 | -22.20 | 5.94 | 5.03 | 6 |
| 8 μM | -0.32 | -0.45 | -0.27 | -23.00 | 6.04 | 5.02 | 6 |
| 8 μM | -0.30 | -0.45 | -0.24 | -21.74 | 8.54 | 3.50 | 6 |
| 8 μM | -0.32 | -0.45 | -0.25 | -22.36 | 5.85 | 3.60 | 6 |
| 8 μM | -0.32 | -0.45 | -0.25 | -23.03 | 6.21 | 2.71 | 6 |
| 9.5 μM | -0.39 | -0.40 | -0.23 | -23.23 | -7.48 | -2.48 | 7 |
| 9.5 μM | -0.40 | -0.42 | -0.23 | -27.14 | -6.19 | -3.30 | 7 |
| 9.5 μM | -0.39 | -0.43 | -0.24 | -26.90 | -3.54 | -2.26 | 7 |
| 9.5 μM | -0.40 | -0.42 | -0.23 | -26.19 | -5.47 | -3.37 | 7 |
| 9.5 μM | -0.40 | -0.42 | -0.24 | -26.10 | -6.03 | -2.46 | 7 |
| 9.5 μM | -0.39 | -0.43 | -0.24 | -27.31 | -4.14 | -1.92 | 7 |

**Table S15**. Training matrix of colorimetric response patterns obtained from an array of nano-enzyme against NE with various concentrations. LDA was carried out and resulting in 3 actors of the canonical scores.

|  | Colorimetric response patterns | | | LDA canonical scores | | | |
| --- | --- | --- | --- | --- | --- | --- | --- |
|  | ZnTCPP-Zn | ZnTCPP-Ti | ZnTCPP-Al | Factor 1 | Factor 2 | Factor 3 | Group |
| 0.5 μM | -0.12 | -0.24 | -0.26 | 13.98 | -7.71 | 0.17 | 1 |
| 0.5 μM | -0.09 | -0.23 | -0.26 | 13.29 | -5.83 | 0.70 | 1 |
| 0.5 μM | -0.08 | -0.22 | -0.22 | 12.96 | -5.13 | -0.51 | 1 |
| 0.5 μM | -0.08 | -0.21 | -0.28 | 11.07 | -4.91 | 2.04 | 1 |
| 0.5 μM | -0.08 | -0.22 | -0.30 | 12.34 | -5.30 | 2.58 | 1 |
| 0.5 μM | -0.07 | -0.20 | -0.29 | 10.92 | -4.34 | 2.60 | 1 |
| 2 μM | 0.03 | -0.26 | -0.23 | 23.80 | 1.48 | -0.17 | 2 |
| 2 μM | 0.06 | -0.26 | -0.21 | 25.32 | 3.14 | -0.62 | 2 |
| 2 μM | 0.07 | -0.24 | -0.23 | 23.67 | 3.34 | 0.28 | 2 |
| 2 μM | 0.07 | -0.25 | -0.19 | 24.88 | 3.85 | -1.00 | 2 |
| 2 μM | 0.08 | -0.25 | -0.17 | 25.31 | 4.20 | -1.79 | 2 |
| 2 μM | 0.08 | -0.23 | -0.15 | 23.83 | 4.35 | -2.35 | 2 |
| 3.5 μM | -0.03 | -0.11 | -0.22 | 0.20 | -1.87 | 2.09 | 3 |
| 3.5 μM | -0.01 | -0.10 | -0.20 | 0.38 | -0.42 | 1.79 | 3 |
| 3.5 μM | -0.02 | -0.10 | -0.23 | -0.77 | -0.91 | 2.86 | 3 |
| 3.5 μM | 0.00 | -0.11 | -0.22 | 1.25 | 0.06 | 2.59 | 3 |
| 3.5 μM | 0.03 | -0.09 | -0.21 | 0.72 | 2.01 | 2.50 | 3 |
| 3.5 μM | 0.02 | -0.09 | -0.22 | -0.74 | 1.10 | 3.11 | 3 |
| 5 μM | -0.08 | -0.13 | -0.22 | 0.94 | -4.36 | 1.42 | 4 |
| 5 μM | -0.07 | -0.13 | -0.17 | 2.05 | -4.03 | -0.29 | 4 |
| 5 μM | -0.06 | -0.13 | -0.17 | 1.71 | -3.13 | -0.24 | 4 |
| 5 μM | -0.07 | -0.11 | -0.16 | -0.19 | -3.40 | -0.43 | 4 |
| 5 μM | -0.07 | -0.13 | -0.18 | 1.23 | -3.58 | 0.34 | 4 |
| 5 μM | -0.06 | -0.12 | -0.16 | 0.99 | -3.33 | -0.38 | 4 |
| 6.5 μM | 0.13 | 0.04 | -0.05 | -10.56 | 8.64 | -0.01 | 5 |
| 6.5 μM | 0.15 | 0.06 | -0.07 | -12.57 | 9.81 | 1.36 | 5 |
| 6.5 μM | 0.18 | 0.05 | -0.07 | -10.22 | 11.85 | 1.49 | 5 |
| 6.5 μM | 0.13 | 0.04 | -0.07 | -11.54 | 8.95 | 0.81 | 5 |
| 6.5 μM | 0.15 | 0.05 | -0.03 | -11.61 | 9.98 | -0.16 | 5 |
| 6.5 μM | 0.17 | 0.04 | -0.06 | -9.55 | 11.18 | 0.63 | 5 |
| 8 μM | -0.04 | -0.09 | -0.05 | -0.82 | -1.49 | -3.75 | 6 |
| 8 μM | -0.01 | -0.09 | -0.05 | 1.02 | 0.60 | -3.74 | 6 |
| 8 μM | -0.02 | -0.09 | -0.09 | -0.52 | -0.21 | -2.08 | 6 |
| 8 μM | -0.03 | -0.10 | -0.11 | -0.28 | -1.17 | -1.73 | 6 |
| 8 μM | -0.03 | -0.08 | -0.01 | -1.39 | -0.41 | -4.83 | 6 |
| 8 μM | -0.02 | -0.10 | -0.09 | 0.30 | -0.60 | -2.29 | 6 |
| 9.5 μM | -0.09 | 0.09 | 0.01 | -28.68 | -3.46 | -2.58 | 7 |
| 9.5 μM | -0.10 | 0.07 | -0.09 | -26.65 | -4.42 | 0.76 | 7 |
| 9.5 μM | -0.10 | 0.06 | -0.09 | -25.42 | -4.32 | 0.60 | 7 |
| 9.5 μM | -0.09 | 0.09 | -0.03 | -27.89 | -3.58 | -1.10 | 7 |
| 9.5 μM | -0.08 | 0.07 | -0.07 | -25.49 | -3.22 | 0.05 | 7 |
| 9.5 μM | -0.09 | 0.08 | -0.04 | -27.22 | -3.41 | -0.75 | 7 |

**Table S16**. Training matrix of colorimetric response patterns obtained from an array of nano-enzyme against HA with various concentrations. LDA was carried out and resulting in 3 actors of the canonical scores.

|  | Colorimetric response patterns | | | LDA canonical scores | | | |
| --- | --- | --- | --- | --- | --- | --- | --- |
|  | ZnTCPP-Zn | ZnTCPP-Ti | ZnTCPP-Al | Factor 1 | Factor 2 | Factor 3 | Group |
| 0.5 μM | -0.11 | 0.18 | -0.05 | -10.31 | -2.08 | 0.32 | 1 |
| 0.5 μM | -0.09 | 0.21 | -0.04 | -11.92 | -0.70 | 2.10 | 1 |
| 0.5 μM | -0.08 | 0.22 | -0.02 | -11.46 | -0.49 | 3.06 | 1 |
| 0.5 μM | -0.08 | 0.23 | -0.02 | -11.94 | -0.26 | 3.57 | 1 |
| 0.5 μM | -0.07 | 0.24 | -0.04 | -13.18 | 1.14 | 3.61 | 1 |
| 0.5 μM | -0.08 | 0.23 | -0.02 | -12.93 | -0.04 | 3.56 | 1 |
| 2 μM | -0.12 | 0.28 | -0.02 | -20.61 | -2.68 | 2.47 | 2 |
| 2 μM | -0.11 | 0.28 | -0.07 | -20.53 | -0.09 | 1.53 | 2 |
| 2 μM | -0.10 | 0.27 | -0.06 | -18.19 | 0.17 | 2.31 | 2 |
| 2 μM | -0.10 | 0.27 | -0.02 | -17.51 | -1.13 | 3.27 | 2 |
| 2 μM | -0.07 | 0.27 | -0.04 | -16.49 | 1.35 | 3.85 | 2 |
| 2 μM | -0.09 | 0.28 | -0.02 | -18.55 | -0.71 | 3.70 | 2 |
| 3.5 μM | -0.06 | 0.15 | -0.16 | -5.60 | 5.04 | -0.92 | 3 |
| 3.5 μM | -0.05 | 0.14 | -0.14 | -3.87 | 5.69 | 0.23 | 3 |
| 3.5 μM | -0.04 | 0.14 | -0.14 | -3.73 | 5.86 | 0.33 | 3 |
| 3.5 μM | -0.03 | 0.16 | -0.14 | -4.68 | 6.65 | 1.31 | 3 |
| 3.5 μM | -0.03 | 0.16 | -0.11 | -4.42 | 5.71 | 2.30 | 3 |
| 3.5 μM | -0.04 | 0.16 | -0.11 | -4.80 | 5.54 | 1.93 | 3 |
| 5 μM | -0.15 | 0.10 | -0.06 | -4.53 | -5.68 | -3.57 | 4 |
| 5 μM | -0.15 | 0.10 | -0.01 | -2.93 | -6.47 | -1.77 | 4 |
| 5 μM | -0.15 | 0.08 | -0.04 | -2.31 | -6.44 | -3.25 | 4 |
| 5 μM | -0.14 | 0.09 | -0.03 | -2.27 | -5.88 | -2.26 | 4 |
| 5 μM | -0.15 | 0.10 | -0.02 | -3.00 | -6.51 | -2.36 | 4 |
| 5 μM | -0.15 | 0.09 | -0.04 | -2.75 | -6.41 | -3.18 | 4 |
| 6.5 μM | -0.13 | 0.20 | -0.14 | -15.09 | -0.75 | -3.28 | 5 |
| 6.5 μM | -0.13 | 0.19 | -0.21 | -15.89 | 1.99 | -5.16 | 5 |
| 6.5 μM | -0.12 | 0.20 | -0.18 | -15.65 | 1.57 | -3.78 | 5 |
| 6.5 μM | -0.12 | 0.20 | -0.17 | -14.85 | 1.39 | -3.44 | 5 |
| 6.5 μM | -0.14 | 0.19 | -0.17 | -15.38 | -0.14 | -4.22 | 5 |
| 6.5 μM | -0.13 | 0.20 | -0.19 | -16.13 | 1.04 | -4.69 | 5 |
| 8 μM | -0.04 | -0.13 | -0.08 | 26.18 | 2.16 | -2.37 | 6 |
| 8 μM | -0.02 | -0.11 | -0.09 | 25.85 | 4.07 | -1.49 | 6 |
| 8 μM | -0.04 | -0.11 | -0.12 | 24.45 | 3.88 | -2.90 | 6 |
| 8 μM | -0.03 | -0.11 | -0.09 | 25.18 | 3.24 | -2.03 | 6 |
| 8 μM | -0.04 | -0.12 | -0.11 | 25.29 | 3.16 | -2.95 | 6 |
| 8 μM | -0.03 | -0.12 | -0.11 | 25.67 | 4.19 | -2.23 | 6 |
| 9.5 μM | -0.03 | -0.09 | 0.09 | 26.99 | -2.11 | 3.50 | 7 |
| 9.5 μM | -0.04 | -0.09 | 0.11 | 26.48 | -3.39 | 3.74 | 7 |
| 9.5 μM | -0.04 | -0.13 | 0.03 | 28.63 | -1.56 | 0.87 | 7 |
| 9.5 μM | -0.04 | -0.11 | 0.07 | 28.41 | -2.27 | 2.36 | 7 |
| 9.5 μM | -0.05 | -0.11 | 0.15 | 28.62 | -5.47 | 4.24 | 7 |
| 9.5 μM | -0.04 | -0.13 | 0.07 | 29.77 | -2.56 | 1.71 | 7 |

**Table S17**. Training matrix of colorimetric response patterns obtained from an array of nano-enzyme against ACh with various concentrations. LDA was carried out and resulting in 3 actors of the canonical scores.

|  | Colorimetric response patterns | | | LDA canonical scores | | | |
| --- | --- | --- | --- | --- | --- | --- | --- |
|  | ZnTCPP-Zn | ZnTCPP-Ti | ZnTCPP-Al | Factor 1 | Factor 2 | Factor 3 | Group |
| 0.5 μM | 0.27 | 0.09 | 0.02 | 26.40 | -0.10 | 3.28 | 1 |
| 0.5 μM | 0.27 | 0.09 | 0.01 | 25.18 | 0.44 | 2.99 | 1 |
| 0.5 μM | 0.26 | 0.10 | 0.04 | 24.93 | -1.26 | 1.43 | 1 |
| 0.5 μM | 0.27 | 0.09 | -0.04 | 24.51 | 2.73 | 4.35 | 1 |
| 0.5 μM | 0.27 | 0.09 | 0.01 | 25.34 | 0.16 | 2.85 | 1 |
| 0.5 μM | 0.28 | 0.12 | 0.00 | 26.02 | 1.84 | 1.87 | 1 |
| 2 μM | 0.03 | 0.03 | -0.03 | -3.07 | -6.06 | -0.28 | 2 |
| 2 μM | 0.07 | 0.06 | -0.04 | 0.60 | -3.27 | -0.48 | 2 |
| 2 μM | 0.06 | 0.04 | -0.04 | -0.27 | -4.50 | 0.32 | 2 |
| 2 μM | 0.06 | 0.05 | -0.04 | 0.21 | -3.84 | -0.48 | 2 |
| 2 μM | 0.06 | 0.05 | -0.06 | -0.67 | -3.00 | 0.36 | 2 |
| 2 μM | 0.06 | 0.04 | -0.03 | 0.18 | -4.71 | 0.26 | 2 |
| 3.5 μM | 0.10 | 0.06 | -0.11 | 2.03 | 0.99 | 2.50 | 3 |
| 3.5 μM | 0.13 | 0.06 | -0.11 | 5.46 | 1.89 | 3.22 | 3 |
| 3.5 μM | 0.12 | 0.08 | -0.12 | 3.60 | 2.67 | 2.11 | 3 |
| 3.5 μM | 0.12 | 0.07 | -0.09 | 4.77 | 0.41 | 1.72 | 3 |
| 3.5 μM | 0.11 | 0.05 | -0.12 | 3.64 | 1.45 | 3.50 | 3 |
| 3.5 μM | 0.13 | 0.07 | -0.09 | 6.60 | 1.28 | 2.16 | 3 |
| 5 μM | -0.07 | 0.09 | -0.34 | -24.73 | 9.46 | 0.42 | 4 |
| 5 μM | -0.07 | 0.09 | -0.38 | -25.70 | 11.69 | 1.70 | 4 |
| 5 μM | -0.07 | 0.11 | -0.37 | -25.65 | 11.53 | -0.13 | 4 |
| 5 μM | -0.07 | 0.10 | -0.35 | -24.82 | 10.42 | 0.16 | 4 |
| 5 μM | -0.07 | 0.06 | -0.36 | -24.71 | 9.27 | 2.90 | 4 |
| 5 μM | -0.07 | 0.05 | -0.37 | -25.21 | 9.22 | 4.09 | 4 |
| 6.5 μM | -0.12 | -0.05 | -0.06 | -22.10 | -11.06 | 0.68 | 5 |
| 6.5 μM | -0.11 | -0.06 | -0.07 | -21.22 | -10.45 | 1.65 | 5 |
| 6.5 μM | -0.11 | -0.06 | -0.08 | -21.38 | -10.18 | 1.80 | 5 |
| 6.5 μM | -0.12 | -0.04 | -0.07 | -21.74 | -10.31 | 0.35 | 5 |
| 6.5 μM | -0.10 | -0.05 | -0.04 | -18.87 | -11.60 | 0.87 | 5 |
| 6.5 μM | -0.11 | -0.04 | -0.08 | -20.64 | -9.29 | 0.76 | 5 |
| 8 μM | 0.05 | 0.14 | -0.07 | -2.65 | 1.01 | -5.63 | 6 |
| 8 μM | 0.07 | 0.16 | -0.09 | -1.80 | 2.83 | -6.05 | 6 |
| 8 μM | 0.07 | 0.15 | -0.05 | -0.64 | 0.58 | -6.44 | 6 |
| 8 μM | 0.06 | 0.17 | -0.07 | -2.40 | 1.86 | -7.64 | 6 |
| 8 μM | 0.06 | 0.13 | -0.09 | -1.74 | 1.44 | -4.34 | 6 |
| 8 μM | 0.06 | 0.15 | -0.07 | -1.69 | 1.27 | -6.17 | 6 |
| 9.5 μM | 0.21 | 0.16 | 0.00 | 18.23 | 1.68 | -3.00 | 7 |
| 9.5 μM | 0.22 | 0.14 | 0.01 | 18.78 | 0.93 | -2.09 | 7 |
| 9.5 μM | 0.21 | 0.13 | 0.02 | 18.33 | -0.32 | -1.67 | 7 |
| 9.5 μM | 0.22 | 0.13 | 0.00 | 19.27 | 0.97 | -1.12 | 7 |
| 9.5 μM | 0.21 | 0.14 | 0.02 | 18.81 | 0.33 | -2.47 | 7 |
| 9.5 μM | 0.22 | 0.13 | -0.02 | 18.83 | 1.61 | -0.35 | 7 |

**Table S18**. LDA jackknifed classification matrix table obtained from an array of nano-enzyme against DA with various concentrations. The jackknifed classification matrix with cross-validation reveals a 100% accuracy.

|  | 0.5 μM | 2 μM | 3.5 μM | 5 μM | 6.5 μM | 8 μM | 9.5 μM | %correct |
| --- | --- | --- | --- | --- | --- | --- | --- | --- |
| 0.5 μM | 6 | 0 | 0 | 0 | 0 | 0 | 0 | 100 |
| 2 μM | 0 | 6 | 0 | 0 | 0 | 0 | 0 | 100 |
| 3.5 μM | 0 | 0 | 6 | 0 | 0 | 0 | 0 | 100 |
| 5 μM | 0 | 0 | 0 | 6 | 0 | 0 | 0 | 100 |
| 6.5 μM | 0 | 0 | 0 | 0 | 6 | 0 | 0 | 100 |
| 8 μM | 0 | 0 | 0 | 0 | 0 | 6 | 0 | 100 |
| 9.5 μM | 0 | 0 | 0 | 0 | 0 | 0 | 6 | 100 |
| Total | 6 | 6 | 6 | 6 | 6 | 6 | 6 | 100 |

**Table S19**. LDA jackknifed classification matrix table obtained from an array of nano-enzyme against EP with various concentrations. The jackknifed classification matrix with cross-validation reveals a 100% accuracy.

|  | 0.5 μM | 2 μM | 3.5 μM | 5 μM | 6.5 μM | 8 μM | 9.5 μM | %correct |
| --- | --- | --- | --- | --- | --- | --- | --- | --- |
| 0.5 μM | 6 | 0 | 0 | 0 | 0 | 0 | 0 | 100 |
| 2 μM | 0 | 6 | 0 | 0 | 0 | 0 | 0 | 100 |
| 3.5 μM | 0 | 0 | 6 | 0 | 0 | 0 | 0 | 100 |
| 5 μM | 0 | 0 | 0 | 6 | 0 | 0 | 0 | 100 |
| 6.5 μM | 0 | 0 | 0 | 0 | 6 | 0 | 0 | 100 |
| 8 μM | 0 | 0 | 0 | 0 | 0 | 6 | 0 | 100 |
| 9.5 μM | 0 | 0 | 0 | 0 | 0 | 0 | 6 | 100 |
| Total | 6 | 6 | 6 | 6 | 6 | 6 | 6 | 100 |

**Table S20**. LDA jackknifed classification matrix table obtained from an array of nano-enzyme against 5-HT with various concentrations. The jackknifed classification matrix with cross-validation reveals a 100% accuracy.

|  | 0.5 μM | 2 μM | 3.5 μM | 5 μM | 6.5 μM | 8 μM | 9.5 μM | %correct |
| --- | --- | --- | --- | --- | --- | --- | --- | --- |
| 0.5 μM | 6 | 0 | 0 | 0 | 0 | 0 | 0 | 100 |
| 2 μM | 0 | 6 | 0 | 0 | 0 | 0 | 0 | 100 |
| 3.5 μM | 0 | 0 | 6 | 0 | 0 | 0 | 0 | 100 |
| 5 μM | 0 | 0 | 0 | 6 | 0 | 0 | 0 | 100 |
| 6.5 μM | 0 | 0 | 0 | 0 | 6 | 0 | 0 | 100 |
| 8 μM | 0 | 0 | 0 | 0 | 0 | 6 | 0 | 100 |
| 9.5 μM | 0 | 0 | 0 | 0 | 0 | 0 | 6 | 100 |
| Total | 6 | 6 | 6 | 6 | 6 | 6 | 6 | 100 |

**Table S21**. LDA jackknifed classification matrix table obtained from an array of nano-enzyme against NE with various concentrations. The jackknifed classification matrix with cross-validation reveals a 100% accuracy.

|  | 0.5 μM | 2 μM | 3.5 μM | 5 μM | 6.5 μM | 8 μM | 9.5 μM | %correct |
| --- | --- | --- | --- | --- | --- | --- | --- | --- |
| 0.5 μM | 6 | 0 | 0 | 0 | 0 | 0 | 0 | 100 |
| 2 μM | 0 | 6 | 0 | 0 | 0 | 0 | 0 | 100 |
| 3.5 μM | 0 | 0 | 6 | 0 | 0 | 0 | 0 | 100 |
| 5 μM | 0 | 0 | 0 | 6 | 0 | 0 | 0 | 100 |
| 6.5 μM | 0 | 0 | 0 | 0 | 6 | 0 | 0 | 100 |
| 8 μM | 0 | 0 | 0 | 0 | 0 | 6 | 0 | 100 |
| 9.5 μM | 0 | 0 | 0 | 0 | 0 | 0 | 6 | 100 |
| Total | 6 | 6 | 6 | 6 | 6 | 6 | 6 | 100 |

**Table S22**. LDA jackknifed classification matrix table obtained from an array of nano-enzyme against HA with various concentrations. The jackknifed classification matrix with cross-validation reveals a 100% accuracy.

|  | 0.5 μM | 2 μM | 3.5 μM | 5 μM | 6.5 μM | 8 μM | 9.5 μM | %correct |
| --- | --- | --- | --- | --- | --- | --- | --- | --- |
| 0.5 μM | 6 | 0 | 0 | 0 | 0 | 0 | 0 | 100 |
| 2 μM | 0 | 6 | 0 | 0 | 0 | 0 | 0 | 100 |
| 3.5 μM | 0 | 0 | 6 | 0 | 0 | 0 | 0 | 100 |
| 5 μM | 0 | 0 | 0 | 6 | 0 | 0 | 0 | 100 |
| 6.5 μM | 0 | 0 | 0 | 0 | 6 | 0 | 0 | 100 |
| 8 μM | 0 | 0 | 0 | 0 | 0 | 6 | 0 | 100 |
| 9.5 μM | 0 | 0 | 0 | 0 | 0 | 0 | 6 | 100 |
| Total | 6 | 6 | 6 | 6 | 6 | 6 | 6 | 100 |

**Table S23**. LDA jackknifed classification matrix table obtained from an array of nano-enzyme against ACh with various concentrations. The jackknifed classification matrix with cross-validation reveals a 100% accuracy.

|  | 0.5 μM | 2 μM | 3.5 μM | 5 μM | 6.5 μM | 8 μM | 9.5 μM | %correct |
| --- | --- | --- | --- | --- | --- | --- | --- | --- |
| 0.5 μM | 6 | 0 | 0 | 0 | 0 | 0 | 0 | 100 |
| 2 μM | 0 | 6 | 0 | 0 | 0 | 0 | 0 | 100 |
| 3.5 μM | 0 | 0 | 6 | 0 | 0 | 0 | 0 | 100 |
| 5 μM | 0 | 0 | 0 | 6 | 0 | 0 | 0 | 100 |
| 6.5 μM | 0 | 0 | 0 | 0 | 6 | 0 | 0 | 100 |
| 8 μM | 0 | 0 | 0 | 0 | 0 | 6 | 0 | 100 |
| 9.5 μM | 0 | 0 | 0 | 0 | 0 | 0 | 6 | 100 |
| Total | 6 | 6 | 6 | 6 | 6 | 6 | 6 | 100 |

**Table S24**. Detection and identification of PFOA with various concentrations using LDA from an array. According to the verification, 35 among 36 unknown samples were correctly identified, representing an accuracy of 97%.

|  | Colorimetric response patterns | | | Results LDA | | | Analyte | |
| --- | --- | --- | --- | --- | --- | --- | --- | --- |
| Sample | ZnTCPP-Zn | ZnTCPP-Ti | ZnTCPP-Al | Factor 1 | Factor 2 | Factor 3 | Identification | Verification |
| 1 | 0.10 | -0.03 | -0.09 | 3.90 | 12.21 | 2.47 | 1 | 1 |
| 2 | 0.10 | -0.03 | -0.10 | 5.56 | 12.59 | 2.13 | 1 | 1 |
| 3 | 0.11 | -0.03 | -0.09 | 4.42 | 12.92 | 2.54 | 1 | 1 |
| 4 | 0.11 | -0.04 | -0.09 | 5.12 | 13.65 | 1.97 | 1 | 1 |
| 5 | 0.01 | -0.06 | -0.13 | 2.03 | 3.71 | -0.75 | 2 | 2 |
| 6 | 0.01 | -0.04 | -0.12 | 0.84 | 3.41 | 0.28 | 2 | 2 |
| 7 | 0.02 | -0.06 | -0.13 | 3.13 | 4.72 | -0.96 | 2 | 2 |
| 8 | 0.02 | -0.06 | -0.13 | 3.42 | 4.35 | -0.95 | 2 | 2 |
| 9 | -0.09 | -0.03 | -0.12 | -6.10 | -7.47 | -0.66 | 6 | 3 |
| 10 | -0.10 | -0.15 | -0.13 | -5.82 | -5.17 | -5.43 | 3 | 3 |
| 11 | -0.08 | -0.16 | -0.11 | -7.23 | -3.23 | -4.66 | 3 | 3 |
| 12 | -0.09 | -0.17 | -0.11 | -8.87 | -3.91 | -4.81 | 3 | 3 |
| 13 | 0.01 | -0.15 | -0.14 | 3.37 | 5.36 | -4.82 | 4 | 4 |
| 14 | 0.00 | -0.15 | -0.15 | 3.14 | 4.28 | -4.87 | 4 | 4 |
| 15 | 0.00 | -0.15 | -0.15 | 3.14 | 4.28 | -4.87 | 4 | 4 |
| 16 | 0.01 | -0.16 | -0.15 | 3.83 | 5.02 | -5.44 | 4 | 4 |
| 17 | -0.03 | -0.04 | 0.06 | -23.68 | 0.83 | 6.09 | 5 | 5 |
| 18 | -0.03 | -0.08 | 0.07 | -25.24 | 1.70 | 5.19 | 5 | 5 |
| 19 | -0.02 | -0.07 | 0.06 | -22.99 | 2.59 | 5.24 | 5 | 5 |
| 20 | -0.03 | -0.08 | 0.10 | -28.87 | 1.63 | 6.37 | 5 | 5 |
| 21 | -0.12 | 0.02 | -0.11 | -9.13 | -11.38 | 1.40 | 6 | 6 |
| 22 | -0.12 | 0.02 | -0.11 | -9.46 | -10.92 | 1.23 | 6 | 6 |
| 23 | -0.12 | 0.01 | -0.11 | -9.34 | -10.49 | 0.93 | 6 | 6 |
| 24 | -0.12 | 0.04 | -0.12 | -8.31 | -12.07 | 1.76 | 6 | 6 |
| 25 | -0.01 | 0.26 | -0.32 | 25.90 | -6.71 | 3.47 | 7 | 7 |
| 26 | -0.01 | 0.27 | -0.29 | 23.15 | -6.85 | 4.39 | 7 | 7 |
| 27 | 0.00 | 0.26 | -0.32 | 26.08 | -6.47 | 3.49 | 7 | 7 |
| 28 | 0.00 | 0.26 | -0.32 | 26.69 | -6.27 | 3.38 | 7 | 7 |

**Table S25**. Detection and identification of EP with various concentrations using LDA from an array. According to the verification, 28 among 28 unknown samples were correctly identified, representing an accuracy of 100%.

|  | Colorimetric response patterns | | | Results LDA | | | Analyte | |
| --- | --- | --- | --- | --- | --- | --- | --- | --- |
| Sample | ZnTCPP-Zn | ZnTCPP-Ti | ZnTCPP-Al | Factor 1 | Factor 2 | Factor 3 | Identification | Verification |
| 1 | 0.08 | -0.06 | -0.15 | 33.99 | -3.94 | 3.91 | 1 | 1 |
| 2 | 0.07 | -0.05 | -0.14 | 34.02 | -4.89 | 3.38 | 1 | 1 |
| 3 | 0.09 | -0.05 | -0.17 | 34.40 | -4.06 | 5.30 | 1 | 1 |
| 4 | 0.08 | -0.07 | -0.16 | 33.38 | -3.09 | 4.23 | 1 | 1 |
| 5 | -0.12 | -0.12 | -0.12 | 17.77 | -15.92 | -1.30 | 2 | 2 |
| 6 | -0.13 | -0.11 | -0.11 | 17.67 | -17.54 | -1.50 | 2 | 2 |
| 7 | -0.12 | -0.12 | -0.11 | 18.20 | -15.07 | -1.96 | 2 | 2 |
| 8 | -0.11 | -0.12 | -0.12 | 18.18 | -14.61 | -1.09 | 2 | 2 |
| 9 | -0.01 | -0.29 | -0.22 | 21.14 | 15.94 | -2.64 | 3 | 3 |
| 10 | 0.03 | -0.28 | -0.22 | 23.86 | 18.10 | -2.14 | 3 | 3 |
| 11 | 0.02 | -0.27 | -0.16 | 25.01 | 16.66 | -4.68 | 3 | 3 |
| 12 | 0.02 | -0.28 | -0.20 | 24.10 | 17.81 | -3.16 | 3 | 3 |
| 13 | -0.27 | -0.39 | -0.35 | -3.88 | 2.54 | -1.42 | 4 | 4 |
| 14 | -0.27 | -0.37 | -0.37 | -4.40 | 0.07 | 0.24 | 4 | 4 |
| 15 | -0.27 | -0.37 | -0.39 | -4.32 | 0.74 | 1.28 | 4 | 4 |
| 16 | -0.27 | -0.36 | -0.32 | -2.82 | -0.54 | -1.75 | 4 | 4 |
| 17 | -0.32 | -0.45 | -0.40 | -10.41 | 5.08 | -1.53 | 5 | 5 |
| 18 | -0.32 | -0.44 | -0.39 | -9.63 | 3.94 | -1.51 | 5 | 5 |
| 19 | -0.32 | -0.44 | -0.39 | -10.13 | 3.13 | -1.88 | 5 | 5 |
| 20 | -0.32 | -0.43 | -0.37 | -9.46 | 2.27 | -2.38 | 5 | 5 |
| 21 | -0.44 | -0.52 | -0.63 | -25.91 | 2.45 | 5.94 | 6 | 6 |
| 22 | -0.45 | -0.51 | -0.63 | -26.18 | 1.10 | 6.60 | 6 | 6 |
| 23 | -0.45 | -0.52 | -0.63 | -26.46 | 1.88 | 6.08 | 6 | 6 |
| 24 | -0.45 | -0.53 | -0.59 | -26.02 | 2.97 | 3.37 | 6 | 6 |
| 25 | -0.57 | -0.56 | -0.51 | -34.88 | -5.39 | -2.48 | 7 | 7 |
| 26 | -0.57 | -0.55 | -0.52 | -34.72 | -6.61 | -1.28 | 7 | 7 |
| 27 | -0.58 | -0.58 | -0.54 | -36.06 | -4.17 | -1.49 | 7 | 7 |
| 28 | -0.57 | -0.56 | -0.52 | -35.05 | -5.12 | -1.63 | 7 | 7 |

**Table S26**. Detection and identification of 5-HT with various concentrations using LDA from an array. According to the verification, 28 among 28 unknown samples were correctly identified, representing an accuracy of 100%.

|  | Colorimetric response patterns | | | Results LDA | | | Analyte | |
| --- | --- | --- | --- | --- | --- | --- | --- | --- |
| Sample | ZnTCPP-Zn | ZnTCPP-Ti | ZnTCPP-Al | Factor 1 | Factor 2 | Factor 3 | Identification | Verification |
| 1 | -0.08 | -0.22 | 0.00 | 22.54 | 7.03 | -1.61 | 1 | 1 |
| 2 | -0.06 | -0.23 | -0.02 | 22.62 | 8.74 | 0.82 | 1 | 1 |
| 3 | -0.07 | -0.23 | 0.00 | 21.76 | 8.31 | -1.01 | 1 | 1 |
| 4 | -0.07 | -0.22 | -0.02 | 22.66 | 6.43 | 0.31 | 1 | 1 |
| 5 | -0.09 | -0.27 | -0.03 | 16.30 | 11.55 | -0.44 | 2 | 2 |
| 6 | -0.09 | -0.27 | -0.03 | 16.26 | 11.47 | -0.14 | 2 | 2 |
| 7 | -0.09 | -0.27 | -0.04 | 16.05 | 11.04 | 1.33 | 2 | 2 |
| 8 | -0.09 | -0.27 | -0.04 | 15.71 | 11.22 | 0.89 | 2 | 2 |
| 9 | -0.19 | -0.14 | -0.13 | 18.69 | -19.34 | 3.99 | 3 | 3 |
| 10 | -0.17 | -0.14 | -0.11 | 21.08 | -17.25 | 4.23 | 3 | 3 |
| 11 | -0.18 | -0.14 | -0.12 | 20.28 | -19.54 | 4.01 | 3 | 3 |
| 12 | -0.19 | -0.14 | -0.14 | 18.94 | -19.69 | 5.03 | 3 | 3 |
| 13 | -0.28 | -0.31 | -0.10 | -3.69 | -5.46 | -5.42 | 4 | 4 |
| 14 | -0.28 | -0.32 | -0.10 | -4.84 | -4.57 | -6.08 | 4 | 4 |
| 15 | -0.28 | -0.32 | -0.12 | -5.34 | -4.86 | -4.04 | 4 | 4 |
| 16 | -0.27 | -0.32 | -0.13 | -4.41 | -3.66 | -3.01 | 4 | 4 |
| 17 | -0.23 | -0.34 | -0.20 | -4.50 | 1.52 | 5.12 | 5 | 5 |
| 18 | -0.25 | -0.34 | -0.18 | -5.65 | 1.35 | 2.56 | 5 | 5 |
| 19 | -0.24 | -0.35 | -0.19 | -5.48 | 2.62 | 4.59 | 5 | 5 |
| 20 | -0.24 | -0.34 | -0.19 | -5.19 | 0.70 | 3.62 | 5 | 5 |
| 21 | -0.33 | -0.44 | -0.26 | -22.99 | 3.77 | 3.51 | 6 | 6 |
| 22 | -0.32 | -0.45 | -0.26 | -22.19 | 5.46 | 3.93 | 6 | 6 |
| 23 | -0.32 | -0.45 | -0.27 | -23.11 | 5.30 | 4.80 | 6 | 6 |
| 24 | -0.32 | -0.46 | -0.26 | -23.48 | 7.31 | 3.76 | 6 | 6 |
| 25 | -0.40 | -0.42 | -0.23 | -26.72 | -6.20 | -3.45 | 7 | 7 |
| 26 | -0.40 | -0.40 | -0.23 | -25.17 | -8.88 | -3.35 | 7 | 7 |
| 27 | -0.41 | -0.42 | -0.21 | -27.41 | -6.90 | -6.25 | 7 | 7 |
| 28 | -0.40 | -0.42 | -0.24 | -26.68 | -6.85 | -2.24 | 7 | 7 |

**Table S27**. Detection and identification of NE with various concentrations using LDA from an array. According to the verification, 28 among 28 unknown samples were correctly identified, representing an accuracy of 100%.

|  | Colorimetric response patterns | | | Results LDA | | | Analyte | |
| --- | --- | --- | --- | --- | --- | --- | --- | --- |
| Sample | ZnTCPP-Zn | ZnTCPP-Ti | ZnTCPP-Al | Factor 1 | Factor 2 | Factor 3 | Identification | Verification |
| 1 | -0.08 | -0.22 | -0.31 | 12.18 | -5.44 | 2.77 | 1 | 1 |
| 2 | -0.08 | -0.23 | -0.31 | 13.36 | -5.38 | 2.82 | 1 | 1 |
| 3 | -0.08 | -0.22 | -0.32 | 12.38 | -5.13 | 3.22 | 1 | 1 |
| 4 | -0.06 | -0.21 | -0.31 | 12.30 | -3.96 | 3.09 | 1 | 1 |
| 5 | 0.07 | -0.23 | -0.25 | 21.60 | 3.77 | 1.49 | 2 | 2 |
| 6 | 0.08 | -0.23 | -0.19 | 23.60 | 4.38 | -0.69 | 2 | 2 |
| 7 | 0.09 | -0.24 | -0.19 | 24.69 | 4.97 | -0.71 | 2 | 2 |
| 8 | 0.10 | -0.22 | -0.22 | 22.46 | 5.25 | 0.69 | 2 | 2 |
| 9 | 0.05 | -0.09 | -0.16 | 2.57 | 3.19 | 0.88 | 3 | 3 |
| 10 | 0.02 | -0.09 | -0.19 | 0.98 | 1.57 | 1.96 | 3 | 3 |
| 11 | 0.01 | -0.09 | -0.15 | 0.18 | 0.98 | 0.12 | 3 | 3 |
| 12 | 0.09 | -0.10 | -0.20 | 5.40 | 5.40 | 2.39 | 3 | 3 |
| 13 | -0.05 | -0.12 | -0.18 | 0.62 | -2.94 | 0.57 | 4 | 4 |
| 14 | -0.05 | -0.13 | -0.22 | 1.57 | -2.92 | 1.86 | 4 | 4 |
| 15 | -0.06 | -0.13 | -0.23 | 1.14 | -3.33 | 2.23 | 4 | 4 |
| 16 | -0.04 | -0.13 | -0.18 | 2.27 | -2.36 | 0.46 | 4 | 4 |
| 17 | 0.14 | 0.05 | -0.06 | -12.20 | 9.48 | 0.83 | 5 | 5 |
| 18 | 0.13 | 0.05 | -0.07 | -12.88 | 8.73 | 0.96 | 5 | 5 |
| 19 | 0.14 | 0.04 | -0.06 | -11.33 | 9.12 | 0.41 | 5 | 5 |
| 20 | 0.15 | 0.06 | -0.04 | -12.02 | 10.21 | 0.16 | 5 | 5 |
| 21 | -0.01 | -0.07 | -0.02 | -1.64 | 0.68 | -4.35 | 6 | 6 |
| 22 | -0.01 | -0.07 | -0.07 | -2.30 | 0.29 | -2.31 | 6 | 6 |
| 23 | -0.03 | -0.08 | -0.07 | -2.47 | -0.57 | -2.49 | 6 | 6 |
| 24 | -0.01 | -0.09 | -0.01 | 1.24 | 0.64 | -4.98 | 6 | 6 |
| 25 | -0.05 | 0.07 | -0.02 | -23.74 | -1.52 | -1.63 | 7 | 7 |
| 26 | -0.08 | 0.08 | -0.03 | -26.90 | -3.11 | -1.13 | 7 | 7 |
| 27 | -0.05 | 0.08 | -0.06 | -25.26 | -1.68 | -0.03 | 7 | 7 |
| 28 | -0.05 | 0.07 | -0.05 | -23.65 | -1.20 | -0.36 | 7 | 7 |

**Table S28**. Detection and identification of NE with various concentrations using LDA from an array. According to the verification, 27 among 28 unknown samples were correctly identified, representing an accuracy of 96%.

|  | Colorimetric response patterns | | | Results LDA | | | Analyte | |
| --- | --- | --- | --- | --- | --- | --- | --- | --- |
| Sample | ZnTCPP-Zn | ZnTCPP-Ti | ZnTCPP-Al | Factor 1 | Factor 2 | Factor 3 | Identification | Verification |
| 1 | -0.08 | 0.23 | -0.02 | -13.07 | -0.20 | 3.45 | 1 | 1 |
| 2 | -0.09 | 0.22 | -0.02 | -11.59 | -0.65 | 2.95 | 1 | 1 |
| 3 | -0.08 | 0.22 | -0.03 | -11.86 | 0.20 | 3.04 | 1 | 1 |
| 4 | -0.07 | 0.23 | -0.01 | -12.29 | 0.14 | 4.19 | 1 | 1 |
| 5 | -0.09 | 0.28 | -0.05 | -19.30 | 0.46 | 3.22 | 2 | 2 |
| 6 | -0.09 | 0.33 | -0.06 | -24.89 | 1.30 | 3.62 | 2 | 2 |
| 7 | -0.08 | 0.33 | -0.06 | -23.91 | 1.94 | 3.98 | 2 | 2 |
| 8 | -0.08 | 0.34 | -0.05 | -24.68 | 1.51 | 4.32 | 2 | 2 |
| 9 | -0.04 | 0.16 | -0.11 | -4.08 | 4.98 | 1.72 | 3 | 3 |
| 10 | -0.04 | 0.16 | -0.11 | -4.82 | 5.21 | 1.97 | 3 | 3 |
| 11 | -0.04 | 0.16 | -0.09 | -4.72 | 4.72 | 2.17 | 3 | 3 |
| 12 | -0.04 | 0.17 | -0.08 | -4.84 | 4.79 | 3.09 | 3 | 3 |
| 13 | -0.15 | 0.11 | -0.05 | -4.19 | -5.31 | -2.76 | 4 | 4 |
| 14 | -0.16 | 0.10 | -0.05 | -4.11 | -6.04 | -3.60 | 4 | 4 |
| 15 | -0.16 | 0.09 | -0.06 | -3.49 | -6.10 | -4.00 | 4 | 4 |
| 16 | -0.15 | 0.09 | -0.05 | -3.13 | -5.40 | -3.24 | 4 | 4 |
| 17 | -0.13 | 0.21 | -0.20 | -17.54 | 1.61 | -4.68 | 5 | 5 |
| 18 | -0.12 | 0.20 | -0.17 | -15.99 | 1.11 | -3.51 | 5 | 5 |
| 19 | -0.12 | 0.19 | -0.17 | -14.68 | 1.03 | -3.72 | 5 | 5 |
| 20 | -0.12 | 0.20 | -0.18 | -15.97 | 1.44 | -3.55 | 5 | 5 |
| 21 | -0.03 | -0.10 | -0.06 | 24.42 | 3.05 | -0.66 | 6 | 6 |
| 22 | -0.02 | -0.10 | -0.07 | 24.46 | 3.70 | -0.75 | 6 | 6 |
| 23 | -0.02 | -0.10 | -0.07 | 25.03 | 3.84 | -0.71 | 6 | 6 |
| 24 | -0.02 | -0.11 | -0.06 | 26.12 | 3.13 | -0.50 | 6 | 6 |
| 25 | -0.04 | -0.09 | 0.09 | 26.44 | -2.78 | 3.06 | 7 | 7 |
| 26 | -0.03 | -0.11 | -0.01 | 26.62 | 0.68 | 0.48 | 7 | 7 |
| 27 | -0.04 | -0.11 | 0.08 | 28.09 | -2.41 | 2.58 | 6 | 7 |
| 28 | -0.03 | -0.09 | 0.10 | 26.90 | -2.57 | 4.02 | 7 | 7 |

**Table S29**. Detection and identification of ACh with various concentrations using LDA from an array. According to the verification, 28 among 28 unknown samples were correctly identified, representing an accuracy of 100%.

|  | Colorimetric response patterns | | | Results LDA | | | Analyte | |
| --- | --- | --- | --- | --- | --- | --- | --- | --- |
| Sample | ZnTCPP-Zn | ZnTCPP-Ti | ZnTCPP-Al | Factor 1 | Factor 2 | Factor 3 | Identification | Verification |
| 1 | 0.22 | 0.05 | -0.01 | 19.49 | -1.32 | 4.38 | 1 | 1 |
| 2 | 0.26 | 0.08 | 0.09 | 26.45 | -4.24 | 1.07 | 1 | 1 |
| 3 | 0.28 | 0.10 | 0.01 | 26.46 | 0.58 | 2.87 | 1 | 1 |
| 4 | 0.27 | 0.11 | 0.08 | 27.95 | -2.21 | 0.03 | 1 | 1 |
| 5 | 0.07 | 0.06 | 0.01 | 1.72 | -5.78 | -1.90 | 2 | 2 |
| 6 | 0.07 | 0.05 | -0.03 | 0.94 | -3.99 | -0.45 | 2 | 2 |
| 7 | 0.07 | 0.06 | -0.02 | 1.80 | -4.24 | -0.90 | 2 | 2 |
| 8 | 0.08 | 0.06 | -0.04 | 1.91 | -3.30 | -0.28 | 2 | 2 |
| 9 | 0.12 | 0.05 | -0.11 | 5.14 | 0.97 | 3.87 | 3 | 3 |
| 10 | 0.12 | 0.07 | -0.09 | 5.15 | 0.98 | 1.72 | 3 | 3 |
| 11 | 0.13 | 0.09 | -0.08 | 6.24 | 1.15 | 0.71 | 3 | 3 |
| 12 | 0.13 | 0.09 | -0.08 | 6.24 | 1.15 | 0.71 | 3 | 3 |
| 13 | -0.06 | 0.09 | -0.35 | -23.89 | 10.24 | 1.07 | 4 | 4 |
| 14 | -0.07 | 0.11 | -0.35 | -24.89 | 10.77 | -0.49 | 4 | 4 |
| 15 | -0.07 | 0.08 | -0.35 | -24.82 | 9.38 | 1.22 | 4 | 4 |
| 16 | -0.07 | 0.05 | -0.30 | -22.60 | 5.81 | 2.34 | 4 | 4 |
| 17 | -0.10 | -0.04 | -0.08 | -20.32 | -9.40 | 1.17 | 5 | 5 |
| 18 | -0.11 | -0.06 | -0.08 | -21.38 | -10.18 | 1.80 | 5 | 5 |
| 19 | -0.12 | -0.06 | -0.08 | -22.50 | -10.60 | 1.77 | 5 | 5 |
| 20 | -0.12 | -0.05 | -0.09 | -22.47 | -9.73 | 1.04 | 5 | 5 |
| 21 | 0.07 | 0.16 | -0.06 | -0.41 | 1.42 | -6.29 | 6 | 6 |
| 22 | 0.07 | 0.14 | -0.06 | -0.85 | 0.59 | -5.17 | 6 | 6 |
| 23 | 0.08 | 0.17 | -0.07 | -0.09 | 2.34 | -6.94 | 6 | 6 |
| 24 | 0.06 | 0.15 | -0.09 | -2.57 | 2.12 | -5.34 | 6 | 6 |
| 25 | 0.20 | 0.12 | 0.02 | 17.36 | -1.02 | -1.85 | 7 | 7 |
| 26 | 0.21 | 0.13 | 0.03 | 18.61 | -0.69 | -2.29 | 7 | 7 |
| 27 | 0.21 | 0.13 | 0.03 | 19.19 | -0.57 | -2.12 | 7 | 7 |
| 28 | 0.22 | 0.14 | 0.01 | 18.97 | 0.49 | -1.91 | 7 | 7 |

**Table S30**. Training matrix of colorimetric response patterns obtained from an array of nano-enzyme against DA/EP with different molar ratios. LDA was carried out and resulting in 3 actors of the canonical scores.

|  | Colorimetric response patterns | | | LDA canonical scores | | | |
| --- | --- | --- | --- | --- | --- | --- | --- |
|  | ZnTCPP-Zn | ZnTCPP-Ti | ZnTCPP-Al | Factor 1 | Factor 2 | Factor 3 | Group |
| 5 μM:0 μM | -0.58 | 0.01 | -0.02 | -52.22 | -7.68 | -0.01 | 5 |
| 5 μM:0 μM | -0.58 | 0.00 | -0.01 | -52.22 | -7.36 | 1.07 | 5 |
| 5 μM:0 μM | -0.58 | 0.01 | 0.01 | -52.12 | -8.02 | 2.10 | 5 |
| 5 μM:0 μM | -0.58 | 0.01 | 0.00 | -52.65 | -7.68 | 1.23 | 5 |
| 5 μM:0 μM | -0.58 | 0.01 | 0.00 | -52.68 | -7.82 | 1.21 | 5 |
| 5 μM:0 μM | -0.58 | 0.00 | -0.01 | -52.14 | -7.34 | 0.87 | 5 |
| 3.5 μM:1.5 μM | -0.42 | -0.14 | -0.16 | -14.86 | 0.82 | -4.57 | 4 |
| 3.5 μM:1.5 μM | -0.40 | -0.13 | -0.14 | -11.44 | 0.42 | -3.24 | 4 |
| 3.5 μM:1.5 μM | -0.40 | -0.13 | -0.16 | -10.95 | 0.70 | -4.45 | 4 |
| 3.5 μM:1.5 μM | -0.39 | -0.10 | -0.16 | -9.87 | -0.55 | -4.01 | 4 |
| 3.5 μM:1.5 μM | -0.40 | -0.13 | -0.09 | -12.58 | 0.08 | -0.18 | 4 |
| 3.5 μM:1.5 μM | -0.40 | -0.13 | -0.14 | -10.99 | 0.40 | -2.77 | 4 |
| 2.5 μM:2.5 μM | -0.18 | -0.09 | -0.17 | 32.89 | 0.60 | -0.98 | 3 |
| 2.5 μM:2.5 μM | -0.18 | -0.08 | -0.17 | 33.02 | 0.17 | -0.79 | 3 |
| 2.5 μM:2.5 μM | -0.18 | -0.08 | -0.18 | 34.27 | 0.18 | -1.74 | 3 |
| 2.5 μM:2.5 μM | -0.18 | -0.08 | -0.17 | 33.45 | 0.09 | -1.18 | 3 |
| 2.5 μM:2.5 μM | -0.18 | -0.09 | -0.19 | 33.59 | 0.47 | -2.20 | 3 |
| 2.5 μM:2.5 μM | -0.18 | -0.15 | -0.17 | 33.42 | 3.12 | -0.75 | 3 |
| 1.5 μM:3.5 μM | -0.02 | 0.01 | -0.18 | 65.98 | -2.51 | 1.31 | 2 |
| 1.5 μM:3.5 μM | -0.02 | -0.01 | -0.18 | 65.04 | -1.69 | 1.07 | 2 |
| 1.5 μM:3.5 μM | -0.01 | -0.01 | -0.17 | 67.67 | -1.95 | 2.14 | 2 |
| 1.5 μM:3.5 μM | -0.01 | -0.01 | -0.18 | 66.73 | -1.79 | 1.43 | 2 |
| 1.5 μM:3.5 μM | -0.02 | -0.01 | -0.18 | 65.34 | -1.68 | 1.10 | 2 |
| 1.5 μM:3.5 μM | -0.02 | -0.02 | -0.14 | 65.68 | -1.63 | 3.68 | 2 |
| 0 μM:5 μM | -0.52 | -0.40 | -0.05 | -35.54 | 11.23 | 1.34 | 1 |
| 0 μM:5 μM | -0.52 | -0.33 | -0.05 | -34.63 | 7.83 | 0.75 | 1 |
| 0 μM:5 μM | -0.52 | -0.28 | -0.04 | -36.92 | 5.71 | 1.46 | 1 |
| 0 μM:5 μM | -0.52 | -0.35 | -0.04 | -35.19 | 9.00 | 1.42 | 1 |
| 0 μM:5 μM | -0.51 | -0.34 | -0.02 | -34.67 | 8.18 | 2.89 | 1 |
| 0 μM:5 μM | -0.52 | -0.35 | -0.04 | -35.40 | 8.68 | 1.80 | 1 |

**Table S31**. Training matrix of colorimetric response patterns obtained from an array of nano-enzyme against DA/NE with different molar ratios. LDA was carried out and resulting in 3 actors of the canonical scores.

|  | Colorimetric response patterns | | | LDA canonical scores | | | |
| --- | --- | --- | --- | --- | --- | --- | --- |
|  | ZnTCPP-Zn | ZnTCPP-Ti | ZnTCPP-Al | Factor 1 | Factor 2 | Factor 3 | Group |
| 5 μM:0 μM | -0.54 | 0.02 | -0.28 | -28.31 | 7.68 | 0.23 | 5 |
| 5 μM:0 μM | -0.53 | 0.02 | -0.27 | -27.47 | 6.64 | 0.39 | 5 |
| 5 μM:0 μM | -0.53 | 0.01 | -0.27 | -26.52 | 7.13 | -0.06 | 5 |
| 5 μM:0 μM | -0.53 | 0.00 | -0.27 | -27.11 | 7.06 | -1.15 | 5 |
| 5 μM:0 μM | -0.53 | 0.00 | -0.26 | -27.14 | 6.28 | -0.98 | 5 |
| 5 μM:0 μM | -0.53 | 0.02 | -0.27 | -26.90 | 6.90 | 0.43 | 5 |
| 3.5 μM:1.5 μM | -0.47 | -0.04 | -0.27 | -17.03 | 6.35 | -3.92 | 4 |
| 3.5 μM:1.5 μM | -0.46 | 0.00 | -0.27 | -14.62 | 6.80 | -1.16 | 4 |
| 3.5 μM:1.5 μM | -0.45 | -0.03 | -0.26 | -14.35 | 6.16 | -2.82 | 4 |
| 3.5 μM:1.5 μM | -0.45 | -0.03 | -0.26 | -14.27 | 5.63 | -2.88 | 4 |
| 3.5 μM:1.5 μM | -0.45 | -0.05 | -0.25 | -14.48 | 4.99 | -4.56 | 4 |
| 3.5 μM:1.5 μM | -0.46 | -0.03 | -0.25 | -15.96 | 4.83 | -3.02 | 4 |
| 2.5 μM:2.5 μM | -0.08 | -0.01 | -0.32 | 47.80 | 10.61 | 0.21 | 3 |
| 2.5 μM:2.5 μM | -0.05 | -0.02 | -0.28 | 52.00 | 7.68 | -0.31 | 3 |
| 2.5 μM:2.5 μM | -0.06 | -0.02 | -0.29 | 50.64 | 8.74 | -0.22 | 3 |
| 2.5 μM:2.5 μM | -0.05 | 0.03 | -0.29 | 50.80 | 8.42 | 2.93 | 3 |
| 2.5 μM:2.5 μM | -0.06 | -0.04 | -0.31 | 51.64 | 9.70 | -1.67 | 3 |
| 2.5 μM:2.5 μM | -0.06 | -0.01 | -0.28 | 50.40 | 7.44 | 0.15 | 3 |
| 1.5 μM:3.5 μM | -0.45 | 0.07 | -0.17 | -17.11 | -1.08 | 3.40 | 2 |
| 1.5 μM:3.5 μM | -0.44 | 0.09 | -0.19 | -14.54 | 0.87 | 5.03 | 2 |
| 1.5 μM:3.5 μM | -0.44 | 0.07 | -0.18 | -14.65 | 0.00 | 3.86 | 2 |
| 1.5 μM:3.5 μM | -0.44 | 0.07 | -0.21 | -14.61 | 2.38 | 3.88 | 2 |
| 1.5 μM:3.5 μM | -0.44 | 0.05 | -0.21 | -14.48 | 2.31 | 2.81 | 2 |
| 1.5 μM:3.5 μM | -0.44 | 0.06 | -0.19 | -15.23 | 0.23 | 3.34 | 2 |
| 0 μM:5 μM | -0.26 | 0.04 | 0.09 | 6.86 | -23.03 | -1.10 | 1 |
| 0 μM:5 μM | -0.26 | 0.04 | 0.07 | 7.24 | -21.68 | -0.66 | 1 |
| 0 μM:5 μM | -0.26 | 0.04 | 0.09 | 6.94 | -22.76 | -1.06 | 1 |
| 0 μM:5 μM | -0.26 | 0.04 | 0.07 | 7.40 | -21.15 | -0.59 | 1 |
| 0 μM:5 μM | -0.26 | 0.06 | 0.10 | 6.50 | -23.46 | 0.44 | 1 |
| 0 μM:5 μM | -0.27 | 0.04 | 0.07 | 6.55 | -21.70 | -0.94 | 1 |

**Table S32**. LDA jackknifed classification matrix table obtained from an array of nanozymes against DA/EP with different molar ratios. The jackknifed classification matrix with cross-validation reveals a 100% accuracy.

|  | 0 μM:5 μM | 1.5 μM:3.5 μM | 2.5 μM:2.5 μM | 3.5 μM:1.5 μM | 5 μM:0 μM | %correct |
| --- | --- | --- | --- | --- | --- | --- |
| 0 μM:5 μM | 6 | 0 | 0 | 0 | 0 | 100 |
| 1.5 μM:3.5 μM | 0 | 6 | 0 | 0 | 0 | 100 |
| 2.5 μM:2.5 μM | 0 | 0 | 6 | 0 | 0 | 100 |
| 3.5 μM:1.5 μM | 0 | 0 | 0 | 6 | 0 | 100 |
| 5 μM:0 μM | 0 | 0 | 0 | 0 | 6 | 100 |
| Total | 6 | 6 | 6 | 6 | 6 | 100 |

**Table S33**. LDA jackknifed classification matrix table obtained from an array of nanozymes against DA/NE with different molar ratios. The jackknifed classification matrix with cross-validation reveals a 100% accuracy.

|  | 0 μM:5 μM | 1.5 μM:3.5 μM | 2.5 μM:2.5 μM | 3.5 μM:1.5 μM | 5 μM:0 μM | %correct |
| --- | --- | --- | --- | --- | --- | --- |
| 0 μM:5 μM | 6 | 0 | 0 | 0 | 0 | 100 |
| 1.5 μM:3.5 μM | 0 | 6 | 0 | 0 | 0 | 100 |
| 2.5 μM:2.5 μM | 0 | 0 | 6 | 0 | 0 | 100 |
| 3.5 μM:1.5 μM | 0 | 0 | 0 | 6 | 0 | 100 |
| 5 μM:0 μM | 0 | 0 | 0 | 0 | 6 | 100 |
| Total | 6 | 6 | 6 | 6 | 6 | 100 |

**Table S34**. Detection and identification of unknown samples with different molar ratios of DA/EP using LDA from the array of nanozymes. According to the verification, 20 among 20 unknown samples were correctly identified, representing an accuracy of 100%.

| Sample | Colorimetric response patterns | | | Results LDA | | | Analyte | |
| --- | --- | --- | --- | --- | --- | --- | --- | --- |
| # | ZnTCPP-Zn | ZnTCPP-Ti | ZnTCPP-Al | Factor 1 | Factor 2 | Factor 3 | Identification | Verification |
| 1 | -0.59 | -0.02 | 0.01 | -53.35 | -6.81 | 2.07 | 5 | 5 |
| 2 | -0.59 | -0.02 | -0.04 | -52.21 | -6.47 | -0.98 | 5 | 5 |
| 3 | -0.58 | -0.02 | -0.01 | -51.59 | -6.47 | 0.77 | 5 | 5 |
| 4 | -0.59 | -0.03 | -0.02 | -52.85 | -6.05 | 0.26 | 5 | 5 |
| 5 | -0.41 | -0.15 | -0.19 | -12.07 | 1.50 | -5.97 | 4 | 4 |
| 6 | -0.41 | -0.15 | -0.18 | -11.04 | 1.75 | -5.23 | 4 | 4 |
| 7 | -0.41 | -0.12 | -0.18 | -11.48 | 0.39 | -5.79 | 4 | 4 |
| 8 | -0.41 | -0.15 | -0.17 | -11.19 | 1.71 | -4.82 | 4 | 4 |
| 9 | -0.24 | -0.10 | -0.16 | 21.98 | 0.32 | -1.55 | 3 | 3 |
| 10 | -0.18 | -0.09 | -0.13 | 33.64 | 0.09 | 1.81 | 3 | 3 |
| 11 | -0.18 | -0.10 | -0.16 | 34.02 | 1.02 | -0.43 | 3 | 3 |
| 12 | -0.18 | -0.08 | -0.18 | 34.77 | 0.04 | -1.49 | 3 | 3 |
| 13 | -0.01 | -0.02 | -0.18 | 66.79 | -1.51 | 1.46 | 2 | 2 |
| 14 | -0.01 | -0.02 | -0.19 | 67.15 | -1.14 | 0.67 | 2 | 2 |
| 15 | -0.01 | -0.03 | -0.18 | 68.23 | -1.00 | 1.19 | 2 | 2 |
| 16 | -0.05 | 0.01 | -0.18 | 59.06 | -2.89 | 0.67 | 2 | 2 |
| 17 | -0.52 | -0.34 | -0.04 | -35.91 | 8.42 | 1.32 | 1 | 1 |
| 18 | -0.53 | -0.35 | -0.04 | -36.52 | 8.66 | 1.49 | 1 | 1 |
| 19 | -0.53 | -0.35 | -0.04 | -37.66 | 8.90 | 1.41 | 1 | 1 |
| 20 | -0.52 | -0.34 | -0.05 | -34.96 | 8.34 | 1.18 | 1 | 1 |

**Table S35**. Detection and identification of unknown samples with different molar ratios of DA/EP using LDA from the array of nanozymes. According to the verification, 20 among 20 unknown samples were correctly identified, representing an accuracy of 100%.

| Sample | Colorimetric response patterns | | | Results LDA | | | Analyte | |
| --- | --- | --- | --- | --- | --- | --- | --- | --- |
| # | ZnTCPP-Zn | ZnTCPP-Ti | ZnTCPP-Al | Factor 1 | Factor 2 | Factor 3 | Identification | Verification |
| 1 | -0.54 | 0.02 | -0.25 | -28.97 | 4.75 | -0.14 | 5 | 5 |
| 2 | -0.52 | 0.03 | -0.28 | -25.68 | 8.01 | 1.12 | 5 | 5 |
| 3 | -0.52 | 0.00 | -0.27 | -26.10 | 6.55 | -0.92 | 5 | 5 |
| 4 | -0.53 | 0.02 | -0.26 | -27.10 | 5.59 | 0.53 | 5 | 5 |
| 5 | -0.46 | -0.03 | -0.26 | -15.73 | 6.12 | -3.39 | 4 | 4 |
| 6 | -0.46 | -0.05 | -0.28 | -14.89 | 7.35 | -4.81 | 4 | 4 |
| 7 | -0.46 | -0.05 | -0.24 | -15.66 | 4.17 | -4.95 | 4 | 4 |
| 8 | -0.46 | -0.05 | -0.25 | -15.41 | 4.97 | -4.85 | 4 | 4 |
| 9 | -0.05 | -0.02 | -0.26 | 51.75 | 6.08 | -0.51 | 3 | 3 |
| 10 | -0.05 | -0.02 | -0.30 | 51.67 | 9.50 | -0.63 | 3 | 3 |
| 11 | -0.06 | -0.05 | -0.41 | 54.14 | 18.15 | -1.65 | 3 | 3 |
| 12 | -0.06 | -0.02 | -0.50 | 55.61 | 25.79 | 1.97 | 3 | 3 |
| 13 | -0.44 | 0.06 | -0.20 | -14.03 | 1.53 | 2.99 | 2 | 2 |
| 14 | -0.44 | 0.07 | -0.19 | -14.86 | 0.79 | 3.68 | 2 | 2 |
| 15 | -0.44 | 0.06 | -0.18 | -15.24 | -0.07 | 2.77 | 2 | 2 |
| 16 | -0.44 | 0.07 | -0.18 | -15.34 | -0.01 | 3.57 | 2 | 2 |
| 17 | -0.27 | 0.05 | 0.08 | 6.49 | -22.16 | 0.06 | 1 | 1 |
| 18 | -0.27 | 0.03 | 0.07 | 6.49 | -21.49 | -1.71 | 1 | 1 |
| 19 | -0.27 | 0.02 | 0.09 | 6.09 | -23.14 | -2.72 | 1 | 1 |
| 20 | -0.26 | 0.02 | 0.09 | 7.35 | -22.85 | -2.39 | 1 | 1 |

**Table S36**. Training matrix of the colorimetric response patterns obtained from an array of nanozymes against 6 neurotransmitters in cerebro-spinal fluid. LDA was carried out and resulting in 3 factors of the canonical scores.

|  | Colorimetric response patterns | | | LDA canonical scores | | | |
| --- | --- | --- | --- | --- | --- | --- | --- |
|  | ZnTCPP-Zn | ZnTCPP-Ti | ZnTCPP-Al | Factor 1 | Factor 2 | Factor 3 | Group |
| NE | -0.02 | 0.06 | 0.00 | 11.00 | -5.48 | -3.15 | 6 |
| NE | 0.00 | 0.02 | -0.01 | 13.24 | -3.31 | -1.63 | 6 |
| NE | -0.01 | 0.02 | -0.01 | 11.71 | -3.97 | -1.81 | 6 |
| NE | -0.01 | 0.01 | -0.03 | 12.32 | -2.24 | -1.56 | 6 |
| NE | -0.01 | 0.01 | -0.02 | 12.27 | -3.16 | -1.59 | 6 |
| NE | 0.00 | 0.02 | -0.03 | 12.58 | -1.80 | -1.66 | 6 |
| EP | -0.33 | -0.14 | -0.38 | -50.59 | 0.20 | -3.34 | 4 |
| EP | -0.33 | -0.22 | -0.35 | -49.12 | -2.06 | -1.09 | 4 |
| EP | -0.33 | -0.22 | -0.38 | -49.32 | 0.48 | -0.77 | 4 |
| EP | -0.33 | -0.26 | -0.41 | -50.09 | 2.25 | 0.42 | 4 |
| EP | -0.34 | -0.26 | -0.41 | -51.89 | 1.18 | 0.20 | 4 |
| EP | -0.33 | -0.26 | -0.40 | -50.82 | 0.81 | 0.03 | 4 |
| 5-HT | -0.12 | -0.18 | -0.15 | -9.11 | -2.02 | 2.02 | 1 |
| 5-HT | -0.10 | -0.18 | -0.14 | -5.89 | -2.10 | 2.22 | 1 |
| 5-HT | -0.10 | -0.18 | -0.14 | -5.72 | -1.43 | 2.26 | 1 |
| 5-HT | -0.10 | -0.18 | -0.15 | -6.41 | -0.90 | 2.47 | 1 |
| 5-HT | -0.11 | -0.22 | -0.14 | -6.52 | -2.69 | 3.49 | 1 |
| 5-HT | -0.11 | -0.19 | -0.16 | -8.25 | -0.85 | 2.55 | 1 |
| DA | 0.00 | -0.01 | -0.13 | 9.96 | 5.22 | -0.78 | 3 |
| DA | -0.01 | -0.06 | -0.12 | 9.77 | 3.63 | 0.51 | 3 |
| DA | 0.00 | -0.06 | -0.10 | 11.26 | 3.34 | 0.79 | 3 |
| DA | 0.00 | 0.04 | -0.13 | 9.68 | 5.81 | -2.55 | 3 |
| DA | 0.00 | -0.06 | -0.13 | 9.98 | 5.21 | 0.58 | 3 |
| DA | -0.01 | -0.05 | -0.13 | 8.66 | 4.08 | 0.27 | 3 |
| HA | 0.04 | -0.03 | -0.04 | 19.12 | 0.95 | 0.60 | 5 |
| HA | 0.05 | 0.08 | -0.03 | 21.20 | 2.36 | -2.66 | 5 |
| HA | 0.05 | -0.01 | -0.03 | 20.69 | 1.51 | -0.03 | 5 |
| HA | 0.04 | -0.01 | -0.02 | 20.65 | 0.56 | 0.07 | 5 |
| HA | 0.04 | -0.03 | -0.04 | 19.28 | 1.61 | 0.63 | 5 |
| HA | 0.05 | -0.03 | -0.04 | 21.30 | 2.21 | 0.73 | 5 |
| Ach | 0.01 | -0.03 | -0.03 | 15.51 | -1.19 | 0.16 | 2 |
| Ach | 0.01 | -0.05 | -0.02 | 15.50 | -2.19 | 0.53 | 2 |
| Ach | 0.02 | -0.05 | -0.03 | 15.95 | -1.13 | 0.75 | 2 |
| Ach | 0.01 | -0.05 | -0.03 | 14.54 | -2.07 | 0.71 | 2 |
| Ach | 0.00 | -0.05 | -0.04 | 13.82 | -1.48 | 0.65 | 2 |
| Ach | 0.00 | -0.03 | -0.04 | 13.76 | -1.35 | -0.03 | 2 |

**Table 37**. Training matrix of the colorimetric response patterns obtained from an array of nanozymes against 6 neurotransmitters in serum. LDA was carried out and resulting in 3 factors of the canonical scores.

|  | Colorimetric response patterns | | | LDA canonical scores | | | |
| --- | --- | --- | --- | --- | --- | --- | --- |
|  | ZnTCPP-Zn | ZnTCPP-Ti | ZnTCPP-Al | Factor 1 | Factor 2 | Factor 3 | Group |
| NE | 0.00 | -0.10 | -0.09 | 6.83 | 9.10 | -1.43 | 6 |
| NE | 0.02 | -0.10 | -0.06 | 10.84 | 7.77 | -1.00 | 6 |
| NE | 0.01 | -0.09 | -0.08 | 8.47 | 9.08 | -1.14 | 6 |
| NE | 0.00 | -0.08 | -0.08 | 8.57 | 8.76 | -2.72 | 6 |
| NE | 0.02 | -0.09 | -0.06 | 10.84 | 8.39 | -1.63 | 6 |
| NE | 0.03 | -0.08 | -0.07 | 10.38 | 10.01 | -1.01 | 6 |
| EP | -0.31 | -0.37 | -0.31 | -36.02 | -5.85 | -2.67 | 4 |
| EP | -0.31 | -0.38 | -0.30 | -34.83 | -7.07 | -2.55 | 4 |
| EP | -0.31 | -0.38 | -0.31 | -35.59 | -6.43 | -2.22 | 4 |
| EP | -0.31 | -0.37 | -0.32 | -35.94 | -5.19 | -2.37 | 4 |
| EP | -0.31 | -0.38 | -0.33 | -37.45 | -5.04 | -1.62 | 4 |
| EP | -0.31 | -0.37 | -0.32 | -36.16 | -5.50 | -2.37 | 4 |
| 5-HT | -0.14 | -0.34 | -0.23 | -19.05 | -0.11 | 6.80 | 1 |
| 5-HT | -0.15 | -0.30 | -0.22 | -18.33 | 0.64 | 2.75 | 1 |
| 5-HT | -0.14 | -0.29 | -0.23 | -17.84 | 1.86 | 3.39 | 1 |
| 5-HT | -0.14 | -0.29 | -0.22 | -17.72 | 1.45 | 3.44 | 1 |
| 5-HT | -0.14 | -0.30 | -0.22 | -17.84 | 1.23 | 4.03 | 1 |
| 5-HT | -0.13 | -0.30 | -0.24 | -18.76 | 2.16 | 5.01 | 1 |
| DA | -0.09 | -0.19 | -0.14 | -4.11 | 3.02 | -1.62 | 3 |
| DA | -0.08 | -0.18 | -0.15 | -4.91 | 4.73 | -1.04 | 3 |
| DA | -0.09 | -0.17 | -0.14 | -4.10 | 4.22 | -2.07 | 3 |
| DA | -0.08 | -0.18 | -0.14 | -4.08 | 4.75 | -1.07 | 3 |
| DA | -0.05 | -0.18 | -0.14 | -2.77 | 6.40 | 1.25 | 3 |
| DA | -0.09 | -0.19 | -0.13 | -3.64 | 3.08 | -1.34 | 3 |
| HA | -0.03 | -0.20 | 0.00 | 11.19 | -2.82 | 0.56 | 5 |
| HA | -0.03 | -0.22 | 0.01 | 12.08 | -4.55 | 1.74 | 5 |
| HA | -0.03 | -0.20 | 0.01 | 12.38 | -3.42 | 0.05 | 5 |
| HA | -0.01 | -0.20 | 0.00 | 12.52 | -2.21 | 1.81 | 5 |
| HA | -0.04 | -0.19 | -0.01 | 10.72 | -2.25 | -0.35 | 5 |
| HA | -0.01 | -0.16 | -0.01 | 12.40 | 0.09 | -0.15 | 5 |
| Ach | 0.08 | -0.12 | 0.20 | 37.04 | -6.30 | -0.74 | 2 |
| Ach | 0.09 | -0.15 | 0.19 | 36.58 | -6.56 | 1.79 | 2 |
| Ach | 0.07 | -0.13 | 0.18 | 35.07 | -6.22 | -0.27 | 2 |
| Ach | 0.08 | -0.11 | 0.20 | 38.13 | -5.91 | -1.12 | 2 |
| Ach | 0.09 | -0.12 | 0.20 | 38.06 | -5.52 | 0.11 | 2 |
| Ach | 0.08 | -0.12 | 0.19 | 37.03 | -5.79 | -0.21 | 2 |

**Table S38**. LDA jackknifed classification matrix table obtained from an array of nanozymes against 6 neurotransmitters in cerebro-spinal fluid. The jackknifed classification matrix with cross-validation reveals a 100% accuracy.

|  | 5-HT | Ach | DA | EP | HA | NE | %correct |
| --- | --- | --- | --- | --- | --- | --- | --- |
| 5-HT | 6 | 0 | 0 | 0 | 0 | 0 | 100 |
| Ach | 0 | 6 | 0 | 0 | 0 | 0 | 100 |
| DA | 0 | 0 | 6 | 0 | 0 | 0 | 100 |
| EP | 0 | 0 | 0 | 6 | 0 | 0 | 100 |
| HA | 0 | 0 | 0 | 0 | 6 | 0 | 100 |
| NE | 0 | 0 | 0 | 0 | 0 | 6 | 100 |
| Total | 6 | 6 | 6 | 6 | 6 | 6 | 100 |

**Table S39**. LDA jackknifed classification matrix table obtained from an array of nanozymes against 6 neurotransmitters in serum. The jackknifed classification matrix with cross-validation reveals a 100% accuracy.

|  | 5-HT | Ach | DA | EP | HA | NE | %correct |
| --- | --- | --- | --- | --- | --- | --- | --- |
| 5-HT | 6 | 0 | 0 | 0 | 0 | 0 | 100 |
| Ach | 0 | 6 | 0 | 0 | 0 | 0 | 100 |
| DA | 0 | 0 | 6 | 0 | 0 | 0 | 100 |
| EP | 0 | 0 | 0 | 6 | 0 | 0 | 100 |
| HA | 0 | 0 | 0 | 0 | 6 | 0 | 100 |
| NE | 0 | 0 | 0 | 0 | 0 | 6 | 100 |
| Total | 6 | 6 | 6 | 6 | 6 | 6 | 100 |

**Table S40**. Detection and identification of unknown samples at 10 μM using LDA from the array of nanozymes. According to the verification, 24 among 24 unknown samples were correctly identified, representing an accuracy of 100%.

| Sample | Colorimetric response patterns | | | Results LDA | | | Analyte | |
| --- | --- | --- | --- | --- | --- | --- | --- | --- |
| # | ZnTCPP-Zn | ZnTCPP-Ti | ZnTCPP-Al | Factor 1 | Factor 2 | Factor 3 | Identification | Verification |
| 1 | -0.01 | 0.01 | -0.04 | 11.33 | -2.07 | -1.66 | 6 | 6 |
| 2 | -0.02 | 0.01 | -0.05 | 9.75 | -1.58 | -1.67 | 6 | 6 |
| 3 | -0.01 | 0.02 | -0.03 | 11.65 | -2.80 | -1.78 | 6 | 6 |
| 4 | -0.01 | 0.00 | -0.03 | 12.36 | -2.32 | -1.15 | 6 | 6 |
| 5 | -0.33 | -0.26 | -0.41 | -51.35 | 2.01 | 0.29 | 4 | 4 |
| 6 | -0.34 | -0.24 | -0.42 | -51.90 | 2.23 | -0.44 | 4 | 4 |
| 7 | -0.34 | -0.27 | -0.41 | -52.74 | 1.05 | 0.38 | 4 | 4 |
| 8 | -0.33 | -0.26 | -0.41 | -50.96 | 2.18 | 0.33 | 4 | 4 |
| 9 | -0.11 | -0.19 | -0.13 | -6.76 | -3.20 | 2.64 | 1 | 1 |
| 10 | -0.11 | -0.19 | -0.15 | -8.32 | -1.74 | 2.38 | 1 | 1 |
| 11 | -0.11 | -0.18 | -0.15 | -8.23 | -1.94 | 2.12 | 1 | 1 |
| 12 | -0.10 | -0.19 | -0.15 | -6.40 | -0.92 | 2.61 | 1 | 1 |
| 13 | -0.01 | -0.05 | -0.12 | 8.47 | 3.46 | -0.04 | 3 | 3 |
| 14 | -0.01 | -0.05 | -0.13 | 8.05 | 4.42 | 0.22 | 3 | 3 |
| 15 | -0.01 | -0.06 | -0.14 | 8.73 | 4.94 | 0.57 | 3 | 3 |
| 16 | -0.02 | -0.07 | -0.13 | 7.05 | 3.60 | 0.49 | 3 | 3 |
| 17 | 0.06 | -0.03 | -0.01 | 23.56 | 0.16 | 1.04 | 5 | 5 |
| 18 | 0.04 | -0.02 | -0.03 | 20.56 | 0.76 | 0.34 | 5 | 5 |
| 19 | 0.05 | -0.02 | -0.03 | 21.34 | 1.07 | 0.56 | 5 | 5 |
| 20 | 0.04 | -0.02 | -0.03 | 19.69 | 0.69 | 0.24 | 5 | 5 |
| 21 | 0.00 | -0.05 | -0.04 | 13.32 | -1.36 | 0.46 | 2 | 2 |
| 22 | 0.01 | -0.05 | -0.04 | 14.56 | -1.10 | 0.47 | 2 | 2 |
| 23 | 0.01 | -0.06 | -0.05 | 13.90 | -0.61 | 0.95 | 2 | 2 |
| 24 | 0.01 | -0.06 | -0.05 | 14.16 | -0.17 | 0.86 | 2 | 2 |

**Table S41**. Detection and identification of unknown samples in serum using LDA from the array of nanozymes. According to the verification, 24 among 24 unknown samples were correctly identified, representing an accuracy of 100%.

| Sample | Colorimetric response patterns | | | Results LDA | | | Analyte | |
| --- | --- | --- | --- | --- | --- | --- | --- | --- |
| # | ZnTCPP-Zn | ZnTCPP-Ti | ZnTCPP-Al | Factor 1 | Factor 2 | Factor 3 | Identification | Verification |
| 1 | 0.03 | -0.09 | -0.07 | 10.20 | 9.72 | -0.54 | 6 | 6 |
| 2 | 0.03 | -0.09 | -0.07 | 10.42 | 9.40 | 0.09 | 6 | 6 |
| 3 | 0.03 | -0.09 | -0.05 | 12.03 | 8.26 | -0.79 | 6 | 6 |
| 4 | 0.03 | -0.07 | -0.06 | 11.65 | 10.07 | -1.23 | 6 | 6 |
| 5 | -0.29 | -0.37 | -0.32 | -35.51 | -4.67 | -1.21 | 4 | 4 |
| 6 | -0.30 | -0.38 | -0.32 | -36.74 | -4.72 | -0.52 | 4 | 4 |
| 7 | -0.30 | -0.39 | -0.32 | -36.23 | -5.74 | -0.49 | 4 | 4 |
| 8 | -0.31 | -0.37 | -0.31 | -35.67 | -5.37 | -2.44 | 4 | 4 |
| 9 | -0.14 | -0.31 | -0.23 | -19.00 | 1.27 | 4.83 | 1 | 1 |
| 10 | -0.15 | -0.31 | -0.22 | -18.53 | 0.24 | 4.39 | 1 | 1 |
| 11 | -0.13 | -0.19 | -0.24 | -16.48 | 7.70 | -2.79 | 1 | 1 |
| 12 | -0.15 | -0.30 | -0.23 | -18.92 | 1.47 | 3.77 | 1 | 1 |
| 13 | -0.09 | -0.17 | -0.14 | -4.32 | 4.54 | -2.70 | 3 | 3 |
| 14 | -0.08 | -0.18 | -0.15 | -5.19 | 4.92 | -0.97 | 3 | 3 |
| 15 | -0.07 | -0.19 | -0.16 | -5.23 | 5.37 | -0.09 | 3 | 3 |
| 16 | -0.09 | -0.18 | -0.16 | -5.76 | 4.76 | -1.83 | 3 | 3 |
| 17 | -0.01 | -0.19 | 0.01 | 13.65 | -1.79 | 1.90 | 5 | 5 |
| 18 | -0.02 | -0.19 | 0.00 | 12.58 | -2.08 | 1.11 | 5 | 5 |
| 19 | -0.02 | -0.19 | 0.01 | 12.77 | -2.30 | 0.10 | 5 | 5 |
| 20 | -0.03 | -0.19 | 0.00 | 11.58 | -2.80 | -0.10 | 5 | 5 |
| 21 | 0.09 | -0.12 | 0.19 | 37.39 | -4.73 | 0.20 | 2 | 2 |
| 22 | 0.08 | -0.13 | 0.18 | 35.84 | -5.19 | 0.31 | 2 | 2 |
| 23 | 0.08 | -0.13 | 0.17 | 34.89 | -4.84 | 1.10 | 2 | 2 |
| 24 | 0.08 | -0.12 | 0.18 | 35.25 | -4.83 | -0.02 | 2 | 2 |

**Table S42.** Comparison between the nanozyme sensor array and conventional detection methods.

| Parameter | Nanozyme Sensor Array | LC-MS/MS | ELISA | Fluorescence Assays |
| --- | --- | --- | --- | --- |
| Detection time | < 10 min | 30–90 min | 2–4 hours | 30–60 min |
| Sensitivity | High sensitivity; No pretreatment; direct detection (0.1 μM) | High sensitivity; Reduced sensitivity in complex biological matrices | High sensitivity; | High sensitivity; No pretreatment; direct detection (0.1 μM) |
| Specificity | 100% classification accuracy; Simultaneous detection of multiple neurotransmitters | Requires extensive sample preparation; Multiple runs required | Analyte-specific antibodies required; Typically one analyte per assay | Low specificity; Labeling often required |
| Cost and operational complexity | Low-cost nanozyme materials; Simple fabrication; Simple procedures; No highly trained personnel required | High cost; Complex operation; Consumables and maintenance; Requires skilled technical operators | Costly antibodies and reagents; Requires trained personnel | Low-cost; Requires skilled personnel for reliable performance |
| Ref. | This work | [4] | [5] | [6] |

**Table S43.** Training matrix of the colorimetric response patterns obtained from an array of nanozymes against normal and AD sample. LDA was carried out and resulting in factors of the canonical scores.

|  | Colorimetric response patterns | | | Results LDA |
| --- | --- | --- | --- | --- |
|  | ZnTCPP-Zn | ZnTCPP-Ti | ZnTCPP-Al | Factor 1 |
| Normal | 0.49 | -0.23 | 0.13 | 1.78 |
| Normal | 0.49 | -0.23 | 0.13 | 1.78 |
| Normal | 0.50 | -0.23 | 0.12 | 1.76 |
| Normal | 0.51 | -0.24 | 0.12 | 1.81 |
| Normal | 0.62 | -0.18 | 0.13 | 1.71 |
| Normal | 0.62 | -0.17 | 0.12 | 1.68 |
| Normal | 0.61 | -0.16 | 0.13 | 1.63 |
| Normal | 0.36 | -0.19 | 0.17 | 1.50 |
| Normal | 0.67 | -0.14 | 0.14 | 1.63 |
| Normal | 0.72 | -0.11 | 0.15 | 1.61 |
| Normal | 0.74 | -0.10 | 0.14 | 1.59 |
| AD | 0.34 | -0.34 | 0.18 | 2.12 |
| AD | 0.40 | -0.32 | 0.20 | 2.14 |
| AD | 0.41 | -0.32 | 0.22 | 2.16 |
| AD | 0.39 | -0.32 | 0.48 | 2.58 |
| AD | 0.45 | -0.29 | 0.17 | 2.03 |
| AD | 0.40 | -0.31 | 0.16 | 2.05 |
| AD | 0.44 | -0.31 | 0.14 | 2.05 |
| AD | 0.44 | -0.31 | 0.16 | 2.07 |
| AD | 0.53 | -0.39 | 0.10 | 2.41 |
| AD | 0.60 | -0.37 | 0.12 | 2.46 |
| AD | 0.60 | -0.37 | 0.12 | 2.46 |

**Table S44.** Predictive performance of different machine learning models for AD diagnosis.

| Model | Precision | Recall | F1 score | AUC |
| --- | --- | --- | --- | --- |
| BNB | 0.9846±0.0493 | 0.9795±0.0679 | 0.9775±0.0756 | 0.9990±0.0070 |
| GPC | 0.9753±0.0408 | 0.9695±0.0506 | 0.9692±0.0507 | 0.9885±0.0299 |
| KNN | 0.9182±0.0867 | 0.8968±0.1152 | 0.886±0.1331 | 0.9648±0.0559 |
| LDA | 1.0000±0.0000 | 1.0000±0.0000 | 1.0000±0.0000 | 1.0000±0.0000 |
| RF | 0.9424±0.0681 | 0.9268±0.0901 | 0.9232±0.0959 | 0.9895±0.0258 |
| DT | 1.0000±0.0000 | 1.0000±0.0000 | 1.0000±0.0000 | 1.0000±0.0000 |
| SVM | 0.9534±0.0842 | 0.9463±0.0792 | 0.943±0.0926 | 0.9900±0.0255 |
| LR | 0.9892±0.0359 | 0.986±0.0503 | 0.9848±0.0586 | 1.0000±0.0000 |
| MLP | 0.9868±0.0315 | 0.9822±0.0425 | 0.9827±0.0412 | 0.9995±0.0050 |

**Table S45.** Training matrix of the colorimetric response patterns obtained from an array of nanozymes against clinical serum samples from healthy controls, AD, and PD patients.

|  | Colorimetric response patterns | | |
| --- | --- | --- | --- |
|  | ZnTCPP-Zn | ZnTCPP-Al | ZnTCPP-Ti |
| Normal | 0.63 | 0.62 | 0.55 |
| Normal | 0.80 | 0.62 | 0.56 |
| Normal | 0.51 | 0.49 | 0.58 |
| Normal | 0.44 | 0.50 | 0.66 |
| Normal | 0.85 | 0.53 | 0.55 |
| Normal | 0.81 | 0.56 | 0.58 |
| Normal | 0.50 | 0.49 | 0.51 |
| Normal | 0.44 | 0.57 | 0.56 |
| Normal | 0.39 | 0.65 | 0.67 |
| Normal | 0.46 | 0.64 | 0.74 |
| Normal | 0.35 | 0.64 | 0.81 |
| Normal | 0.36 | 0.61 | 0.69 |
| Normal | 0.44 | 0.67 | 0.66 |
| Normal | 0.66 | 0.61 | 0.79 |
| Normal | 0.42 | 0.63 | 0.76 |
| Normal | 0.44 | 0.55 | 0.71 |
| Normal | 0.49 | 0.61 | 0.79 |
| Normal | 0.39 | 0.64 | 0.66 |
| Normal | 0.67 | 0.69 | 0.58 |
| Normal | 0.91 | 0.74 | 0.58 |
| AD | 0.53 | 0.40 | 0.65 |
| AD | 0.15 | 0.41 | 0.55 |
| AD | 0.27 | 0.47 | 0.58 |
| AD | 0.21 | 0.42 | 0.61 |
| AD | 0.38 | 0.44 | 0.62 |
| AD | 0.21 | 0.43 | 0.72 |
| AD | 0.32 | 0.33 | 0.76 |
| AD | 0.39 | 0.30 | 0.67 |
| AD | 0.29 | 0.43 | 0.58 |
| AD | 0.29 | 0.61 | 0.73 |
| PD | 0.74 | 0.64 | 1.07 |
| PD | 0.97 | 0.63 | 1.20 |
| PD | 0.84 | 0.67 | 1.24 |
| PD | 1.06 | 0.61 | 1.15 |
| PD | 0.93 | 0.63 | 1.11 |
| PD | 0.81 | 0.63 | 1.16 |
| PD | 0.80 | 0.64 | 1.09 |
| PD | 0.94 | 0.64 | 1.12 |
| PD | 0.75 | 0.63 | 1.57 |

# References

[1] D. Feng, Z.-Y. Gu, J.-R. Li, H.-L. Jiang, Z. Wei, H.-C. Zhou, *Angew. Chem., Int. Ed.* **2012**, *51* (41), 10307, <https://doi.org/https://doi.org/10.1002/anie.201204475>.

[2] X. Fang, Q. Shang, Y. Wang, L. Jiao, T. Yao, Y. Li, Q. Zhang, Y. Luo, H.-L. Jiang, *Adv. Mater.* **2018**, *30* (7), 1705112, <https://doi.org/https://doi.org/10.1002/adma.201705112>.

[3] D. Chen, Z. Jin, H. Xing, *Langmuir* **2022**, *38* (40), 12292, <https://doi.org/10.1021/acs.langmuir.2c01932>.

[4] D. Kumar, S. N. Sinha, B. Gouda, *J Am Soc Mass Spectrom* **2024**, *35* (4), 663, <https://doi.org/10.1021/jasms.3c00326>.

[5] M. Perry, Q. Li, R. T. Kennedy, *Anal. Chim. Acta* **2009**, *653* (1), 1, <https://doi.org/https://doi.org/10.1016/j.aca.2009.08.038>.

[6] Z. Wu, D. Lin, Y. Li, *Nat Rev Neurosci* **2022**, *23* (5), 257, <https://doi.org/10.1038/s41583-022-00577-6>.
